# A network of transcription factors in complex with a regulating cell cycle cyclin orchestrates fungal oxidative stress responses

**DOI:** 10.1186/s12915-024-01884-3

**Published:** 2024-04-12

**Authors:** Yanze Kan, Zhangjiang He, Nemat O. Keyhani, Ning Li, Shuaishuai Huang, Xin Zhao, Pengfei Liu, Fanqin Zeng, Min Li, Zhibing Luo, Yongjun Zhang

**Affiliations:** 1 Key Laboratory of Agricultural Biosafety and Green Production of Upper Yangtze River (Ministry of Education), College of Plant Protection, Southwest University, Chongqing, 400715 People’s Republic of China; 2 Key Laboratory of Entomology and Pest Control Engineering, Beibei Culture Collection of Chongqing Agricultural Microbiology, Chongqing, 400715 People’s Republic of China; 3 Biochemical Engineering Center of Guizhou Province, Guizhou University, Guiyang, 50025 People’s Republic of China; 4 Department of Biological Sciences, University of Illinois, Chicago, IL 60607 USA

**Keywords:** Transcription factor, Protein complex, Oxidative stress response, Fungal pathogen, Chromatin immunoprecipitation, Gene regulation

## Abstract

**Background:**

Response to oxidative stress is universal in almost all organisms and the mitochondrial membrane protein, BbOhmm, negatively affects oxidative stress responses and virulence in the insect fungal pathogen, *Beauveria bassiana*. Nothing further, however, is known concerning how *BbOhmm* and this phenomenon is regulated.

**Results:**

Three *o*xidative *s*tress *r*esponse regulating Zn_2_Cys_6_ transcription factors (BbOsrR1, 2, and 3) were identified and verified via chromatin immunoprecipitation (ChIP)-qPCR analysis as binding to the *BbOhmm* promoter region, with BbOsrR2 showing the strongest binding. Targeted gene knockout of *BbOsrR1* or *BbOsrR3* led to decreased *BbOhmm* expression and consequently increased tolerances to free radical generating compounds (H_2_O_2_ and menadione), whereas the Δ*BbOsrR2* strain showed increased *BbOhmm* expression with concomitant decreased tolerances to these compounds. RNA and ChIP sequencing analysis revealed that BbOsrR1 directly regulated a wide range of antioxidation and transcription-associated genes, negatively affecting the expression of the *BbClp1* cyclin and *BbOsrR2*. BbClp1 was shown to localize to the cell nucleus and negatively mediate oxidative stress responses. BbOsrR2 and BbOsrR3 were shown to feed into the Fus3-MAPK pathway in addition to regulating antioxidation and detoxification genes. Binding motifs for the three transcription factors were found to partially overlap in the promoter region of *BbOhmm* and other target genes. Whereas BbOsrR1 appeared to function independently, co-immunoprecipitation revealed complex formation between BbClp1, BbOsrR2, and BbOsrR3, with BbClp1 partially regulating BbOsrR2 phosphorylation.

**Conclusions:**

These findings reveal a regulatory network mediated by BbOsrR1 and the formation of a BbClp1-BbOsrR2-BbOsrR3 complex that orchestrates fungal oxidative stress responses.

**Supplementary Information:**

The online version contains supplementary material available at 10.1186/s12915-024-01884-3.

## Background

Organisms, including fungi, both produce compounds, e.g., reactive oxygen species (ROS) as a byproduct of respiration / oxidative phosphorylation that results in oxidative stress, and encounter oxidative stress from the environment. In addition, for microbial pathogens, oxidative stress often occurs as a host defense mechanism during interactions with potential targets. Internal / external sources of ROS have been implicated to act as signal molecules, e.g., nitric oxide (NO), that mediate various signaling events, including cell development, differentiation and proliferation, apoptosis, immunity, and stress responses [1–3]. However, accumulation and/or exposure to ROS is often highly toxic to cells, leading to oxidative damage to DNA, proteins, lipids, and other cellular components, and can activate apoptotic cell death programs [4, 5]. Thus, it is crucial for cells to maintain intracellular oxidative homeostasis for normal development and defense, and to be able to overcome host and environmental sources of oxidative stress, particularly for successful infection of hosts. Generally, intracellular ROS homeostasis is modulated by the activities of antioxidant enzymes (e.g., catalases, peroxidases, superoxide dismutases, and laccases), as well as by non-enzymatic ROS scavengers (e.g., glutathione) [6–9]. With respect to fungal pathogens, a variety of additional non-enzymatic means, including production of small soluble molecules (glutathione) for internal ROS scavenging, synthesis of melanin as a buffer against external ROS, and / or secretion of metabolites (e.g., oxalic acid) to suppress host oxidative burst, are known to contribute to virulence [10–12].

Thus far several partially redundant signaling pathways have been shown to contribute to or regulate fungal oxidative / ROS stress responses [13, 14]. These include the high osmolarity glycerol (Hog1) mitogen-activated protein (MAP) kinase, the protein kinase C-like, PKC1-MAP kinase, and cell wall integrity (CWI) pathways [15–18]. In addition, the cytokinesis-required Cdc14 phosphatase and a P-type calcium ATPase have been shown to affect these signaling pathways in response to oxidative stress [19, 20]. In terms of downstream transcription factors (TFs), a range of oxidative stress response-involved TFs and their gene targets have been characterized. In the yeast *Saccharomyces cerevisiae*, transcriptional regulators YAP1 (AP1), YAP2, Msn2 / Msn4, Skn7, and Sko1, as well as the heat-shock transcription factor Hsf1, are involved in regulation of oxidative defense responses [21, 22]. Similar roles of some of those TF orthologs are reported in the fission yeast *Schizosaccharomyces pombe* and some fungal pathogens, such as *Candida albicans*, *Ustilago maydis* and *Magnaporthe oryza*, *Beauveria bassiana*, and *Metarhizium anisopliae*, although functional divergence of some TFs has been demonstrated in different fungal species [23–37]. However, besides those conserved TFs essentially found both in yeast and filamentous fungi, other TFs participating in the control of oxidative stress responses have been poorly characterized or have yet to be revealed. This is particularly complicated by the existence of over 37 different TF families and an average of ~ 360 different TFs encoded in Ascomycete genomes, that can range from as few as 113 in *S. japonicus* to 1035 in *Nectria heematococca* (*Neurospora crassa* has 321), many of whose functions remain unknown [38].

*B. bassiana* is an economically important insect fungal pathogen that has been used as a microbial agent for control of a variety of insect pests [39, 40]. Of the 168 predicted TFs found in the *B. bassiana* genomes [30], only a handful of TFs have been characterized. *B. bassiana* infects their insect host via direct penetration of the cuticle, where spores attach to the surface, germinate, and penetrate the exoskeleton using a mixture of enzymatic activities, mechanical pressure, and secretion of (toxic) secondary metabolites [41, 42]. Like other microbial pathogens in different host systems (e.g., plant and mammalian pathogens), *B. bassiana* must overcome an insect immune response-generated ROS “oxidative burst” during infection that can directly damage invading fungal cells, and / or function to activate other host immune responses (e.g., stimulate antimicrobial enzyme / peptide synthesis, activate the immune prophenoloxidase system and / or programmed cell death responses) that seek to block further fungal infection and disease progression [43]. Conversely, many microbial pathogens, including fungi, produce ROS as a means for targeting host cells and defenses [2]. Thus, both interacting organisms (the host and the pathogen) seek to utilize oxidative stress to their own advantage while at the same time having to remediate the effects of such stress. One known (fungal) pathway broadly responsive to different stress conditions, including osmotic, oxidative, and heat stresses, is the Hog1 mitogen-activated protein (MAP) kinase pathway, that has also been shown to contribute to virulence in *B. bassiana* [44]. One downstream target gene of the *B. bassiana* Hog1 pathway encodes the mitochondrial membrane protein, BbOhmm, which negatively controls fungal oxidative stress resistance, hypoxia tolerance, and virulence, meaning that a targeted gene knockout mutant, Δ*BbOhmm*, is more resistant to oxidative stress, hypoxia, and shows higher virulence than the wild type strain [2, 43]. However, the Δ*BbOhmm* mutant shows high ROS levels and impaired spore formation, suggesting that BbOhmm acts in an internal ROS production pathway linked to conidiation, and that there exists an antagonistic pleiotropic effect for the protein. Nothing further, however, is known concerning how *BbOhmm* expression and this phenomenon is regulated. Here, we identified three previously uncharacterized Zn_2_Cys_6_ TFs, termed BbOsrR1, BbOsrR2, and BbOsrR3 that regulated *BbOhmm* expression and more broadly participated in the control of *B. bassiana* oxidative stress responses. We further showed the formation of a complex between BbOsrR2, BbOsrR3, and the BbClp1 cyclin, in which BbClp1 partially regulated the phosphorylation of BbOsrR2. Our results provide functions for (orphan) TFs and identify a complex involved in OSRs, demonstrating coordinative regulation of oxidative stress responses via a new regulatory network.

## Results

### Identification of transcription factors regulating BbOhmm

To define the boundaries of the regulatory element in the *BbOhmm* promoter involved in response to oxidative stress, the entire *BbOhmm* open reading frame (ORF) along with different lengths of upstream sequences (1008–2500 bp) were cloned and reintroduced into the (previously constructed) Δ*BbOhmm* strain [2], with functionality of the upstream regulatory region tested by complementation of the Δ*BbOhmm* oxidative stress phenotype. Tolerance of the Δ*BbOhmm* strain to hydrogen peroxide (H_2_O_2_) was restored to wild type levels using at least 1100 bp of upstream promoter (from ATG starts site) sequence but not by sequences containing 1000 bp or less (Additional file 1: Fig S1). To further define the regulatory element, upstream regions corresponding to 1094, 1088, 1082, 1062, and 1008 bp (+ the *BbOhmm* ORF) were transformed into the Δ*BbOhmm* strain. *BbOhmm* with the 1094 bp and 1088 bp constructs, but not with any of the others (e.g., 1082 bp, 1062 bp, and 1008 bp) were able to complement the H_2_O_2_ phenotype of the Δ*BbOhmm* strain to wild type, thus defining the regulatory element to − 1087 to − 1083 bp of sequence upstream of coding region, ‘ATATC’. To determine the contribution of the “ATATC” element, the sequence was mutated to “CCCTC”, resulting in loss of the ability of the promoter to be properly activated and complement the H_2_O_2_ phenotype of the Δ*BbOhmm* strain (Additional file 1: Fig S1).

To isolate potential transcription factors (TFs) that could bind to the *BbOhmm* promoter in response to oxidative stress, yeast-one hybrid screening was performed using as the sequence target, a three-copy tandem repeat of the element “ATATC” fused to the aureobasidin A (AbA) ORF. No background binding to this element in the yeast host strain was observed since the resultant yeast strain could grow on uracil-free SD agar but not on SD agar containing 100–200 ng/ mL AbA. The resultant yeast strain was then transformed and screened with a *B. bassiana* cDNA library. A total of 132 clones with inserts > 200 bp were sequenced, with sequence analysis revealing 7 clones encoding four proteins containing DNA-binding domains (Additional file 1: Fig S2A). Further yeast-one hybrid screening indicated only 3 of the constructs (proteins products) showed binding to the tandem repeat element (Additional file 1: Fig S2B) and could activate transcription (Additional file 1: Fig S2C). Bioinformatic analysis indicated that these proteins were annotated as GAL4-like Zn_2_Cys_6_ proteins (BBA_01499, BBA_04239, and BBA_01981) in the *B. bassiana* genome, consisting of 702, 764, and 1010 amino acids that were designed as BbOsrR1, BbOsrR2, and BbOsrR3 (*O*xidative *s*tress *r*esponse *R*egulators 1, 2, and 3), respectively. BbOsrR1 and BbOsrR3 contained GAL4-like Zn_2_Cys_6_ binuclear cluster DNA-binding domains and fungal transcription factor regulatory middle homology region (one each), whereas BbOsrR2 consisted of a large integument protein UL36-like region (PHA03247) containing a GAL4-like Zn_2_Cys_6_ binuclear cluster (Additional file 1: Fig S2D). Transcription levels of *BbOsrR1* were reduced by 54.1 or 77.9% after exposure of *B. bassiana* to 4 mM H_2_O_2_ or 60 μM menadione (MND) for 30 min as compared to those in the normal culture (¼ SDY), respectively. However, H_2_O_2_ or MND stress resulted in significant increase in *BbOsrR2* expression level (73.5 or 579.3%). *BbOsrR3* expression was slightly decreased after exposure of the fungus to H_2_O_2_ stress but not to MND (Additional file 1: Fig S2E). Construction of green fluorescent protein (eGFP)-tagged fusion proteins for each TF, in which the eGFP protein was fused to the C-terminal of each protein and expressed in the *B. bassiana* wild type strain. The GFP signals in each strain localized in the nucleus of conidia, germlings without or with exposure to oxidative stress (4 mM H_2_O_2_ or 60 μM MND for 30 min), and in vivo blastospores (derived from infected insect) (Additional file 1: Fig S3).

**Fig. 1:**
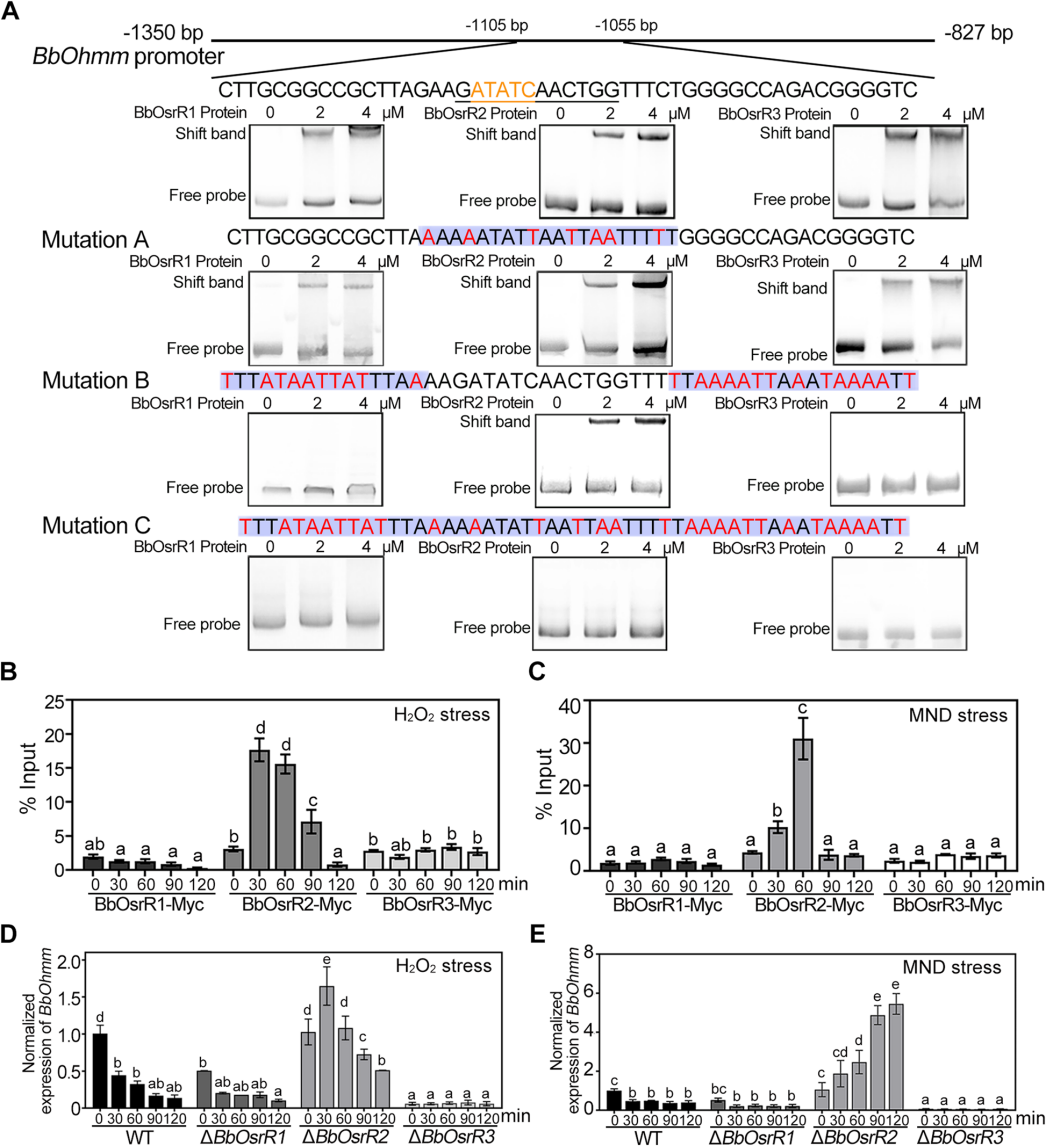
Binding of BbOsrR1, 2, and 3 to *BbOhmm* promoter elements and *BbOhmm* expression in the wild type and mutant strains. **A** EMSA analysis of BbOsrR1, 2, and 3 binding to *BbOhmm* promotor regions. Wild type and three scanning mutant elements (Mutations **A**, **B**, and **C**) were constructed as detailed in the “Methods” section. (**B**-**C**) ChIP-qPCR analysis of BbOsrR1, 2, and 3 binding to the *BbOhmm* promotor region. Fungal cells were grown under no-stress and H_2_O_2_ (4 mM) or menadione (MND, 60 μM)-stress conditions over the indicated time course. **D**,**E** RT-qPCR analysis of *BbOhmm* expression in the wild type and Δ*BbOsrR1*, *2*, and *3* mutant strains grown under no-stress and H_2_O_2_ (4 mM) or menadione (MND, 60 μM)-stress conditions over the indicated time course. Standard deviations (± SD) in **B–E** derived from triplicate experiments. Columns with the same lowercase letters above them are not significantly different (LSD test, *P* < 0.05)

To examine the abilities of the three TFs to bind to the promoter region of *BbOhmm*, electrophoretic mobility shift assays (EMSA) and chromatin immunoprecipitation and quantitative PCR (ChIP-qPCR) assays were performed. EMSA tests indicated that *E. coli*-expressed DNA-binding domains of BbOsrR1, BbOsrR2, and BbOsrR3 could bind to the “ATATC” motif-containing promotor sequence (524 bp) of *BbOhmm* (Fig. 1A). To expand upon the identity of the binding sites for BbOsrR1, 2, and 3, a scanning mutation approach in which sets of the “C” and “G” nucleotides in the sequences surrounding the “ATATC” region were point mutated to (C to T) and (G to A) as follows: (i) Mutation A, − 1091 bp to − 1072 bp upstream of the coding region, 7 mutations in a 20-bp region, (ii) Mutation B, − 1105 bp to − 1090 bp and − 1073 bp to − 1055 bp upstream of “ATG” consisting of 10 changes out of 15 bp at 5′-end and 14 out of 18 bp at the 3′-flanking end, and (iii) Mutation C, containing the point mutations made in A + B in one sequence. EMSA tests were performed using the *E. coli*-expressed DNA-binding domains of BbOsrR1, 2 and 3 with the mutated sequences-contained promoter sequences (524 bp) of *BbOhmm*. All the TF DNA-binding domains could bind to the Mutation A sequence, but only BbOsrR2 bound to the Mutation B sequence, in which a putative Zn_2_Cys_6_ binding sequences “GATATCAACTGG” was found. No binding band was seen between any of the TF binding domains with the Mutation C sequence (Fig. 1A). These results indicated (i) flexibility in the binding of the BbOsrR TFs to the ATATC site (i.e., binding to ATATT sequences) and (ii) contributions of flanking sequences and / or shifted binding sites for the BbOsrR1 and BbOsrR2 TFs.

ChIP-qPCR analysis using Myc-labeled TFs (fusion proteins) in *B. bassiana* revealed low enrichment of BbOsrR1 and BbOsrR3 at the *BbOhmm* promoter, which was not affected by addition of oxidant H_2_O_2_ (4 mM) or MND (60 μM) to the growth media. However, BbOsrR2 was highly enriched in the *BbOhmm* promoter, with increased signal seen over a time course of exposure of cells to H_2_O_2_ or MND peaking at 30–60 min post-stress (Fig. 1B, C). To examine functions of BbOsrR1, BbOsrR2, and BbOsrR3, targeted gene knockout (Δ*BbOsrR1*, Δ*BbOsrR2*, and Δ*BbOsrR3*) and their corresponding complemented strains were constructed (Additional file 1: Fig S4). RT-qPCR analysis revealed that similar patterns of *BbOhmm* expression were seen between the Δ*BbOsrR1* and wild type strains; however, overall expression in the mutant strain was significantly lower than those in the wild type strain when grown in standard media (normal). Expression of *BbOhmm* was slightly induced by H_2_O_2_ but not influenced by MND stress in the Δ*BbOsrR1* strain (Fig. 1D, E). No difference in *BbOhmm* expression was seen between the Δ*BbOsrR2* and wild type strains in standard media; however, *BbOhmm* expression was significantly elevated when Δ*BbOsrR2* cells were stressed by H_2_O_2_ (for 30 min) or MND (increasing from 30 to 120 min) as compared to the wild type strain (Fig. 1D, E), which correlated to the data of BbOsrR2 binding to the *BbOhmm* promoter region in response to both H_2_O_2_ and MND. Expression of *BbOhmm* was almost completely repressed in the Δ*BbOsrR3* mutant under normal and H_2_O_2_ / MND stress conditions (Fig. 1D, E). These data suggest that BbOsrR2 acts as a (strong) negative regulator of *BbOhmm* in response to both H_2_O_2_ and MND, whereas BbOsrR3 acts as a positive regulator of *BbOhmm* expression.

### BbOsrR1, 2, and 3 differentially contribute to oxidative stress responses and virulence

**Fig. 2:**
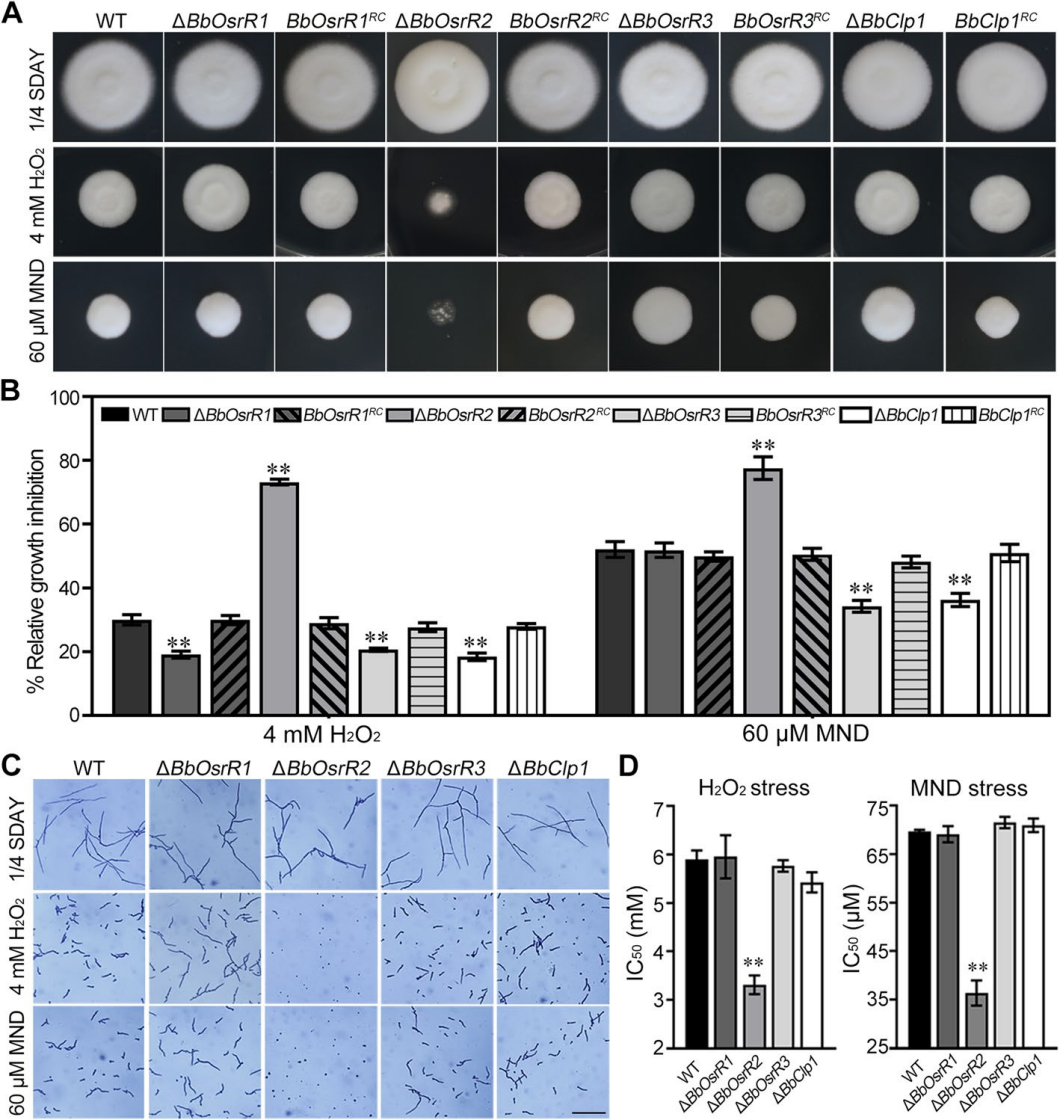
Sensitivities of *B. bassiana* wild type and the mutant strains to H_2_O_2_ and menadione (MND). **A** Vegetative growth of indicated *B. bassiana* strains on ¼ SDAY and ¼ SDAY containing 4 mM H_2_O_2_ or 60 μM MND for 7 days. **B** Calculated relative growth inhibition (RGI) of *B. bassiana* strains challenged with oxidative stressors for 7 days. RGI was calculated as described in “Methods” section. **C** Conidial germination on ¼ SDAY and ¼ SDAY containing 4 mM H_2_O_2_ or 60 μM MND for 24 h. Scale bar = 10 μm. **D** The dose for inhibiting 50% conidial viability (IC_50_) of H_2_O_2_ and menadione. IC_50_ was calculated using the probit regression model and the SPSS 17.0 program. Standard deviations (± SD) of data derived from triplicate experiments are shown in **B** and **D**. The asterisks (**) in the column charts denote *P* < 0.01 for the indicated fungal strains versus the WT (*t*-test), respectively

In terms of their cellular phenotypes, disruption of *BbOsrR1*, *BbOsrR2,* or *BbOsrR3* did not cause any alteration in vegetative growth on the various media tested, including CZA, PDA, and ¼ SDAY, although overall conidial yield was reduced by 18.5–65.2% and 48.0–60.5% in the Δ*BbOsrR1* and Δ*BbOsrR2* strains, respectively, but increased by 38.0–39.4% in the Δ*BbOsrR3* strain (*P* < 0.01). The *BbOsrR2* mutation also showed delayed conidial germination, resulting in an increased median germination time (for 50% of conidial germination, GT_50_) from 11.8 h for the wild type strain to 13.3 h for the Δ*BbOsrR2* strain (*P* < 0.01). However, disruption of *BbOsrR3* promoted conidial germination, resulting in a decreased GT_50_ of 10.6 h (for Δ*BbOsrR3*) versus 11.8 h for the wild type strain (*P* < 0.01) (Additional file 1: Fig S5A-D). Oxidative stress responses of the three mutants were examined on ¼ SDAY. Inactivation of *BbOsrR1* resulted in an increased tolerance to H_2_O_2_ (4 mM), reducing the relative growth inhibition (RGI) in the presence of H_2_O_2_ by 36.4% as compared to the wild type strain (*P* < 0.01), although sensitivity to MND remained unaffected (Fig. 2A, B). In contrast, the Δ*BbOsrR2* strain displayed decreased resistance to both H_2_O_2_ and MND (RGI values increased by 146.8 and 49.0% as compared to the wild type strain, respectively, *P* < 0.01) (Fig. 2A, B). With respect to BbOsrR3, gene disruption led to a significantly increase in tolerances to both H_2_O_2_ and MND (RGI reduced by 31.4 and 34.1%, respectively, *P* < 0.01) (Fig. 2A, B). Moreover, no obvious difference in conidial germination was detected between Δ*BbOsrR1*, Δ*BbOsrR3*, and wild type strains on ¼ SDAY containing different doses of H_2_O_2_ or MND for 24 h. However, Δ*BbOsrR2* conidia displayed dramatically decreased germination when stressed by the two oxidant agents, generating reduced median inhibiting concentrations (IC_50_) from 5.9 mM H_2_O_2_ and 69.7 μM MND for wild type strain to 3.3 mM H_2_O_2_ and 36.4 μM MND for Δ*BbOsrR2* strain, respectively (Fig. 2C, D). Similar results were also examined on CZA containing H_2_O_2_ or MND (Additional file 1: Fig S6A and B), suggesting oxidative stress responses of the three mutant strains were independent of culture media.

**Fig. 3:**
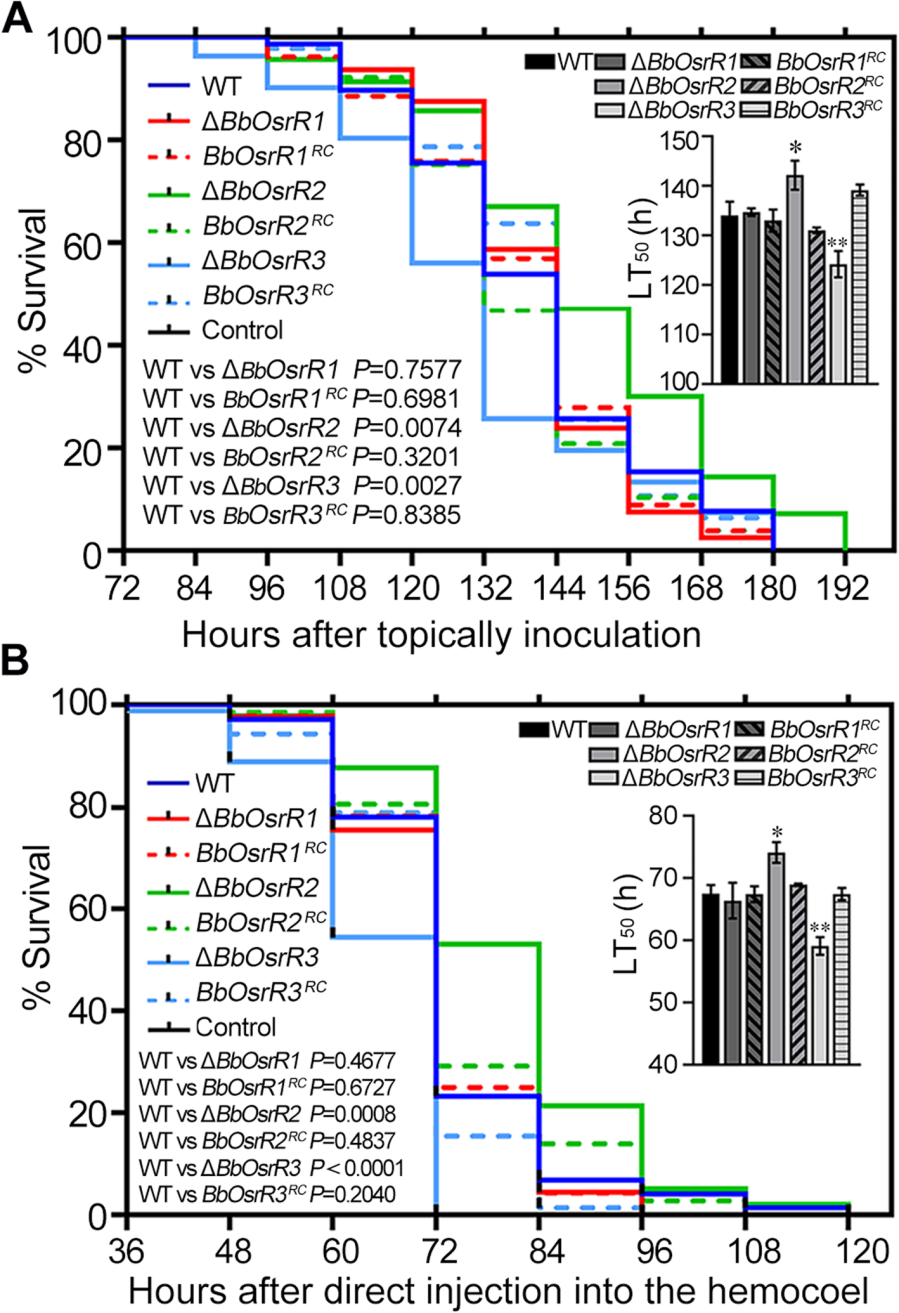
Survival of *Galleria mellonella* larvae with the median lethal time (LT_50_) following topical application by spray of 1 mL conidial suspension (1 × 10^7^ spores/mL) (**A**) and injection into the second proleg with 2 μL of 5 × 10^6^ conidia/mL (**B**). All controls were treated with water. The experiments were repeated twice at different time. The survival data were plotted as Kaplan–Meyer curves and difference of the gene disruption mutant strains or reverse complementation strains from the wild type strain was analyzed using a log-rank test. LT_50_ was calculated using SPSS 17.0 program. The asterisks (*) and (**) in the column charts denote *P* < 0.05 and *P* < 0.01 for the indicated fungal strains versus the WT (*t*-test), respectively

Insect bioassays using *Galleria mellonella* larvae as the host, revealed that loss of *BbOsrR1* did not change *B. bassiana* virulence, but that the Δ*BbOsrR2* mutant was impaired in its ability to infect and kill hosts, resulting in an increased mean lethal time to kill 50% of host insects (LT_50_) from 134.0 ± 2.7 h for wild type strain to 142.2 ± 2.9 h for Δ*BbOsrR2* strain in topical bioassays that reflect the “nature” route of infection (5 × 10^6^ conidia/mL) (*P* < 0.05), and from 67.5 ± 1.1 h for wild type strain to 74.1 ± 1.4 h for Δ*BbOsrR2* strain in intrahemocoel injection assays that bypass the need for cuticle penetration to directly interact with the host (innate) immune system (2 μL 1 × 10^6^ conidia/ mL) (*P* < 0.01). In contrast, the Δ*BbOsrR3* was more virulent than the wild type strain, yielding an LT_50_ = 124.2 ± 2.9 h as compared to wild type strain (134.0 ± 2.7 h) in topical bioassays (*P* < 0.01), and an LT_50_ = 59.1 ± 1.4 h as compared to the wild type strain (67.5 ± 1.1 h) in intrahemocoel injection assays (*P* < 0.01) (Fig. 3). No obvious differences were seen between all the different complement strains and the wild type strain in the phenotypes examined, and unless otherwise noted, all the complemented strains were identical to the wild type strain (Fig. 3).

### BbOsrR1 regulates antioxidation and transcription-involved genes

We then employed RNA sequencing (RNA-seq) and chromatin immunoprecipitation sequencing (ChIP-seq) to identify target genes of BbOsrR1 in response to H_2_O_2_ stress. Global RNA-seq analysis of the Δ*BbOsrR1* / wild type comparison revealed 1213 differentially expressed genes (DEGs, 695 showing increased expression and 518 showing decreased expression, fold change > 2, *P* < 0.001) in standard media (¼ SDY) and 1442 (688 increased / 754 decreased) in ¼ SDY + H_2_O_2_ (4 mM for 30 min) (Additional file 2: Table S2-S3). The number of upregulated and downregulated DEGs under non-stress conditions and H_2_O_2_-stress conditions are summarized in Table 1 with GO enrichment analysis of DEG datasets shown in Additional file 1: Fig S7. RT-qPCR analysis showed that transcription levels of three fungal central development pathway (CDP) genes, *BrlA* (BBA_07544), *AbaA* (BBA_00300), and *VosA* (BBA_01023), which are present in the downregulated ¼ SDY DEG dataset, were significantly decreased at conidiation stage (5 days on ¼ SDY agar, ¼ SDAY). Another CDP gene, *WetA* (BBA_06126), was also significantly downregulated in the Δ*BbOsrR1* strain at this stage (Additional file 1: Fig S5E). To evaluate expression patterns of antioxidant / detoxification DEGs from RNA-seq datasets in minimal medium CZB, five genes were selected and analyzed using RT-qPCR. Expression levels of all the genes were significantly upregulated in the CZB-cultured Δ*BbOsrR1* cells either under no-stress or H_2_O_2_ (4 mM for 30 min)-stress condition as compared to wild type strain (Additional file 1: Fig S6C), which were consistent with their expression patterns in the nutrient-rich ¼ SDY-derived RNA-seq data (Additional file 2: Table S2-S3).

**Table 1:** Datasets summary of RNA-seq and ChIP-seq

	Δ*BbOsrR1*	Δ*BbOsrR2*	Δ*BbOsrR3*
RNA-seq^a^			
Normal	695 up/518 down	616 up/344 down	1016 up/783 down
H_2_O_2_ stress	630 up/712 down	318 up/369 down	324 up/ 311 down
Menadione stress	/	346 up/508 down	830 up/954 down
ChIP-seq			
Normal	340	1109	43
H_2_O_2_ stress	40	926	36
Menadione stress	/	285	56
Direct targets genes (In both RNA-seq and ChIP-seq datasets)			
Normal	41 up/19 down	94 up/39 down	8 up/2 down
H_2_O_2_ stress	6 up	84 up/62 down	5 up/2 down
Menadione stress	/	10 up/54 down	10 up/4 down
Additional information			
Putative binding site sequence	BGGYGRCGGHGGCGG	CKYCGBSGYCG	CCYCSSYRAC
Binding site verified (EMSA)	*CatA* (BBA_06186), *BbOsrR2* (BBA_04239), *BbClp1* (BBA_07582)	*Msg5* (BBA_09275), hypothetical protein gene (BBA_06338), *Mcm1* (BBA_06763)	*Fus3* (BBA_01244), hypothetical protein gene (BBA_06338)

^a^As compared to the wild type strain

**Fig. 4:**
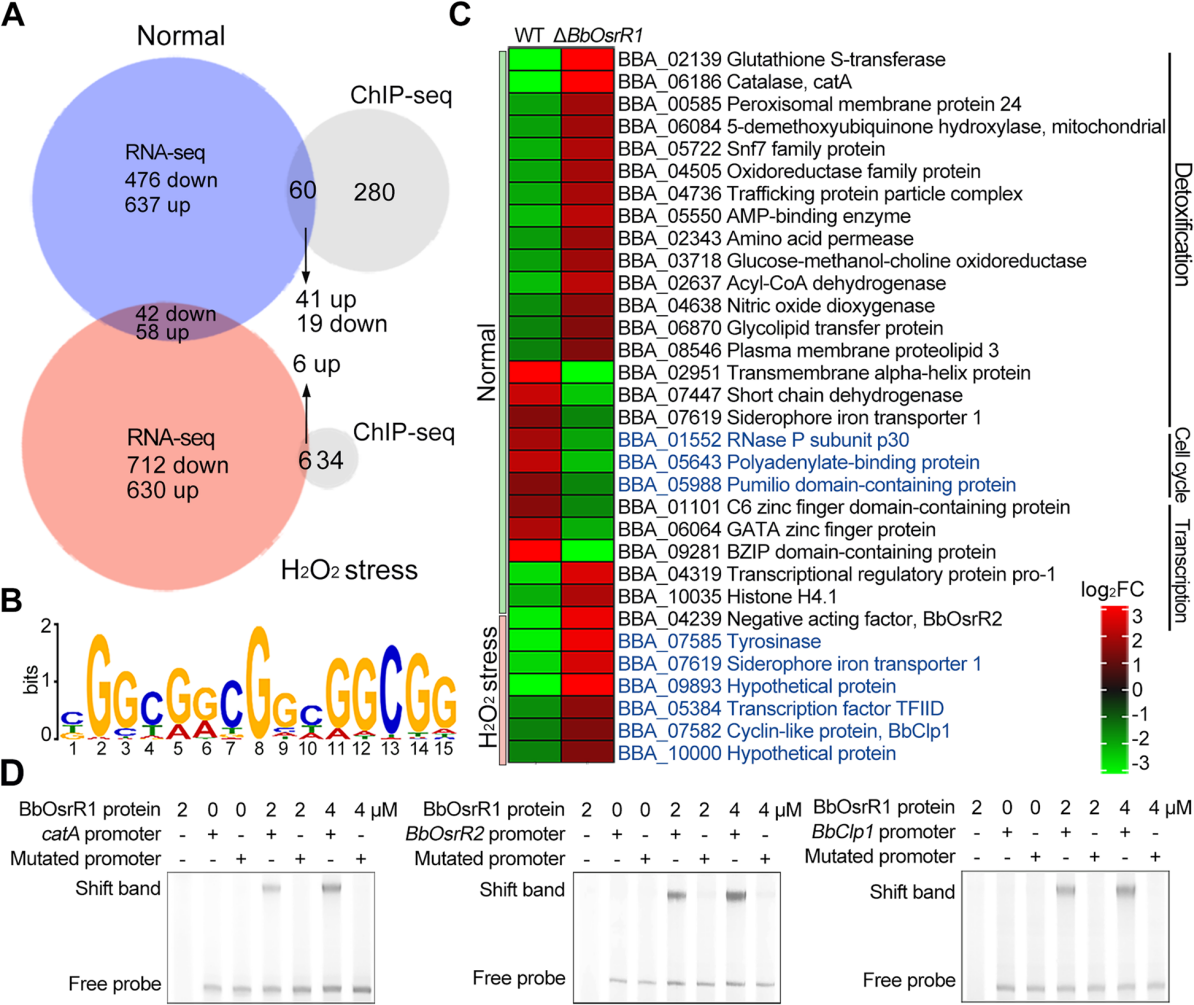
ChIP-seq and RNA-seq analysis identify BbOsrR1 target genes. **A** Identification of BbOsrR1 (gene promoter) targets by comparison of RNA-seq and ChIP-seq under the normal and H_2_O_2_ (4 mM)-stressed conditions for 30 min. **B** MEME analysis indicating the likely binding sequence of BbOsrR1. **C** Partial annotation of identified BbOsrR1 gene targets and their expression patterns. **D** EMSA verification of three identified gene targets of BbOsrR1: *CatA*, *BbOsrR2*, and *BbClp1* with indicated BbOsrR1 protein concentrations. The promoter region of each target harboring mutated binding motif was used as the scrambled DNA sequence control (Mutated promoter) as detailed in the “Methods” section

To probe targets of BbOsrR1, ChIP-seq using a *B. bassiana* strain expressing Myc-tagged BbOsrR1 (probed with an anti-Myc antibody) was performed to compare growth of the Δ*BbOsrR1* mutant in ¼ SDY to growth in ¼ SDY + H_2_O_2_ stress. These analyses revealed a total of 460 unique ChIP-seq peaks that mapped to 340 different target genes (within 1.5-kb sequences upstream of an ORF start codon) in ¼ SDY dataset, whereas only 40 target genes of BbOsrR1 were identified within 65 ChIP-seq peaks in promoter regions in ¼ SDY + H_2_O_2_ dataset (Fig. 4A, Additional file 2: Table S13-S14). MEME analysis revealed an identifiable binding sequence for BbOsrR1 in ChIP-seq peaks, as BGGYGRCGGHGGCGG with highest E value (2.2e − 4) and frequency (205/525) (Fig. 4B and Table 1). Cross analysis of the ChIP-seq with the RNA-seq datasets revealed 60 co-identified BbOsrR1 target genes (17.6%) in the standard media dataset and 6 BbOsrR1 target genes (15%) in the H_2_O_2_ stress dataset (Fig. 4C and Table 1). With respect to the former (60), target genes were enriched in antioxidant and detoxification-associated function (e.g., glutathione S-transferase, catalase (*catA*), oxidoreductase family protein, trafficking protein particle complex, AMP-binding enzyme, peroxisomal membrane protein 24, and nitric oxide dioxygenase), as well as transcripts involved in cell cycle and transcription. Many of these targets involved in antioxidant / detoxification were significantly upregulated in the Δ*BbOsrR1* strain, supporting its role as a repressor of oxidative response pathway components. Expression of *BbOsrR2* was identified as a potential direct target of BbOsrR1 (Fig. 4C); however, overall expression of *BbOsrR2* was not significantly affected in the Δ*BbOsrR1* strain (a slight 1.45-fold decrease that did not meet the cut-off for significance, Additional file 2: Table S13). Of the six putative direct target genes of BbOsrR1 under H_2_O_2_ stress, all were significantly upregulated in the Δ*BbOsrR1* strain, which encoded the Clp1 cyclin-like protein (BBA_07582, termed BbClp1), transcription factor TFIID (BBA_05384), tyrosinase (BBA_07585), siderophore iron transporter 1 (BBA_07619), and two hypothetical proteins (BBA_09893 and BBA_10000) (Additional file 2: Table S14). Binding of purified BbOsrR1 to the promoter regions of three identified putative target genes, including *CatA*, *BbClp1*, and *BbOsrR2*, was verified by EMSA using respective promoter fragments from each gene and the purified protein. These bindings were not affected by addition of the mutated promoter regions of each target, in which the binding motifs “CCCGTCGCGTGGCGG” of *CatA* (at − 672 to − 657 bp), “CCGATTGGGTCGGCGG” of *BbOsrR2* (at − 586 to − 570 bp), and “CATTACTCGAGGCGG” of *BbClp1* (at − 1077 to − 1062 bp) promoters were mutated to “TTTATTATATAATAA,” “TTAATTAAATTAATAA,” and “TATTATTTAAAATAA,” respectively (Fig. 4D).

### The BbOsrR1 target gene, BbClp1, negatively mediates oxidative stress response

**Fig. 5:**
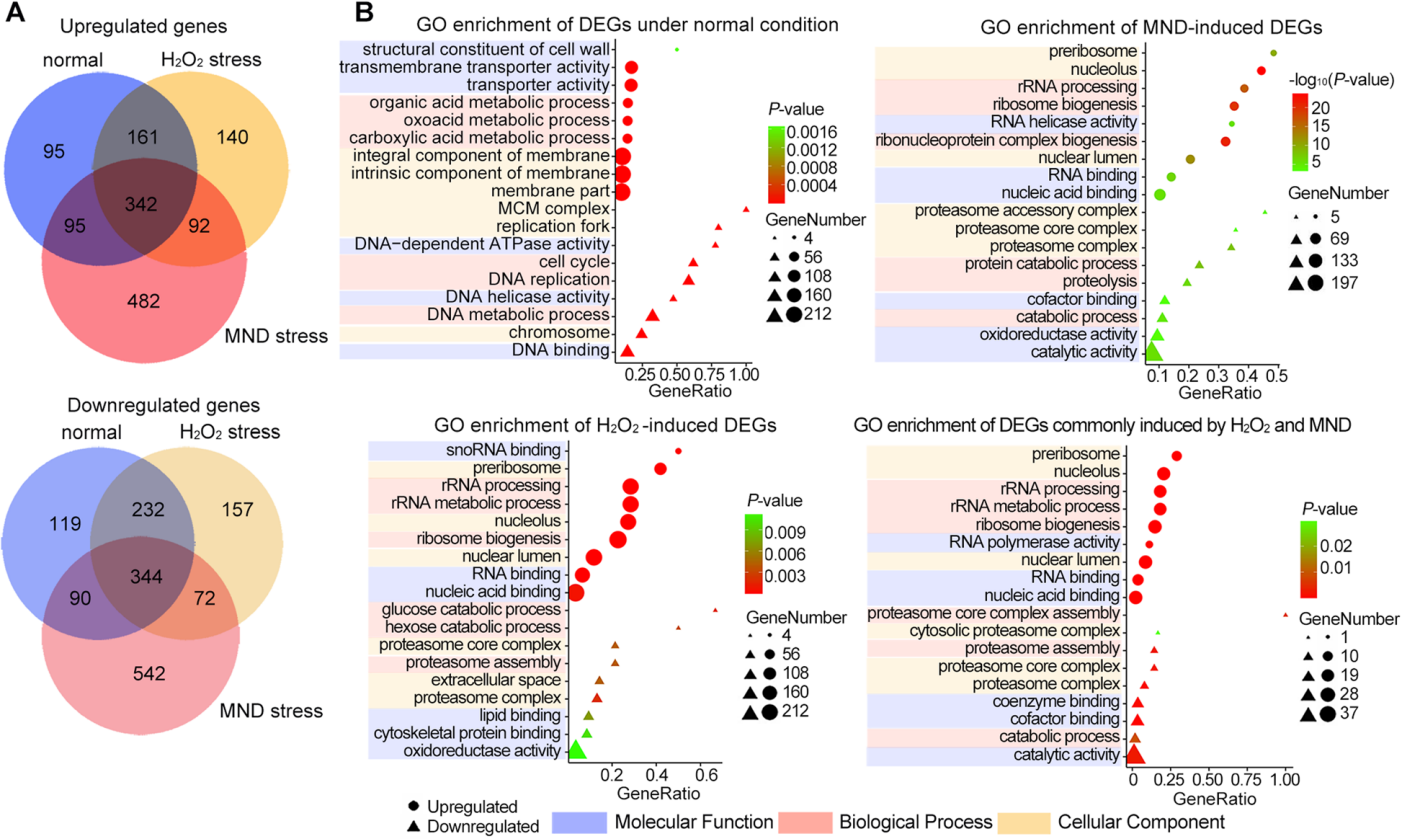
Transcriptomic analysis of the differently expressed genes (DEGs) in the Δ*BbClp1* strain versus the wild type strain under the normal and oxidative stress (4 mM H_2_O_2_ or 60 μM menadione, MND) conditions for 30 min. **A** Venn diagram of DEGs under the normal and oxidative stress conditions. **B** GO enrichment of H_2_O_2_ and MND-induced DEGs as compared to those DEGs under the normal condition

Bioinformatic analysis indicated that *BbClp1* encodes a protein containing a cyclin box domain. The gene expression was not affected by oxidative stress as compared to that of the normal cultures from ¼ SDY (Additional file 1: Fig S2E). Construction and expression of a *BbClp1::eGFP* fusion reporter indicated the expected nuclear localization of BbClp1 in all the cells, including conidia, germlings without or with exposure to oxidative stress (4 mM H_2_O_2_ or 60 μM MND for 30 min), and in vivo blastospores (Additional file 1: Fig S3). To further investigate the role of BbClp1 in oxidative stress response, we constructed a target gene disruption strain Δ*BbClp1* and complemented strain Δ*BbClp1*^*RC*^ (Additional file 1: Fig S4). No significant effects in terms of vegetative growth or virulence were seen in the Δ*BbClp1* mutant as compared to the wild type strain (Additional file 1: Fig S5A and B, and Fig S8). However, the strain displayed decreased conidial yield (by 21.8–22%, *P* < 0.01) and delayed conidial germination, with the GT_50_ increasing from 11.8 h for the wild type strain to 12.7 h for the Δ*BbClp1* strain (*P* < 0.01) (Additional file 1: Fig S5C and D). The Δ*BbClp1* strain also showed increased tolerances to H_2_O_2_ (4 mM) and MND (60 μM) (RGI decreased by 44.4 and 30.4% as compared to the wild type strain, respectively, *P* < 0.01, Fig. 2A, B). However, no obvious difference in conidial germination was examined between Δ*BbClp1* and wild type strains on ¼ SDAY containing different doses of H_2_O_2_ or MND for 24 h (Fig. 2C, D). To examine the underlying mechanism of BbClp1 mediated oxidative responses, RNA-seq was used to compare gene expression patterns between the Δ*BbClp1* mutant and wild type strains under the normal (¼ SDY), H_2_O_2_ (4 mM), and MND (60 μM) stress conditions (30 min). Totals of 1478, 1540, and 2059 DEGs (fold change > 2, *P* < 0.001) were found in comparisons between Δ*BbClp1*^¼SDY^/wild type^¼SDY^, Δ*BbClp1*^H2O2^/wild type^H2O2^, and Δ*BbClp1*^MND^/wild type^MND^, respectively (Additional file 2: Table S4-S6). The DEGs identified by comparing the Δ*BbClp1* mutant grown in ¼ SDY to stress conditions indicated 461 (229 upregulated) and 1118 (614 upregulated) DEGs in the H_2_O_2_ and MND stress condition datasets, respectively. A total of 164 DEGs (92 upregulated and 72 downregulated) DEGs were shared between the Δ*BbClp1* H_2_O_2_ and MND datasets (Fig. 5A). A total of 140 upregulated DEGs were found unique to H_2_O_2_ stress, and 482 unique to MND stress. Major categories of those H_2_O_2_ and MND DEGs included ribosome biogenesis, RNA processing and metabolism, and cellular component biogenesis (Fig. 5B). A total of 157 downregulated DEGs uniquely found in the H_2_O_2_-stress dataset were enriched in sugar (e.g., glucose, hexose, and monosaccharide) catabolism, protein catabolism and function, and cellular process (Fig. 5B), whereas a total of 542 downregulated DEGs uniquely found in the MND dataset were associated with protein catabolism, protein modification and function, and macromolecule catabolism (Fig. 5B). It was noticed that *BbOhmm* was significantly upregulated in the Δ*BbClp1* strain (53-, 22-, and 15-fold in ¼ SDY, ¼ SDY + H_2_O_2_, and ¼ SDY + MND, respectively) (Additional file 2: Table S4-S6). RT-qPCR analysis revealed that transcription levels of two CDP genes, *WetA* and *VosA*, in which the latter present in the ¼ SDY DEG dataset (Additional file 2: Table S4), were significantly decreased in the Δ*BbClp1* strain at conidiation stage (5 days on ¼ SDAY) (Additional file 1: Fig S5E). Moreover, expression levels of several antioxidant / detoxification genes were significantly upregulated in the Δ*BbClp1* cells cultured in CZB or CZB containing H_2_O_2_ (4 mM) or MND (60 μM) for 30 min as compared to those in wild type strain (Additional file 1: Fig S6C), which were in line with their expression patterns in the ¼ SDY-derived RNA-seq data (Additional file 2: Table S4-S6).

### BbOsrR2 controls cell detoxification genes and negatively regulates the Fus3 MAPK pathway

Similar to experiments probing targets of BbOsrR1, ChIP-seq, and RNA-seq were used to isolate targets of BbOsrR2 regulation. RNA-seq analysis revealed that loss of *BbOsrR2* led to total of 960 (616 downregulated), 971 (472 downregulated), and 1051 (572 downregulated) DEGs when compared to the wild type strain (fold change > 2, *P* < 0.001) under no-stress (¼ SDY), H_2_O_2_, and MND-stress conditions, respectively (Additional file 2: Table S7-S9). The number of upregulated / downregulated DEGs under no-stress condition and specifically induced by H_2_O_2_- and MND stress are summarized in Table 1 and GO enrichment analysis of those DEG datasets are shown in Additional file 1: Fig S7. RT-qPCR analysis revealed that transcription levels of three CDP genes, *AbaA*, *WetA*, and *VosA*, in which *WetA* present in the downregulated ¼ SDY DEG dataset (Additional file 2: Table S7), were significantly decreased in the Δ*BbOsrR2* strain at conidiation stage (5 days on ¼ SDAY) (Additional file 1: Fig S5E). In addition, expression of five antioxidant / detoxification genes were dramatically repressed in the Δ*BbOsrR2* cells cultured in CZB or CZB containing H_2_O_2_ (4 mM) or MND (60 μM) for 30 min as compared to those in wild type strain (Additional file 1: Fig S6C), which were in line with their expression patterns in the ¼ SDY-derived RNA-seq data (Additional file 2: Table S4-S6).

**Fig. 6:**
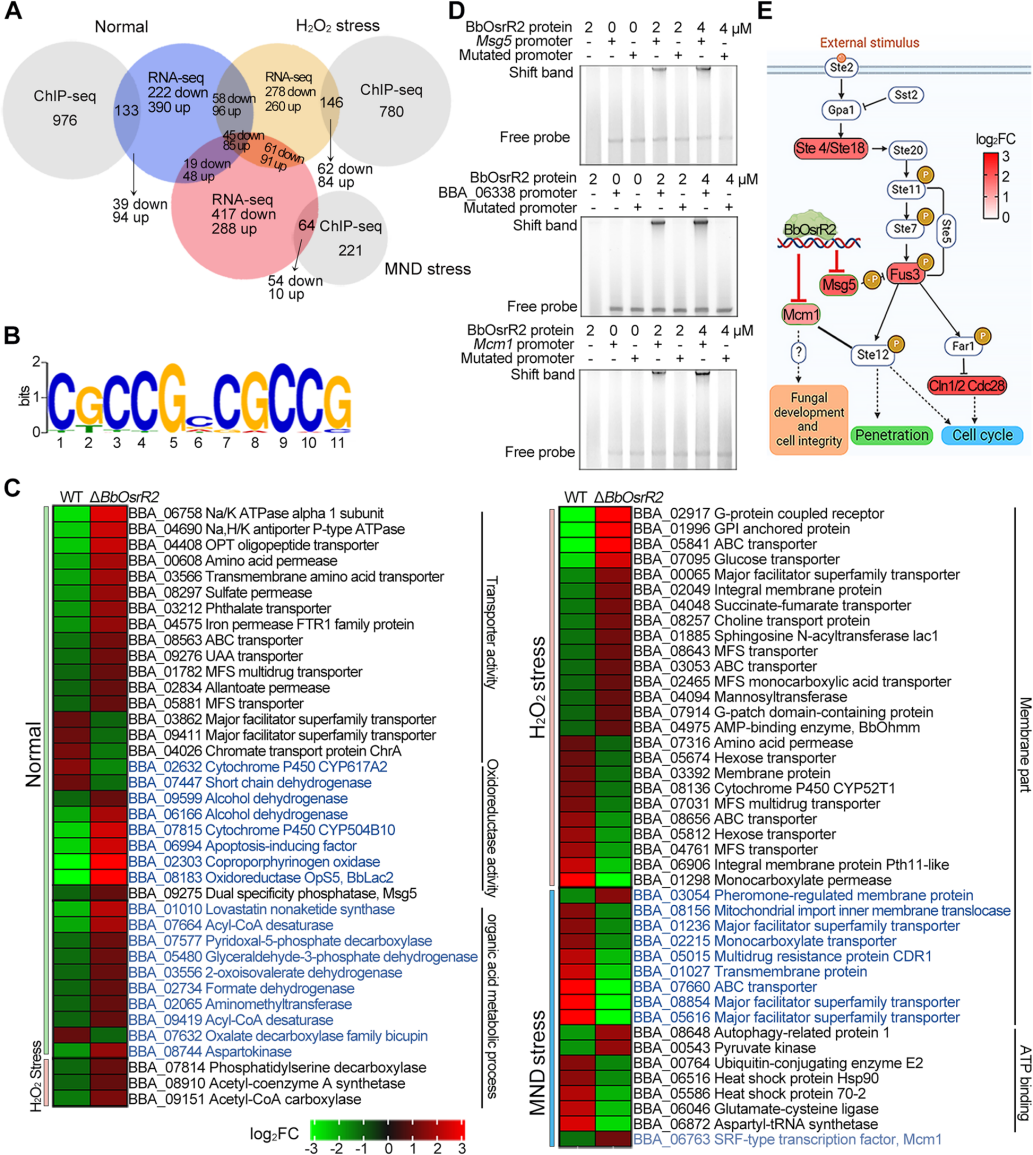
ChIP-seq and RNA-seq analysis identify BbOsrR2 target genes. **A** Identification of BbOsrR2 (gene promoter) targets by comparison of RNA-seq and ChIP-seq under the normal, H_2_O_2_ (4 mM), and menadione (MND, 60 μM)-stressed conditions for 30 min. **B** MEME analysis indicating the likely binding sequence of BbOsrR2. **C** Partial annotation of identified BbOsrR2 gene targets and their expression patterns. **D** EMSA verification of three BbOsrR2 targets: *Msg5*, a hypothetical protein gene (BBA_06338) and *Mcm1* with indicated BbOsrR2 protein concentrations. The promoter region of each target harboring mutated binding motif was used as the scrambled DNA sequence control (Mutated promoter) as detailed in the “Methods” section. **E** Model of BbOsrR2 regulation of the Fus3-MAP kinase pathway via (negative) targeting of *Msg5* and *Mcm1*, affecting expression of the *Ste4*, *Fus3*, and *Cdc28* genes. Upregulated genes in the Δ*BbOsrR2* mutant are shadowed in red

To identify direct promoters / target genes of BbOsrR2 in response to oxidative stress, BbOsrR2 binding sites were detected using a global genome-wide ChIP-seq screen with a *B. bassiana* strain expressing a Myc-tagged-BbOsrR2 fusion protein. This screening yielded a total of 1389, 1106, and 503 ChIP-seq peaks (within 1.5 kb nucleotides upstream of an ORF, *P* < 0.001) in cells grown in SDY, ¼ SDY + H_2_O_2_, and ¼ SDY + MND, which were mapped to 1109, 926, and 285 different genes (Additional file 2: Table S15-S17), respectively. Comparison of the ChIP-seq to the RNA-seq data revealed 133 (12%), 146 (15.8%), and 64 (22.5%) target genes found in all datasets for the ¼ SDY, ¼ SDY + H_2_O_2_, and ¼ SDY + MND conditions, respectively. Of these, 94 / 39, 84 / 62, and 10 / 54 targets were significantly upregulated / downregulated in the Δ*BbOsrR2* strain under the three conditions, respectively (Fig. 6A and Table 1). MEME analysis revealed a putative identifiable binding sequence for BbOsrR2 from the ChIP-seq data to be CKYCGBSGYCG with highest *E* value (3.7e − 5) and frequency (267 / 2958) (Fig. 6B and Table 1). Target genes under the no-stress (¼ SDY) condition were mainly associated with transporter activity, oxidoreductase activity, and organic acid metabolic processes, in which ~ 83% (29 / 35) were significantly upregulated in the Δ*BbOsrR2* strain (Fig. 6C). One target was the dual specificity phosphatase gene, *Msg5* (BBA_09275), that was significantly upregulated in the Δ*BbOsrR2* mutant (Fig. 6C and Additional file 2: Table S15). Yeast Msg5 specifically dephosphorylates threonine and tyrosine residues in the Mpk1 and Fus3/Kss1 MAP kinases to regulate cell wall integrity (CWI) and pheromone responses, respectively [45]. Examination of the RNA-seq dataset revealed that the Fus3 pathway genes, *Ste4* (BBA_03738), *Fus3* (BBA_01244), and *Cdc28* (BBA_02861), were significantly upregulated in the Δ*BbOsrR2* strain, suggesting participation of BbOsrR2 in the Fus3 MAPK pathway as a negative regulator (Fig. 6E). When fungal cells were stressed by H_2_O_2_, most BbOsrR2 targets (25 / 28) were distributed in terms corresponding to membrane functioning and organization, with 15 and 10 genes significantly upregulated and downregulated, respectively, in the Δ*BbOsrR2* strain, that mainly included transporter and detoxification genes. In addition, three signaling pathway-involved genes were identified, including two upregulated genes, encoding a G-protein-coupled receptor (BBA_02917) and a G-patch domain-containing protein (BBA_07914), respectively, and one downregulated gene, namely, an integral membrane Pth11-like (BBA_06906) gene. *BbOhmm* was also identified as a target that was significantly upregulated in the Δ*BbOsrR2* strain (Fig. 6C and Additional file 2: Table S16). Under the MND-stress condition, targets were enriched in membrane activity and functioning (transporter activity), and ATP binding activity, in which 15 of 17 targets were found to be downregulated in the Δ*BbOsrR2* mutant (Fig. 6C and Additional file 2: Table S17). These data were consistent with the decreased tolerance of Δ*BbOsrR2* strain to oxidative stress. One pheromone-regulated membrane protein (BBA_03054) and one SRF-type transcription Mcm1 (BBA_06763) genes were significantly upregulated, in which the former is involved in the Fus3-MAPK pathway, while the latter functions in cell cycle and stress response [46]. Binding of purified BbOsrR2 to the promoter regions of three identified putative target genes, including *Msg5*, a hypothetical protein gene (BBA_06338), and *Mcm1* was verified by EMSA using respective promoter fragments from each gene and the purified protein. These bindings were not affected by addition of the mutated promoter regions of each target, in which the motifs “CCGCCTCTTCCC” of *Msg5* (at − 893 to − 881 bp), “GCAAGGCCGTGTG” of *Mcm1* (at -439 bp to -426 bp), and “GTCTCGCCGACTGT” of BBA_06338 (at − 1972 bp to − 1958 bp) promoters were mutated to “TTATTTTTTTTT,” “ATAAAATTATATA,” and “ATTTTATTAATTAT,” respectively (Fig. 6D).

### BbOsrR3 regulates oxidative response genes and the Fus3-MAPK pathway

**Fig. 7:**
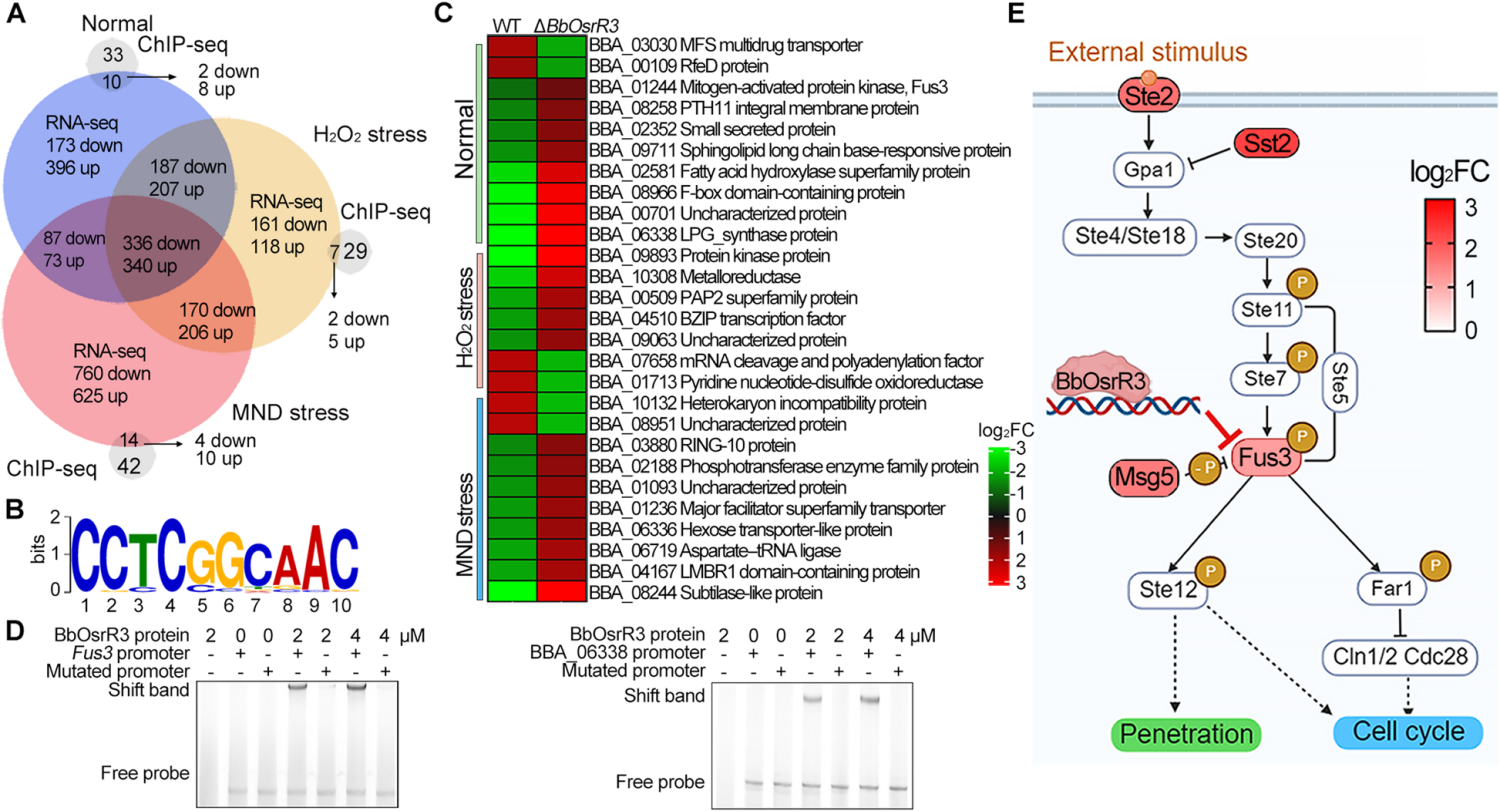
ChIP-seq and RNA-seq analysis identify BbOsrR3 target genes. **A** Identification of BbOsrR3 (gene promoter) targets by comparison of RNA-seq and ChIP-seq under the normal, H_2_O_2_ (4 mM), and menadione (MND, 60 μM)-stressed conditions for 30 min. **B** MEME analysis indicating the likely binding sequence of BbOsrR3. **C** Partial annotation of identified BbOsrR3 gene targets and their expression patterns. **D** EMSA verification of two identified BbOsrR3 targets: *Fus3* and a hypothetical protein gene (BBA_06338) with indicated BbOsrR3 protein concentrations. The promoter region of each target harboring mutated binding motif was used as the scrambled DNA sequence control (Mutated promoter) as detailed in the “Methods” section. **E** Model of BbOsrR3 regulation of the Fus3-MAP kinase pathway via (negatively) targeting *Fus3* and affecting expression of the *Ste2*, *Sst2*, and *Msg5* genes. Upregulated genes in the Δ*BbOsrR3* mutant are shadowed in red

Similar to the experiments performed for BbOsrR1 and BbOsrR2, RNA-seq, and ChIP-seq were used to identify BbOsrR3 target genes. RNA-seq experiments revealed 1799 (1016 / 783, upregulated / downregulated), 1725 (871 / 854), and 2620 (1243 / 1377) DEGs (fold change > 2, *P* < 0.001) in the Δ*BbOsrR3* mutant when compared to the wild type strain during growth in ¼ SDY, ¼ SDY + H_2_O_2_, and ¼ SDY + MND, respectively (Fig. 7A). The number of upregulated and downregulated DEGs under no-stress condition, specifically induced by H_2_O_2_ stress and MND stress, are summarized in Table 1 with GO enrichment analysis of these DEGs shown in Additional file 1: Fig S7. *BbOhmm* was significantly downregulated in the Δ*BbOsrR3* strain (30, 21, and 13-fold under ¼ SDY, ¼ SDY + H_2_O_2_, and ¼ SDY + MND conditions, respectively (Additional file 2: Table S10-S12). RT-qPCR analysis revealed that transcription levels of CDP gene, *AbaA*, were significantly increased but *VosA* was decreased in the Δ*BbOsrR3* strain at conidiation stage (5 days on ¼ SDAY) (Additional file 1: Fig S5E), which was in line with their transcription patterns in the ¼ SDY DEG dataset (Additional file 2: Table S10). Moreover, expression levels of several antioxidant / detoxification genes were significantly upregulated in the Δ*BbOsrR3* cells cultured in CZB or CZB containing H_2_O_2_ (4 mM) or MND (60 μM) for 30 min as compared to those in wild type strain (Additional file 1: Fig S6C), which were consistent with their expression patterns in the ¼ SDY-derived RNA-seq data (Additional file 2: Table S10-S12).

BbOsrR3 binding sites were identified via ChIP-seq using a *B. bassiana* strain expressing Myc-tagged BbOsrR3, yielding 47 (that could be mapped to 43 different genes), 44 (mapped to 36), and 75 (mapped to 56) ChIP-seq peaks (within 1.5 kb nucleotides upstream of an ORF, *P* < 0.001) when cells were grown in ¼ SDY, ¼ SDY + H_2_O_2_, and ¼ SDY + MND, respectively (Additional file 2: Table S18-S20). Comparison of the ChIP-seq with the RNA-seq datasets indicated expression of 10 (8 upregulated / 2 downregulated, 23.3% of the total ChIP sets), 7 (5 / 2, 19.4%), and 14 (10 / 4, 25.0%) genes as BbOsrR3 dependent under the three conditions, respectively (Fig. 7A and summarized in Table 1). MEME analysis indicated the putative DNA-binding sequence for BbOsrR3 to be CCYCSSYRAC with highest E value (8.8e − 3) and frequency (53/166) (Fig. 7B and Table 1). Many upregulated targets were involved in signal transduction and regulator (e.g., Fus3 MAPK, PTH11 integral membrane protein, F-box domain-containing protein, protein kinase protein, bZIP transcription factor, and LMBR1 domain-containing protein), lipid metabolism (sphingolipid long chain-base responsive protein, fatty acid hydroxylase superfamily protein, and phosphotransferase enzyme family protein), cell resistance and detoxification (LPG synthase protein, major facilitator superfamily transporter and metalloreductase), xylose / nitrogen source utilization and virulence (hexose transportor-like protein, small secreted protein, PAP superfamily protein and subtilase-like protein), and vascular remodeling (ring 10 protein) (Fig. 7C and Additional file 2: Table S10-S12). These data were consistent with the increased tolerance to oxidative stress and virulence seen for the Δ*BbOsrR3* strain. Two putative targets, including *Fus3* (BBA_01244) and a hypothetical protein gene (BBA_06338), were also confirmed by EMSA using respective promoter fragments from each gene and the purified BbOsrR3 protein. These bindings were not affected by addition of the mutated promoter regions of each target, in which the motifs “GCGCCGGCGG” of *Fus3* (at − 1583 bp to − 1573 bp) and “GTCTCGCCGACTGT” of BBA_06338 (the target shared with BbOsrR2, at − 1972 bp to − 1958 bp) promoters were mutated to “ATATTAATAA” and “ATTTTATTAATTAT,” respectively (Fig. 7D). The Fus3-MAP kinase gene (*Fus3*) and other three components of the Fus3-MAPK pathway, including the Ste2 G protein-coupled receptor, the Sst2 negative regulator (negatively regulates the G-protein a subunit, Gpa1), and the Msg5 phosphatase genes [47, 48], were significantly upregulated by 2.19–2.73-fold, 3.18–4.04-fold, 4.28–4.69-fold, and 2.89–4.39-fold in the Δ*BbOsrR3* strain during growth ¼ SDY, ¼ SDY + H_2_O_2_, and ¼ SDY + MND, respectively (Fig. 7D and Additional file 2: Table S10-S12). These results suggest that BbOsrR3 regulates the Fus3-MAPK pathway via targeting the Fus3-MAPK gene (Fig. 7E).

### BbOsrR1, 2, and 3 orchestrate expression of oxidative stress response genes

**Fig. 8:**
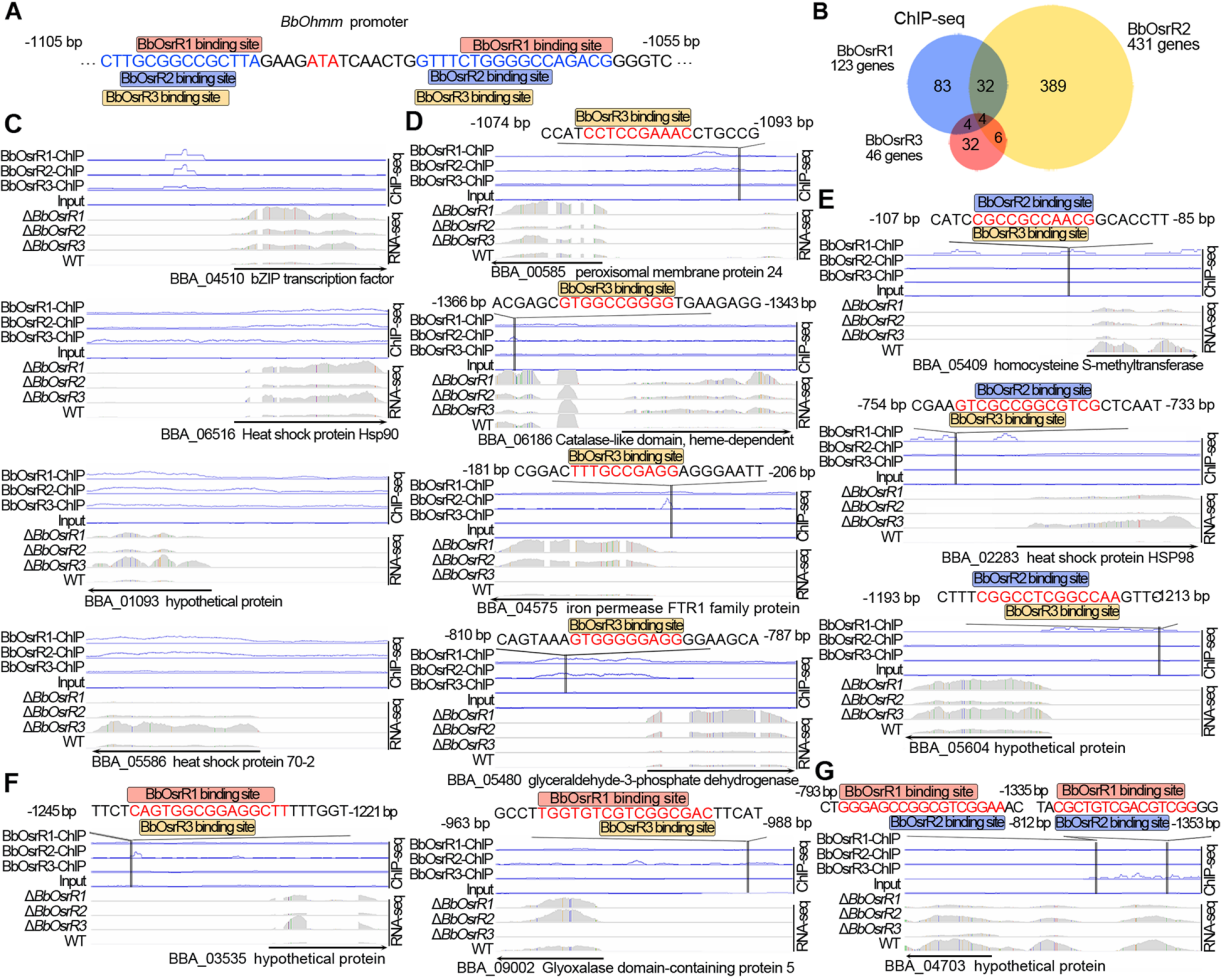
Binding motifs BbOsrR1, 2, and 3 partially overlap in some target promoters. **A** Partial overlap of the binding motifs for BbOsrR1, 2, and 3 in the *BbOhmm* promoter region. **B** Comparative analysis of the targets of BbOsrR1, 2, and 3. **C** Overlap of ChIP-seq peaks for BbOsrR1, 2, and 3 in the promoters of four target genes. **D** BbOsrR3 binding motifs present in promoters of some common targets of BbOsrR1 and BbOsrR2 with partial overlap of their ChIP-seq peaks each other. **E** BbOsrR2 and BbOsrR3-binding motifs presented in promoters of some BbOsrR1 targets with partial overlap of their ChIP-seq peaks each other. **F** BbOsrR1 and BbOsrR3-binding motifs present in promoters of some BbOsrR2 targets with partial overlap of their ChIP-seq peaks each other. **G** BbOsrR1 and BbOsrR2-binding motifs present in promoters of some BbOsrR3 targets with partial overlap of their ChIP-seq peaks each other

Promoter element scanning of upstream sequences of *BbOhmm* ORF revealed two binding motifs for each of BbOsrR1, BbOsrR2, and BbOsrR3 present at − 1092 to − 1102 bp and − 1077 to − 1055 bp upstream of ORF, respectively, which flanked the 5′- and 3′-ends of the “ATATC” sequence that was identified as important for mediating regulation of *BbOhmm* above. The binding motifs for the three TFs partially overlapped within the two regions (Fig. 8A). However, it was confusing that distribution of the TF binding motifs were different from the yeast-one hybrid screening using a three-copy tandem repeat of the “ATATC” element as the target sequence which seemed to need for regulation of *BbOhmm*. To figure out the puzzle, we scanned the three-copy tandem repeat of the “ATATC” used for construction of pAbAi-bait and found the binding motifs of the three TFs (Additional file 1: Fig S9A).

Further analysis of direct targets of TFs were compared (Fig. 8B and Table 2), with ChIP-seq analysis revealing overlap in four target genes whose expression was regulated by all the three TFs (Fig. 8C). A total of 32 gene targets that were identified as regulated by both BbOsrR1 and BbOsrR2 (ChIP-seq) indicated that the binding sites for the two TFs also partially overlapped in the promoters of 27 of these genes, with RNA-seq data showing transcription levels of 23 of these genes significantly altered in the Δ*BbOsrR3* mutant. BbOsrR3 binding motifs were found in promoter regions of 13 genes of those 23 genes, and these sequences partially overlapped with the BbOsrR1 and BbOsrR2 (ChIP-seq peaks). The two target genes encoded peroxisomal membrane protein 24 (BBA_00585) and CatA catalase (BBA_06186) (Fig. 8D). Of the 6 target genes shared by BbOsrR2 and BbOsrR3 (but not BbOsrR1), the ChIP-seq peaks for the two TFs showed partial overlap in the promoters of a subset of genes. With respect to the 83 targets only found in the BbOsrR1 ChIP-seq pool, expression of 23 genes were significantly altered in either the Δ*BbOsrR2* and /or Δ*BbOsrR3* strains. Based on MEME-MAST analysis, both BbOsrR2 and BbOsrR3 binding motifs were found in promoter regions of 3 of the 23 targets (despite no ChIP-seq signals). These targets were identified as a homocysteine S-methyltransferase (BBA_05409), heat-shock protein HSP98 (BBA_02283), and a hypothetical protein (BBA_05604) genes, in which their binding motifs partially overlapped with the BbOsrR1 ChIP-seq peak (Fig. 8E). Within the 389 targets only found in the BbOsrR2 ChIP-seq pool, expression of 121 targets were significantly altered in the Δ*BbOsrR1* and /or Δ*BbOsrR3* strains. Binding motifs for BbOsrR1 and BbOsrR3 were identified in the promoters of 2 genes (again, no ChIP-seq signals for these were identified), which included a hypothetical protein (BBA_03535) and glyoxalase domain-containing protein 5 (BBA_09002) genes. For these promoter sequences, potential BbOsrR1 and BbOsrR3 binding sites partially overlapped with the BbOsrR2 ChIP-seq peaks (Fig. 8F). With respect to the BbOsrR3 ChIP-seq pool, 32 unique targets were identified, in which 2 genes, encoding a fatty acid hydroxylase (BBA_02581) and a hypothetical protein (BBA_04703), were significantly altered in expression in the Δ*BbOsrR1* and Δ*BbOsrR2* mutant strains. However, binding motifs for BbOsrR1 and BbOsrR2 were only found in the fatty acid hydroxylase gene promoter, and partially overlapped with the BbOsrR3 ChIP-seq peak (Fig. 8G).

**Table 2:** BbOsrR1, BbOsrR2, and BbOsrR3 regulated genes

Promoter elements containing	Number of genes	Co-regulated genes
BbOsrR1, BbOsrR2, and BbOsrR3 binding sites	4	4
BbOsrR1 and BbOsrR2 binding sites only	32	13
BbOsrR2 and BbOsrR3 binding sites only	6	0
BbOsrR1 and BbOsrR3 binding sites only	4	0
BbOsrR1 binding sites only	83	3
BbOsrR2 binding sites only	389	2
BbOsrR3 binding sites only	32	1

### Identification of a BbClp1-BbOsrR2-BbOsrR3 complex and correlation analysis

**Fig. 9:**
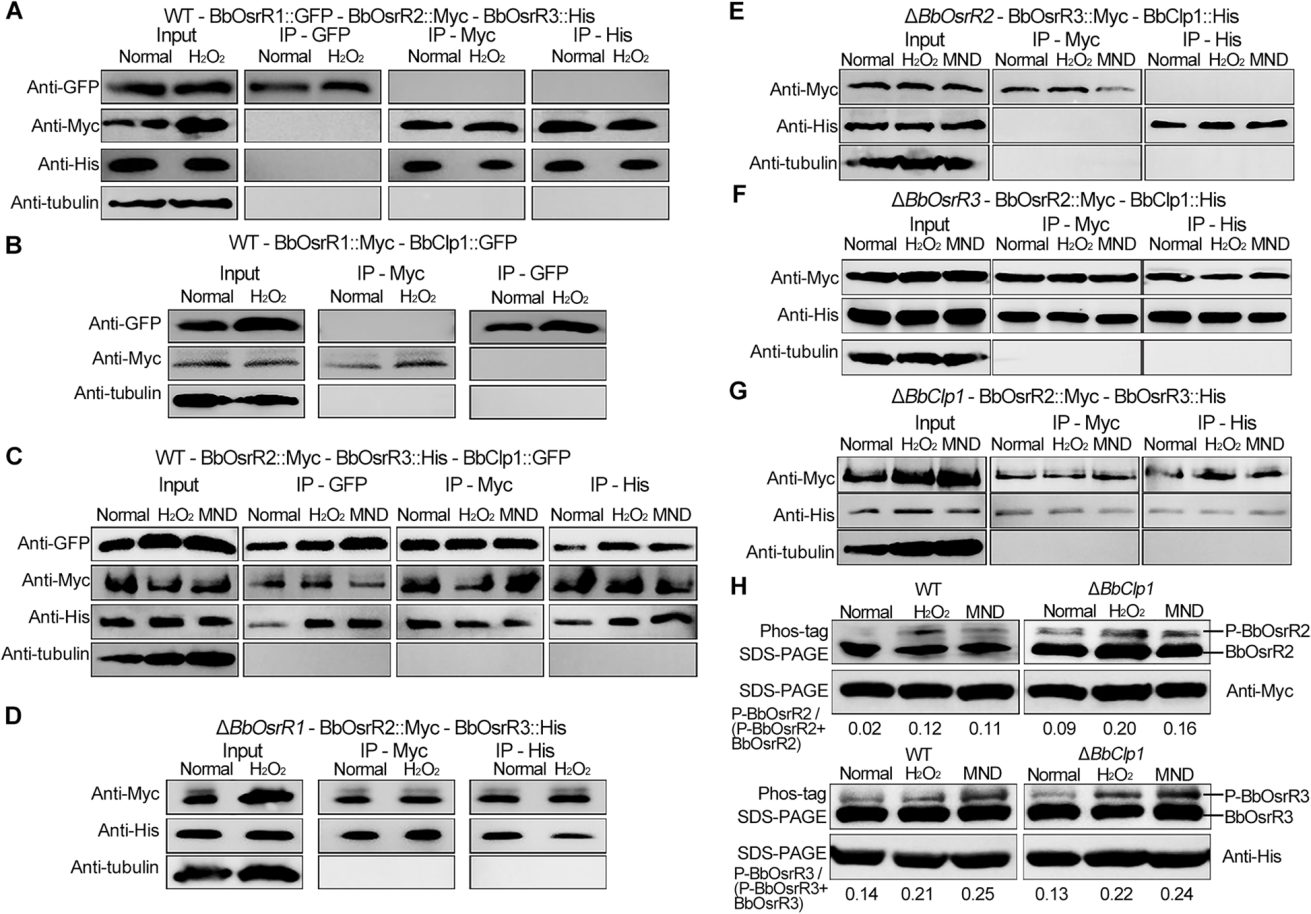
Immunoprecipitation (Co-IP) of a TF complex and phosphorylation of BbOsrR2 and BbOsrR3. **A** Co-IP assays using tagged versions of BbOsrR1, BbOsrR2, and BbOsrR3 as detailed in the “Methods” section. **B** Co-IP assays using tagged versions of BbOsrR1 and BbClp1. **C** Co-IP assays using tagged versions of BbOsrR2, BbOsrR3, and BbClp1. **D** Co-IP assays using tagged versions BbOsrR2 and BbOsrR3 in the Δ*BbOsrR1* strain. **E** Co-IP assays using tagged versions of BbOsrR3 and BbClp1 in the Δ*BbOsrR2* strain. **F** Co-IP assays using tagged versions of BbOsrR2 and BbClp1 in the Δ*BbOsrR3* strain. **G** Co-IP assays of tagged versions of BbOsrR2 and BbOsrR3 in the Δ*BbClp1* strain. Anti-β-tubulin antibody was used as an internal standard. **H** Assays for phosphorylation of BbOsrR2 and BbOsrR3 in the wild type and Δ*BbClp1* strains. Phosphorylation of BbOsrR2 and BbOsrR3 from fungal cultures was performed using the Myc-tagged version of BbOsrR2 and the His-tagged version of BbOsrR3 in cells grown under normal and oxidative stress for 30 min after Western blotting via Phos-tag™ SDS-PAGE as described in the “Methods” section. BbOsrR2 and BbOsrR3 proteins were blotted with their respective (anti-)tag antibodies. The relative amounts of the phosphorylated BbOsrR2 and BbOsrR3 as compared to their total protein levels for each sample were measured by densitometric analysis of bands using the ImageJ software

In order to identify interacting partners of the BbOsrR TFs, co-immunoprecipitation (Co-IP) experiments were performed using the Myc-tagged versions of the proteins. Western blotting indicated that “pull-down” of BbOsrR1 did not result in Co-IP of either BbOsrR2 or BbOsrR3 in cells grown in ¼ SDY, ¼ SDY + H_2_O_2_, or ¼ SDY + MND. However, pull-down experiments using either BbOsrR2 or BbOsrR3 revealed Co-IP of BbOsrR3 and BbOsrR2, respectively (Fig. 9A). No Co-IP signal was detected between BbOsrR1 and BbClp1 (Fig. 9B); however, Co-IP between all three partners, BbOsrR2, BbOsrR3, and BbClp1, were detected, with stronger signals noted between BbOsrR2 and BbClp1 in fungal cells grown in ¼ SDY + H_2_O_2_ and ¼ SDY + MND (Fig. 9C). To further understand the interaction of BbOsrR2, BbOsrR3, and BbClp1, pull-down experiments were performed in one of the gene disruption mutants. As expected from the results above, loss of BbOsrR1 did not affect the Co-IP of BbOsrR2 and BbOsrR3 (Fig. 9D). However, no Co-IP signal between BbOsrR3 and BbClp1 was seen in the Δ*BbOsrR2* mutant (Fig. 9E), whereas Co-IP was seen between BbOsrR2 and BbClp1 in the Δ*BbOsrR3* mutant, and between BbOsrR2 and BbOsrR3 in the Δ*BbClp1* mutant (Fig. 9F and G). These interactions were also confirmed by yeast-two hybrid tests (Additional file 1: Fig S9B). These results suggest a BbClp1-BbOsrR2 interaction, and a BbOsrR2-BbOsrR3 interaction that allows for a complex of the three partners, BbClp1-BbOsrR2-BbOsrR3.

In yeast, cyclins bind and activate cyclin-dependent protein kinases (Cdk), which further regulate protein targets via phosphorylation. The Cdk activity is inhibited via binding of inhibitory subunits termed CKIs (Cdk Inhibitors), resulting in dephosphorylation of their targets [49, 50]. Annotation of BbClp1 indicated the presence of a cyclin domain, and analysis of BbOsrR2 and BbOsrR3 indicated the presence of (at least) one predicted phosphorylation sites for a Cdks, namely, S168 in BbOsrR2 and T317 in BbOsrR3. To examine whether these proteins were indeed phosphorylated and what contribution BbClp1 may have on this phosphorylation, the phosphorylation levels of BbOsrR2 and BbOsrR3 in the wild type and Δ*BbClp1* strains were examined using Western blotting followed by probing of the blots with Phos-tag™ reagent. Very low level of phosphorylated of BbOsrR2 was seen under normal growth conditions, with BbOsrR2-phosphorylation increasing when cells were stressed by H_2_O_2_ (4 mM) and MND (60 μM) for 30 min (Fig. 9H). Phosphorylation of BbOsrR2 was retained in Δ*BbClp1* mutant cells, with higher basal and induced levels after exposure to the oxidative agents (Fig. 9H). Phosphorylation of BbOsrR3 was seen in the absence of stress, with levels increased in H_2_O_2_ and MND stressed cells, although no apparent changes were seen in Δ*BbClp1* cells as compared to the wild type strain (Fig. 9H).

To further reveal linkages between the BbOsrR TFs in the control of oxidative stress responses, correlation analysis was performed on the RNA-seq dataset derived from mutant strains of the three TFs, as well as Δ*BbClp1*. Heatmap analysis showed that BbOsrR1 and BbOsrR2-mediated genes displayed high correlation between the ¼ SDY and ¼ SDY + H_2_O_2_ datasets (correlation coefficient, R^2^ > 0.8). Lower correlation (R^2^ = 0.67) was seen between BbOsrR2 and BbOsrR3 RNA-seq comparing ¼ SDY datasets; however, the R^2^ values increased to 0.8 and 0.74 when comparing fungal cells stressed by H_2_O_2_ and MND, respectively. Similarly, very high correlations between the Δ*BbOsrR3* and Δ*BbClp1* datasets were seen under H_2_O_2_ and MND stress (R^2^ > 0.94), although an R^2^ = 0.74 was seen when comparing the ¼ SDY datasets. Strong correlations were also seen between Δ*BbOsrR1* (H_2_O_2_ stress) and Δ*BbOsrR2* and Δ*BbClp1* datasets (R^2^ > 0.74) (Additional file 1: Fig S9C).

## Discussion

Cellular responses to oxidative stress are essential for maintenance of cell integrity. In multicellular organisms, oxidative damage can lead to uncontrolled cell proliferation (cancer) and / or cell death and necrosis. For (eukaryotic) micro-organisms, resistance or mitigation of oxidative stress is required for viability, and, for pathogens, affects the ability to successfully infect hosts. Several partially redundant signaling pathways that include the Hog1 MAP kinase, the PKC1-MAP kinase and CWI pathways, and other factor such as the cytokinesis-required Cdc14 phosphatase and a P-type calcium ATPase, as well as several transcription factors (TFs), have been showed to regulate oxidative / ROS stress responses in various yeasts and filamentous fungi [15–37]. Here, we report an additional regulatory circuit mediated by three Zn_2_Cys_6_ TFs, BbOsrR1, 2, and 3, as well as a cyclin domain-containing protein, BbClp1, that orchestrates the regulation of fungal oxidative stress response-involved genes. These TFs were identified using the promoter element of one downstream target of the Hog1-MAP-kinase pathway, namely *BbOhmm*, which encodes a unique fungal mitochondrial membrane protein that negatively affects oxidative stress responses and virulence, including mediation of intracellular ROS levels in *B. bassiana* [2, 43]. BbOsrR1, 2, and 3 were identified via yeast-one hybrid screening and the binding of each protein as well as the identification of putative binding sequence motifs were verified using EMSA with purified proteins and various target sequences. ChIP-qPCR analysis revealed high occupancy of BbOsrR2 to the promoter region of *BbOhmm*, with lower enrichment of BbOsrR1 and BbOsrR3 under the conditions tested including cells grown under oxidative stress, with the *BbOhmm* promoter element present in the BbOsrR2 ChIP-seq pool but not in ChIP pools of the other TFs. RNA-seq analysis revealed that *BbOhmm* expression was significantly upregulated in the Δ*BbOsrR2* strain under oxidative stress conditions but repressed in Δ*BbOsrR3* strain. These data support a model in which BbOsrR2 acts as a (strong) negative regulator of *BbOhmm* expression in response to both H_2_O_2_ and menadione, whereas BbOsrR3 acts as a positive regulator of *BbOhmm* expression, with the role / functioning of BbOsrR1 with respect to *BbOhmm* expression unclear.

**Fig. 10:**
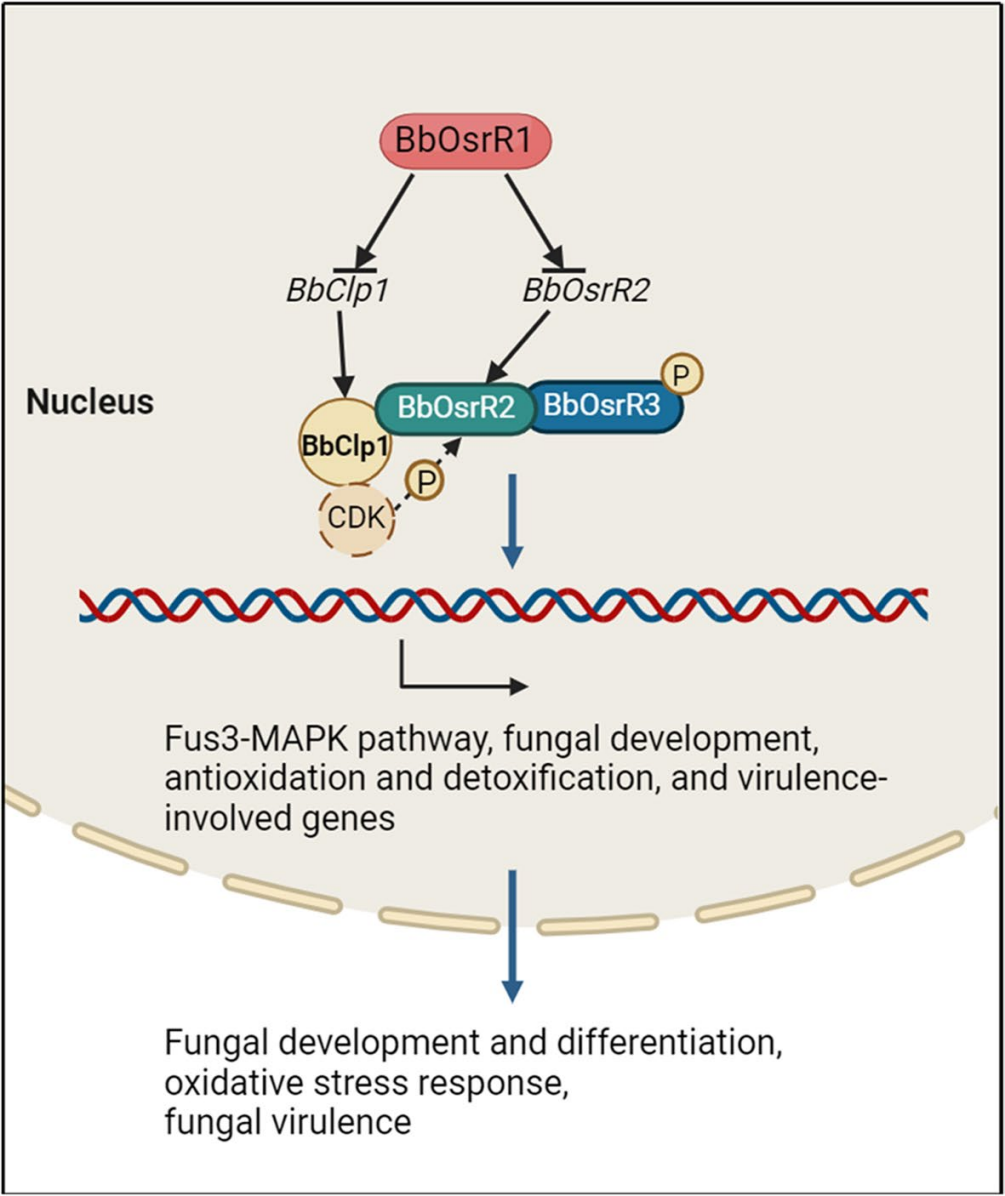
The network consisting of three TFs, BbOsrR1, 2, and 3, and cyclin BbClp1 orchestrates *B. bassiana* development, oxidative stress responses, and virulence. BbOsrR1 negatively regulates *BbOsrR2* and *BbClp1* transcription via targeting their promoters. BbClp1, BbOsrR2, and BbOsrR3 forms a regulatory complex, in which BbClp1 directly interacts with BbOsrR2 but not with BbOsrR3, with BbClp1 partially regulating BbOsrR2 phosphorylation. The network finely regulates fungal conidiation and germination, oxidative stress responses, and virulence via control of Fus3-MAPK pathway, fungal development, antioxidation and detoxification, and virulence-involved genes

In terms of effects on global gene expression networks, *BbOsrR1* and *BbOsrR3* mutation led to increased tolerances to free radical generating compounds (H_2_O_2_ or / and menadione), whereas the Δ*BbOsrR2* strain displayed decreased tolerances to these compounds, indicating that the former two TFs suppress genes involved in oxidative damage response, whereas the latter TF acts to induce oxidative stress genes. These results are supported by characterization of the DEG datasets in which expression of the oxidative damage response genes, encoding glutathione S-transferase, catalase (catA), oxidoreductase family protein, and LPG synthase protein, major facilitator superfamily transporter, metalloreductase, etc., were increased in the Δ*BbOsrR1* and Δ*BbOsrR3*, whereas transporter and detoxification genes, e.g., major facilitator superfamily transporter, chromate transporter, cytochrome P450 Cyp617A2, short-chain dehydrogenase genes, were decreased in the Δ*BbOsrR2* strain. ChIP-seq and RNA-seq revealed that BbOsrR1 directly targeted the promoter of *BbOsrR2* and a cyclin-like protein BbClp1 gene. BbClp1 was shown to localize to the nucleus and targeted gene disruption of *BbClp1* (Δ*BbClp1* mutation) negatively affected oxidative stress response, confirming its role as part of the OSR circuit, with expression of *BbOhmm* was significantly upregulated in the Δ*BbClp1* strain. Similar tolerances of mutant strains of the three TFs and BbClp1 to oxidative stresses were tested on the minimal medium CZA, and expression patterns of some key antioxidant / detoxification genes in those mutants in CZB were also similar to the ¼ SDY-derived RNA-seq data. The results suggested that the three TFs and BbClp1-mediated oxidative stress responses seemed independent of the effects of growth media. A series of Co-IP experiments revealed the following: (i) no clear partners for BbOsrR1, (ii) Co-IP between BbOsrR2, BbOsrR3, and BbClp1 under both normal and oxidative stress conditions. These experiments suggested that the three partners, BbOsrR2, BbOsrR3, and BbClp1 interacted and formed a complex. Co-IP experiments in the various deletion mutant backgrounds revealed that BbClp1 directly interacted with BbOsrR2 but not BbOsrR3 in the complex of the three partners, which was verified by yeast-two hybrid experiments. These experiments suggest a model in which BbOsrR3 and BbClp1 interact directly with BbOsrR2 but not each other within a complex containing the three proteins (Fig. 10). Data further confirming the functioning of these proteins as a complex is indicated by the high correlations seen between the DEG datasets of the transcriptomes of mutant strains of each of these genes under normal and oxidative stress (H_2_O_2_ and menadione) conditions.

While additional work is needed to determine the exact function of BbClp1 within the complex, cyclin and cyclin-dependent protein kinases (Cdks), as well as their associated regulators, are known to control cell division and other important functions in eukaryotic cells [51, 52]. Cyclins bind and activate a cyclin-dependent kinase (Cdk) subunit, with the activated Cdk targeting the phosphorylation of downstream effectors that can include TFs. Cdk activity is inhibited via cyclin kinase inhibitory subunits (Cdk Inhibitors, CDKIs), which allow for tuned regulatory cascades that can rapidly respond to various cell stimuli [49, 50]. *S. cerevisiae* contains 23 cyclins and 6 Cdks indicating shared elements between different pathways / complexes [53]. Thus far, however, to the best of our knowledge, a cyclin interacting (or forming a complex) with OSR TFs has yet to be reported. The *B. bassiana* genome encodes at least 14 cyclin-like proteins and 11 CDK-like proteins, whose functions remain largely uncharacterized. Although it is unclear how BbClp1 affects the BbClp1-BbOsrR2-BbOsrR3 complex, our data do indicate that both BbOsrR2 and BbOsrR3 are phosphorylated, with levels increasing in response to oxidative stress. However, only a slight increase in BbOsrR2 phosphorylation under non-stress condition was seen in the Δ*BbClp1* background. Given that BbOsrR2 acts as a negative regulator of *BbOhmm* expression in response to H_2_O_2_ and menadione, and BbOsrR3 acts as a positive regulator of *BbOhmm* expression, it is likely that the increased phosphorylation seen for both proteins after oxidative stress results in opposing effects in the activities of these TFs. Although no CDK or CDKI were identified in our Co-IP experiments, our data suggest their involvement, with further work, is needed to characterize if/how BbClp1 and any associated CDKs / CDKIs contribute to the BbOsrR2-BbOsrR3 circuit.

By combining the ChIP- and RNA-seq analysis as well as direct EMSA-mediated binding to putative promoter element sequences, the DNA-binding motifs for the three TFs were identified and found to aggregate with partial overlap in the promoter region of *BbOhmm* as well as other gene targets. These data suggest that for some promoters, the BbOsrR TFs may compete for binding to regulatory elements, thus providing for a mechanism for their differential binding and effects on the regulation of target genes. In addition, BbOsrR1 was found to directly bind (via EMSA) to the promoter regions of both BbOsrR2 and BbClp1, indicating a feedback loop that can coordinate the expression of this circuit. RNA-seq data showed that BbOsrR1 also targets several antioxidation and detoxification genes, which correlates to the increased H_2_O_2_ tolerance phenotype of the Δ*BbOsrR1* mutant, as the TF appears to act to repress factors that contribute to oxidative stress resistance.

Our data also show input of BbOsrR2 and BbOsrR3 into the Fus3/Kss1-MAP kinase pathways. One direct (repressor) target of BbOsrR2 is the *Msg5* encoding dual specificity phosphatase, which was significantly upregulated in the Δ*BbOsrR2* strain. Yeast Msg5 specifically dephosphorylates threonine and tyrosine residues in the CWI MAP kinase Mpk1 and Fus3/Kss1 MAPK pathways [45, 48]. Our RNA-seq data show that the Fus3-MAP kinase pathway components, *Ste4*, *Fus3*, and *Cdc28* were significantly upregulated in the Δ*BbOsrR2* strain. Despite this increase, however, one consequence of *Msg5* upregulation in the Δ*BbOsrR2* strain may be dampening of the Fus3/Kss1 MAP kinase signaling which would result in decreased virulence and reduced conidial production, phenotypes seen for the Δ*BbOsrR2* strain. Disruption of the Fus3/Kss1 MAP kinase gene, *Bbmpk1*, in *B. bassiana* also results in decreased virulence, but unlike the Δ*BbOsrR2* mutant, increased growth is also seen [54, 55]. Expression of *Cdc28*, involved in cell cycle progression, acting as downstream factor of the Fus3/Kss1 MAP and Hog1 MAP kinase pathways [47, 56, 57], was also significantly upregulated in the Δ*BbOsrR2* strain, potentially helping account for the conidiation and germination defects seen. In addition, the MADS-box TF, *Mcm1* gene was found to be a direct target of BbOsrR2 and significantly upregulated in the Δ*BbOsrR2* strain. In *S. cerevisiae*, Mcm1 controls minichromosome maintenance, general metabolism, cell integrity, and pheromone responses [58, 59], interacting with the Fus3 / Kss1 target TF, Ste12, to activate pheromone-inducible genes required for mating and cell fusion [58, 60]. The Fus3 / Kss1 MAP kinase pathway also controls fungal virulence via activation of Ste12 [61], and regulates cell cycle via cyclin-dependent serine/threonine-protein kinase Cdc28 complex [62], in which the latter controls fungal development and differentiation. In filamentous fungi, Mcm1 can also partner with Ste12 to regulate cell cycle progression and cell integrity, affecting fungal growth, development, and virulence [46]. Thus, upregulation of *Mcm1* in the Δ*BbOsrR2* strain might also contribute to the abnormal conidiation, germination, and virulence phenotypes seen, as well as to the increased oxidative stress sensitivities of the mutant strain. With respect to BbOsrR3, this TF was shown to directly target as a negative regulator the Fus3 / Kss1-MAPK gene, whose expression was significantly upregulated in the Δ*BbOsrR3* strain under oxidative stress conditions. RNA-seq data revealed that expression of the Fus3 / Kss1 MAP kinase pathway-involved genes, including the Ste2 G protein-coupled receptor, the (G-protein a subunit, Gpa1) negative regulator, Sst2 [47], and the Msg5 genes, were significantly upregulated in the Δ*BbOsrR3* strain. In this case, increased expression of these pathway components may contribute to the increased virulence phenotype seen for the Δ*BbOsrR3* strain. In addition, a PTH11-like integral membrane protein gene implicated in virulence was found in both the BbOsrR2 and BbOsrR3 ChIP-seq pools; however, expression of this gene was downregulated in the Δ*BbOsrR2* strain but upregulated in the Δ*BbOsrR3* strain. The PTH11-like shares identity with the MrGpr8 G protein-coupled receptor (34%) which has been shown to mediate appressorium formation by controlling the nuclear translocation of the Fus3-MAP kinase in the related insect fungal pathogen, *M. robertsii* [62]. These results support a competing regulatory circuit composed of BbOsrR2 and BbOsrR3 that fine tunes regulation of the Fus3-MAP kinase pathway, controlling oxidative stress responses, and contributing to fungal development, differentiation, and virulence (Fig. 10). Our ChIP-seq and RNA-seq data also demonstrated that BbOsrR2 and BbOsrR3 have discrete targets (in addition to common targets). How these are acted upon within the context of the BbOsrR3-BbOsrR2-BbClp1 complex, i.e., whether these TFs act separately as well as part of the complex, remains to be determined. Moreover, RT-qPCR analysis revealed that 1 to 3 fungal central development pathway (CDP) genes, *BrlA*, *AbaA*, *WetA*, or/and *VosA* were significantly increased or decreased at conidiation stage of Δ*BbOsrR1*, Δ*BbOsrR2*, Δ*BbOsrR2*, or Δ*BbClp1* strain, which were in line with their expression patterns in the RNA-seq data. Contribution of the altered expression patterns of those CDP genes to the abnormal conidiation, germination, and virulence of those mutants might be included, although the underlying regulatory mechanism is unclear and needs to be revealed in the future experiments.

## Conclusions

Three Zn_2_Cys_6_ transcription factors, BbOsrR1, BbOsrR2, and BbOsrR3, were identified as regulators of the oxidative stress response pathway as well as more broadly contributing to fungal development and virulence. A feedback loop where BbOsrR1 directly targets *BbOsrR2* and *BbClp1* expression was identified. In addition, a complex involving the BbClp1 cyclin, BbOsrR2, and BbOsrR3 was identified. These results expand the regulatory mechanisms employed by cells to respond to oxidative stress.

## Methods

### Bacterial and fungal strains

Bacterial strains of *Escherichia coli* DH5a, *E. coli* BL21, and *Agrobacterium tumefaciens* AGL-1 were used for DNA manipulations, protein expression, and fungal transformations, respectively. The yeast Y1HGold and Y2HGold strains (Clontech, USA) were used for the yeast-one hybrid experiments, transcriptional activation assays, and yeast-two hybrid tests, respectively. Maintenance and growth of strain were performed as described previously [63]. *Beauveria bassiana* wild type strain Bb0062 (CGMCC 7.34) and the *BbOhmm* disruption mutant (Δ*BbOhmm*) [2] were typically grown in potato dextrose agar /broth (PDA / PDB), Czapek-dox agar / broth (CZA / CZB), and/or ¼ strength Sabouraud dextrose media (¼ SDAY / ¼ SDY) amended as indicated.

### Molecular manipulation

All primers used for gene manipulation are given in Additional file 2: Table S1. Different lengths of the *BbOhmm* promoter region with the entire *BbOhmm* coding region were constructed by design of suitable primers and PCR amplification of resultant fragments using *B. bassiana* genomic DNA as the template. Promoter fragments with the coding region were cloned into the *Xba*I site of pK2sur [64]. The resultant vectors were then transformed into the Δ*BbOhmm* strain using *A. tumefaciens*-mediated fungal transformation [65]. Putative transformants were isolated on CZA plates supplemented with 40 μg / mL sulfonylurea and 200 μg / mL phosphinothricin, and the integrity of the construct subsequently verified by PCR with respective primers. Verified transformants were inoculated on CZA + 5.76 mM H_2_O_2_ to examine rescue of the sensitivity to oxidative stress phenotype of the Δ*BbOhmm* strain to wild type. Site directed mutations in the regulatory element were introduced via primer design, and the desired sequence was amplified and cloned using overlap PCR and subsequently introduced and screened in the Δ*BbOhmm* strain as above.

Target gene knockouts were constructed in *B. bassiana* using *A. tumefaciens-*mediated homologous recombination with the herbicide resistance *bar* gene as the selective marker as described previously [2]. Complementation strains were constructed by introduction of the wild type gene (entire coding region + 1.5–2 kb of upstream sequences) into each respective mutant strain using a vector containing the sulfonylurea resistance *sur* gene as the selection marker as described previously [2, 9].

Subcellular localization of indicated proteins was examined via construction of enhanced green fluorescence protein (eGFP) gene fused to the 3′-end of the target gene, followed by transformation and expression in wild type *B. bassiana*. Briefly, coding regions corresponding to *BbOsrR1* and *BbOsrR2* were amplified from wild type genomic DNA and separately inserted into the *Bam*HI / *Eco*RI sites of pBARGPE-GFP [66] resulting in a fusion of the *eGFP* to the 3′-end of the genes, with expression under control of the *gpdA* promoter. For *BbOsrR3* and *BbClp1*, 1.5–2 kb of upstream (promoter) were amplified and cloned into the *Nde*I / *Sma*I sites (for *BbOsrR3*) and *Nde*I/ *Eco*RV sites (for *BbClp1*) of pBARGPE-GFP resulting in *eGFP* fused to the 3′-end of the genes, with expression under control of the native promoter. All the resultant vectors were transformed into the *B. bassiana* wild type strain as above. eGFP fluorescence signals were examined using an inverted confocal laser scanning microscope (*E*_*x*_ = 488 nm, *E*_*m*_ = 507 nm, SP8, Leica) with an argon ion laser. A *B. bassiana* strain carrying pBARGPE1-GFP was used as the positive control (*eGFP* was under the control of *gpdA* promoter) [66]. Where indicated, the nucleus was counter stained with DAPI (4', 6-diamidino-2-phenylindole, *E*_*x*_ = 350 nm, *E*_*m*_ = 470 nm).

### Construction of yeast-one hybrid library and transcriptional activation assays

A three tandem repeat sequence corresponding to 5′-ATATC, found in the promoter region of *BbOhmm* (− 1082 to − 1088 bp from the start codon), was constructed and cloned into *Sac*I / *Xho*I sites of plasmid pAbAi (Clontech, USA) to form the bait vector (pAbAi-bait). The bait plasmid was introduced and screened in the yeast Y1HGold strain according to the manufacturer’s instructions. Positive clones were cultured on uracil-free synthetic dropout medium (SD) (Clontech) containing 0, 100, and 200 ng/mL aureobasidin A (AbA). Colonies capable of growing on AbA-contained agar were deemed as candidates for transcriptional activation. The bait vector alone showed no transcriptional activation (negative control).

To construct the screening library, *B. bassiana* was grown in ¼ SDY grown for 2 days (26 °C, with aeration), and then aliquots (20 mL) were added to ¼ SDY (final volume of 50 mL) containing 5.76 mM H_2_O_2_ and returned to the incubator with shaking for 5–120 min. Total RNA was isolated from cultures at 5, 10, 20, 60, and 120 min, after which the RNA was pooled for construction of the yeast-one hybrid library using the Matchamker™ One-Hybrid Library Construction kit (Clontech, USA) according to the manufacturer’s instructions. The constructed library and the linearized pGADT7-Rec were co-transformed into the Y1HGold strain containing the bait vector, pAbAi-bait. Transformants were cultured on 100 ng / mL AbA-contained leucine-free SD agar at 30 °C for 3 to 5 days. Positive colonies were verified using PCR with primer pair T7-F / T7-R (Additional file 2: Table S1). Clones containing fragments > 200 bp were sequenced. Predicted amino acid sequences from the clones were identified by searching the non-redundant protein sequence database using Blastx software (https://blast.ncbi.nlm.nih.gov/Blast.cgi). DNA-binding domains in proteins were analyzed using the conserved domains (CD) search in NCBI website.

For select positive clones, a second round of yeast-one hybrid screening was performed. Briefly, the DNA-binding domain-coding region of each protein was amplified by PCR using *B. bassiana* genomic DNA as the template and cloned into pGADT7 to produce pGADT7-BBA_04239, -BBA_01981, -BBA_01499, -BBA_06193, -BBA_00237, -BBA_05389, and -BBA_04821, respectively. The resultant plasmids were then transformed into the Y1HGold strain harboring pAbAi-bait. Transformants were cultured on leucine-free SD agar or leucine-free SD agar containing 125 ng/mL AbA to examine colony growth. Yeast strains harboring p53/AbAi + AD-p53 and p53/AbAi alone were used as positive and negative controls, respectively.

Transcriptional activation of each candidate protein was evaluated using a yeast-based assay. Briefly, the entire coding sequence of each gene (*BbOsrR1*, *BbOsrR2*, and *BbOsrR3*) was cloned into *Xma*I/ *Not*I sites of vector pGBKT7 (Clontech, USA) using *B. bassiana* cDNA as the template, with expression of the genes under the control of a GAL4 promoter. Constructs were then transformed into the yeast Y2HGold strain and screened as described previously [63], for survival on SD agar + 125 ng/mL AbA and tryprotphan-free SD agar + 0.5 mM X-α-gal. Negative and positive controls were made by transforming the yeast strain with the empty vector and with the pGBKT7 vector containing the GAL4 activation domain sequence (pGBKT7-53), respectively.

### Electrophoretic mobility shift assay (EMSA)

EMSA was used to examine the ability of each TF to bind promoter regions of *BbOhmm* and other target genes. Briefly, the predicted DNA-binding domains of BbOsrR1, BbOsrR2, and BbOsrR3 were amplified and inserted into the T7 promoter-based bacterial expression plasmid, pET-28a, creating histidine-tagged fusion products for each protein. Plasmids were introduced into *E. coli* BL21 with protein expression and purification following standard protocols. Briefly, after expression, proteins were purified using a Mag-Beads His-Tag protein purification kit (Sangon, China). Predicted promoter region for each target gene was amplified and used for EMSA assays. Detection of DNA in gels was performed using the LightShift™ Chemiluminescent EMSA Kit (Thermo, USA) following the manufacturer’s instructions.

To examine possible binding motifs of BbOsrR1, 2, and 3, all the “C” and “G” nucleotides in the following sequences of the *BbOhmm* promoter were mutated to “T” and “A,” respectively with the sequences covering the identified motif “ATATC” (20 bp) (Mutation A, at − 1091 to − 1071 bp upstream of the ATG translation start site). (1) The 5′- and 3′-*BbOhmm* promoter regions were separately amplified using primer pairs POhmm-F/ MutationAR and MutationAF / POhmm-R, in which the mutated sites were introduced. The resultant products were amplified with overlap PCR, forming the Mutation A-contained promoter sequence (524 bp). (2) For Mutation B: the sequences flanking the “Mutation A”, i.e., the 15 bp at 5′-end and 18 bp at 3′-end of Mutation A at − 1105 bp to − 1090 bp and − 1073 bp to − 1055 bp upstream of the coding region, were separately amplified with primer pairs POhmm-F / MutationBR and MutationBF / POhmm-R, introducing mutated sites in the primers, which were then amplified using overlap PCR. (3) For Mutation C: the sequences containing Mutation A and B (50 bp) were combined. The promoter regions were separately amplified using Mutation B-contained promoter sequence as template with primer pairs POhmm-F/ MutationCR and MutationCF / POhmm-R. The PCR products were then amplified using overlap PCR. EMSA tests were performed using the *E. coli*-expressed DNA-binding domain of BbOsrR1, 2, or 3 with the mutated sequence-contained promoter region (524 bp) of *BbOhmm*. For detection of binding of BbOsrR1, 2, and 3 to promoter regions of their other targets using EMSA tests, promoter region harboring mutated binding motif for each target was used as scrambled DNA sequence control, in which all the “C” and “G” in binding motif were mutated to the “T” and “A,” respectively. Briefly, the 5′- and 3′-end of promoter fragments of the BbOsrR1 target, *CatA*, were separately amplified using two primer pairs, PCatA-F / MuPcatAR and MuPcatAF/ PCatA-R, in which mutated sites were introduced. The resultant PCR products were then amplified using overlap PCR (537 bp). Promoter regions of other two BbOsrR1 targets, *BbOsrR2* (586 bp) and *BbClp1* (243 bp), were amplified using primer pairs, MuPOsrR2F/ POsrR2R, and MuPClp1F/ PClp1R, respectively, in which the mutated sites were introduced. The 5′- and 3′-end of promoter fragments of the BbOsrR2 targets, *Msg5*, *Mcm1*, and BBA_06338, were seperatly amplified using two primer pairs, PMsg5-F / MuPMsg5R and MuPMsg5F / PMsg5-R, PMcm1-F / MuPMcm1R and MuPMcm1-F / PMcm1-R, and P06338-F / MuP06338R and MuP06338F / P06338-R, in which the mutated sited were introduced. All the PCR fragments for each promoter region were fused using overlap PCR (277, 256, and 589 bp, respectively). The 5′- and 3′-end of promoter fragments of the BbOsrR3 target, *Fus3*, were amplified using two primer pairs, PFus3-F / MuPFus3R and MuPFus3F / PFus3-R, respectively, in which mutated sites were introduced. The resultant PCR products were then amplified using overlap PCR (284 bp). All the primers are shown in Additional file 2: Table S1.

### Phenotype assays and insect bioassays

Growth and sensitivity of fungal strains to oxidative stress were examined on ¼ SDAY or CZA and ¼ SDAY or CZA amended with 4 mM H_2_O_2_ or 60 μM menadione (MND) as described previously [2]. Briefly, 2 μL conidial suspension (1 × 10^7^ spores/ mL) were spotted in the center of plates and cultured at 26 °C. Radial growth was measured over a time course (up to 7 days). Experiments were repeated three times with at least two independent batches of conidia. Relative growth inhibition (RGI) was used to compare the colony growth rate under the tested stress condition against their corresponding controls under the normal conditions [63]. Colony growth, conidiation, and conidial germination on different media, CZA, PDA, and /or ¼ SDAY, were assayed as described previously [2]. Alternatively, sensitivity of conidia to oxidative stress was assayed on ¼ SDAY containing different concentrations of oxidant agents (0, 2, 4, 6, and 8 mM H_2_O_2_, or 0, 20, 40, 60, and 80 μM MND) by examination of conidial germination at 24 h after inoculation. The median inhibiting concentration (IC_50_) for each fungal strain was calculated using the probit regression model and the SPSS 17.0 program.

Insect bioassays were performed using the last-instar larvae of *Galleria mellonella* either by topically inoculation (natural route of infection) or by direct injection into the hemocoel (bypassing the cuticle) as described previously [9]. Briefly, larvae were inoculated by spray of 1 mL conidial suspension (1 × 10^7^ spores/ mL) using a Potter spray tower (Burkard Manufacturing Co Ltd., UK). Alternatively, 2 μL of conidial suspension (5 × 10^6^ spores/ mL) was micro-injected larvae via the second proleg using an infusion / withdrawal pump (Baoding Shenchen Precision Pump Co Ltd., China). Each treatment was performed with three replicates of 30 insects each, and the experiments were repeated twice. Survival data were plotted as Kaplan–Meyer curves, and a log-rank test was used to analyze differences between groups. The median lethal time (LT_50_) was calculated using SPSS 17.0 program.

### Chromatin immunoprecipitation (ChIP)

To perform ChIP, a master vector, pK2-sur-13Myc, was constructed by introduction of 13 copies of the Myc epitope (13 Myc). Briefly, full-length *13* × *Myc* and terminator* T*_*ADH1*_ sequences were amplified from pFA6a-13Myc-KanMX6 [67] with paired primers p13myc-F / p13Myc-R (Additional file 2: Table S1), and the resultant fragment was inserted into the *Xba*I / *Hin*dIII sites of pK2-sur [64] to form pK2-sur-13Myc. The *gpdA* promoter (constitutive promoter) and coding region of *BbOsrR1* (deletion of the stop codons) were separately amplified from pBARGPE1 and *B. bassiana* genomic DNA using primer pairs *gpd*A-F / *gpd*A-R and OsrR1-F / OsrR1-R (Additional file 2: Table S1), and was fused using overlap PCR and inserted into the *Xba*I sites of pK2-sur-13Myc to add the 13 Myc-tag to the 3′-end of the gene. The integrity and correct orientation of all constructs were confirmed by sequencing. To add the 13Myc-tag to BbOsrR2 and BbOsrR3, the *B. bassiana* constitutive promoter PB3 [9, 68] was used to control the expression of the fusion genes. Briefly, promoter PB3 and the coding region of *BbOsrR2* (without stop codons) were separately amplified with primer pairs PB3OsrR2-F / PB3OsrR2-R and OsrR2-F / OsrR2-R, and fused using overlap PCR. The resultant fragment was cloned into the *Xba*I / *Pac*I sites of pK2-sur-13Myc (tag added to the 3′- end of the gene) to yield pK2-sur-BbOsrR2::Myc. Similarly, the promotor PB3 and the coding region of *BbOsrR3* (without stop codons) were separately amplified using primer pairs PB3OsrR3-F / PB3OsrR3-R and mycOsrR3-F / mycOsrR3-R, respectively, fused using overlap PCR, and then cloned into *Xba*I / *Pac*I sites of pK2-sur-13Myc to form pK2-sur-OsrR3::Myc. All resultant vectors were individually introduced into corresponding *B. bassiana* gene disruption mutants using *Agrobacterium*-mediated transformation [65]. Transformants were verified by PCR analysis using primer pairs *gpd*A-F / OsrR1, PB3OsrR2-F / OsrR2-R, or PB3OsrR3-F / mycOsrR3-R and Western blotting with anti-Myc antibody (Thermo, USA) following selection using 40 μg / mL sulfonylurea on CZA.

ChIP was carried out as described previously [69] with 0.3 g mycelia (fresh weight) from ¼ SDY and ¼ SDY containing oxidants (4 mM H_2_O_2_ or 60 μM menadione) for 30 min with shaking aeration. The purified DNA from the immunocomplexes was dissolved in 100 μL 0.1 × TE buffer after volatilization of residual ethanol and used for ChIP-qPCR analysis and ChIP sequencing. The concentration of DNA was measured using a NanoPhotemeter (Implen, German).

ChIP-qPCR analysis was used to evaluate enrichment of each BbOsrR TF in *BbOhmm* promoter region using primer pair POhmm-F / POhmm-R (Additional file 2: Table S1) with the precipitated chromatin DNA / input DNA as template as described previously [70] using a Bio-Rad IQ5 thermocycler (Bio-Rad) and the SYBR Green Supermix (Takara, Japan). Relative enrichment of the ChIP DNA in the promoter region was normalized to the input DNA, which was calculated with formula: 2^ΔCT^ × 100%, in which ΔCT = CT_ChIP_-(CT_Iuput_-6.64) where CT_ChIP_ and CT_Iuput_ are the threshold cycle (CT) values for ChIP DNA and input DNA, respectively, as described previously [71].

ChIP libraries were sequenced using the Illumina HiSeq™ 2000 platform (Novogene, Bejing, China) for 76-bp paired-end sequencing. Prior to aligning to *B. bassiana* 2860 genome (accession: PRJNA38719) [30], reads were trimmed and cleaned of contaminating adaptors using trimmomatic software [72]. Peaks with a *P* value of < 0.001 were chosen as candidate binding sites and targeted genes were identified if peaks were located within their promoter regions (about 1.5 kb). The motifs within ChIP-seq peaks were analyzed using an online motif predictor, the Multiple Em for Motif Elicitation (MEME; https://meme-suite.org/meme/) tool [73]. The targeted genes were analyzed using Gene ontology (GO) term enrichment analysis (http://www.blast2go.com/b2ghome/) and Kytoto Encyclopedia of Genes and Genomes (KEGG) pathway enrichment analysis (http://www.genome.jp/kegg/genes.html).

### RNA sequencing (RNA-Seq)

Mycelia of the *B. bassiana* wild type strain and indicated gene knockout mutants were derived from cultures first grown in ¼ SDY for 3 days, after which aliquots (20 mL into final volume of 50 mL) were inoculated in ¼ SDY (normal) or ¼ SDY containing oxidants (4 mM H_2_O_2_ or 60 μM MND) for 30 min with shaking aeration and used for RNA isolation. Total RNA was extracted with a RNeasy plant miniKit (Qiagen Sciences). After removal of DNA contamination with RQ1 RNase-free DNase (Promega, USA), the RNA pools were sequenced (Novogene, Beijing, China) using the Illumina HiSeq™ 2000 platform. Reads were mapped to *B. bassiana* 2860 genome (accession: PRJNA38719) [30] after clearning of adaptor tags, low-quality tags, and tags with only a single copy. Differentially expressed genes (DEGs) were identified between the gene disruption mutant and the wild type strain RNA-seq libraries under the same cultural condition using the number of fragments per kb of exon region per million mappable reads (FPKM). A minimum of twofold expressional difference (i.e., log2 FoldChange <  − 1.0 or > 1.0) in the paired libraries was used as a standard to judge each DEG at the false discovery rate (FDR) of 0.05 or less [74]. DEGs were classified and annotated using gene ontology (GO) term and KEGG pathway enrichment analysis.

### Revers-transcription (RT)-PCR and RT-qPCR

RT-PCR and RT-qPCR were used for examination of gene expression analysis. Briefly, first-strand cDNA was prepared by reverse transcription of 2 μg of total RNA using an oligo (dT)-primed cDNA synthesis kit (Primer Script™ RT reagent Kit) and gDNA Eraser (TaKaRa, Dalian, China), which was used as the template for PCR amplification. RT-PCR analysis was performed for 25 cycles using *18S rRNA* (BBA_07911) as a reference gene. RT-qPCR was performed using a quantitative realtime PCR kit (Bio-Rad, Hercules, CA), in which *18S rRNA* was used as the reference gene for normalization of expression levels of the target genes [75]. RT-PCR analysis was used for examination of loss or gain of target gene transcription in the gene disruption mutant or its reverse complementation strains. Briefly, RNA was isolated from ¼ SDY for 48 h at 26 °C with shaking aeration (200 r.p.m) and used for RT-PCR analysis. RT-qPCR analysis was adopted to evaluate transcription patterns of *BbOsrR1*, *2*, and *3*, *BbClp1*, and *BbOhmm* in the wild type or/ and gene disruption mutant (Δ*BbOsrR1*, Δ*BbOsrR*2, and Δ*BbOsrR3*) strains under the normal (¼ SDY) and oxidative stress (4 mM H_2_O_2_ or 60 μM MND) conditions for 30 min. The key genes derived from ¼ SDY-based DEG datasets involved in antioxidant / detoxification were verified and evaluated in the CZB-cultured wild type and mutant strains either under no-stress and H_2_O_2_ (4 mM)- or menadione (60 μM)-stress conditions for 30 min using RT-qPCR. Transcription patterns of fungal central development pathway (CDP) genes, *BrlA*, *AbaA*, *WetA*, and *VosA*, were evaluated using RT-qPCR at conidiation stage (5 days on ¼ SDAY). All the primers for RT-PCR and RT-qPCR are shown in Additional file 2: Table S1.

### Co-immunoprecipitation (Co-IP) and yeast-two hybrid tests

Co-IP was performed to examine the interactions between BbOsrR1, BbOsrR2, BbOsrR3, and BbClp1. Briefly, the *B. bassiana* promotor PB3 [9] and the coding region of *BbOsrR3* (without stop codons) were separately amplified using primer pairs PB3OsrR3-F / PB3OsrR3-R and HisOsrR3-F / HisOsrR3-R, in which a 6 × *His* tag was introduced into the primers to result in a fusion of the tag to the 3′-terminus of *BbOsrR3*. The PCR products were fused using overlap PCR and inserted into *Bam*HI site of pK2-sur-BbOsrR2::Myc to form vector pK2-sur-BbOsrR2::Myc-BbOsrR3::His. The resultant vector was separately transformed into *B. bassiana* strain harboring the *BbClp1::GFP* fusion gene, the strain containing the *BbOsrR1::GFP* fusion gene, as well as into the Δ*BbOsrR1* or Δ*BbClp1* strain using *Agrobacterium*-mediated transformation [65]. Integrities of the sequences of all vectors were confirmed by sequencing. To detect the interaction between BbOsrR1 and BbClp1, pK2-sur-BbOsrR1::13 Myc (constructed in ChIP in this study) was introduced into *B. bassiana* strain harboring the *BbClp1::GFP* fusion gene. To probe effect of *BbOsrR3* disruption on the interaction BbOsrR2 with BbClp1, the promotor PB3 and coding region of BbClp1 (without stop codons) were separately amplified using primer pairs Clp1pB32F/ Clp1pB32R and HisClp12F / HisClp12R, in which a 6 × *His* tag was introduced into the primers to result in a fusion of the tag to the 3′-terminus of *BbClp1*. The PCR products were fused using overlap PCR and inserted into *Bam*HI site of pK2-sur-BbOsrR2::Myc to form vector pK2-sur-BbOsrR2::Myc-BbClp1::His. The resultant vector was introduced into the Δ*BbOsrR3* strain. To examine the effect of *BbOsrR2* mutation on the interaction of BbOsrR3 and BbClp1, the promotor PB3 and coding region of BbClp1 (without stop codons) were separately amplified using primer pairs Clp1pB33F/ Clp1pB33R and HisClp13F / HisClp13R, in which a 6 × *His* tag was introduced into the primers to result in a fusion of the tag to the 3′-terminus of *BbClp1*. The PCR products were fused using overlap PCR and inserted into *Bgl*II site of pK2-sur-BbOsrR3::Myc to form vector pK2-sur-BbOsrR3::Myc-BbClp1::His. The resultant vector was transformed into the Δ*BbOsrR2* strain. All the transformants were selected on CZA containing 40 μg / mL sulfonylurea or / and 200 μg / mL phosphinothricin. Protein expression was verified by Western blotting using commercial antibodies against the corresponding tags. All the used primers are shown in Additional file 2: Table S1.

Mycelia of transformants harvested from ¼ SDY at 26 °C for 3 days were inoculated into fresh ¼ SDY or ¼ SDY containing oxidants (4 mM H_2_O_2_ or 60 μM MND) and cultured for 30 min with shaking aeration. Cultures were collected and used for protein extraction. Briefly, 0.3 g mycelia (fresh weight) fungal cells were homogenized using a Tissue Cell destroyer (DS1000, New ZongKe Vial Disease Control BioTech LTD., Wuhan, China) at 2400* g* by addition in 1 mL cell lysis buffer for Western blotting and IP (Beyotime, China). The supernatant was used for Co-IP following centrifugation at 13,800* g* and 4 °C for 10 min. The sample was divided into four equal parts, in which the three parts were incubated at 4 °C for overnight separately by addition of 25 μL Anti-c-Myc Magnetic Beads (Thermo, USA), Anti-GFP Magnetic Beads (Thermo, USA) and HisPur Ni–NTA Magnetic Beads (Thermo, USA), and another part was used as input. The beads were collected after centrifugation at 13,800* g* for 2 min and washed with 0.1 M glycine buffer (pH 2.0) at room temperature for 15 min with gentle rotation. As for HisPur Ni–NTA Magnetic Beads, 500 mM imidazole were added in the wash buffer. The immunocomplexes and the input were used for Western blotting using anti-GFP, anti-Myc, and anti-His antibodies (Thermo, USA). Anti-β-tubulin antibody (Sigma-Alorich, USA) was used as an internal standard. Western blotting analysis was performed using corresponding antibodies and Clarity Max Western ECL Substrate (Bio-Rad, Philadelphia, PA) according to the manufacturer’s instructions.

Yeast-two hybrid tests were performed to examine the interactions between BbOsrR2, BbOsrR3, and BbClp1. Briefly, *BbClp1*, *BbOsrR2*, and *BbOsrR3* cDNA were separately amplified with primer pairs, BaitBbClp1F / BaitBbClp1R, LureBbOsrR2F / LureBbOsrR2R, and LureBbOsrR3F / LureBbOsrR3R. The resultant products were separately inserted into *Eco*RI / *Bam*HI sites of plasmid pGBKT7 (for *BbClp1*) and pGADT7 (for *BbOsrR2* and *BbOsrR3*) to form pGBKT7-BbClp1, pGADT7-BbOsrR2, and pGADT7-BbOsrR3*.* pGBKT7-BbClp1 and pGADT7-BbOsrR2 or pGADT7-BbOsrR3 were separately co-transformed into Y2HGold cell according to the manufacturer’s instructions, termed AD-BbOsrR2 BD-BbClp1 and AD-BbOsrR3 BD-BbClp1 strains. Positive transformants were grown on the medium (SD-His-Ade-Leu-Trp) with 0.5 mM X-α-gal and 350 ng / mL AbA. The Y2HGold cells harboring pGADT7-T and pGBKT7-53, or pGADT7-T and pGBKT7-Lam were used for positive or negative controls.

### Phosphorylation assays and correlation analysis between transcriptomic data

To examine the effect of *BbClp1* mutation on phosphorylation of BbOsrR2 and BbOsrR3, proteins were extracted using cell lysis buffer (Roche) from the wild type strain harboring BbOsrR2::Myc-BbOsrR3::His-BbClp::GFP and Δ*BbClp1* strain vectoring BbOsrR2::Myc-BbOsrR3::His (constructed for Co-IP) cultures in ¼ SDY and ¼ SDY containing oxidants (4 mM H_2_O_2_ or 60 μM MND) for 30 min with shaking aeration. Protein was quantified using the BCA assay kit (GENEray) and 100 μg proteins were separated by Phos-tag™ sodium dodecyl sulfate–polyacrylamide gel electrophoresis (SDS-PAGE) (containing 5.0 mM Phos-tag™ (Wako Pure Chemical Industries) and 10 mM manganese chloride (MnCl_2_)). The phosphorylated BbOsrR2 and BbOsrR3 were probed after Western blotting with anti-Myc and anti-His antibodies (Thermo, USA), respectively, and horseradish peroxidase (HRP)-conjugated goat anti-rabbit secondary antibody (Boster Biological Technology) as described previously [76, 77]. BbOsrR2 and BbOsrR3 proteins were probed with anti-Myc and anti-His antibodies following separation in SDS-PAGE, respectively. The relative amount of the phosphorylated BbOsrR2 and BbOsrR3 as compared to their total proteins for each sample was measured by densitometric analysis of bands using the ImageJ software [78].

To evaluate correlation of the three TFs and BbClp1-mediated fungal growth and development and oxidative stress response, transcriptomic data controlled by those factors were compared using an intergroup correlation analysis (ICA) with the OmicShare ICA tools2 (https://www.omicshare.com/tools). The Pearson correlation coefficient was calculated.  

### Data analysis

The differences between two groups and between more than two groups were tested using Student’s *t* test (*t*-test) and one-way analysis of variance (ANOVAs) with subsequent LSD test, respectively. *P* values less than 0.05 were considered statistically significant. All statistical analyses were carried out with the SPSS 17.0 program. All data shown present the results obtained from triplicated independent experiments with standard deviations.

### Supplementary Information

**Additional file1:** **Fig S1. **Sensitivities of *BbOhmm* reverse complement strains to oxidative stress. The reverse complement strains were generated by introduction of *BbOhmm *with different length of promotor sequences into Δ*BbOhmm*. P + number indicate length of promotor sequence. “P1500+ mutated sites” indicate the element ‘ATATC’ in 1500 bp promotor sequences of *BbOhmm* was mutated to CCCTC. Two μL conidial suspensions (1×10^7^ cells/ mL^−^^1^) were spot-inoculated onto CZM plates supplemented with H_2_O_2_ (5.76 mM) and incubated at 26℃ for 7 days. **Fig S2.** Identification of transcription factors of *BbOhmm* in response to oxidative stress. (A) DNA-binding domain-contained clones that were screened from the yeast-one hybrid library. (B) Verification of transcription control of *BbOhmm* by the screened DNA-binding domain-contained proteins in yeast. DNA-binding domain-coding region of the protein was cloned into pGADT7-Rec, which was introduced into the Y1HGold strain harboring pAbAi-bait (vectoring tandem repeat of three copies of the ‘ATATC’ element in *BbOhmm *promoter). The transformants were cultured on Leu-free SD agar or Leu-free SD agar containing 125 ng / mL Aureobasidin A (AbA) to examine colony growth. The yeast cells transformed with vector p53-AbAi and pGADT7-Recp53, or with the pGADT7 AD vector containing the DNA-binding sequence of candidate proteins and a blank vector (p53-AbAi) vector, were used as positive or negative controls, respectively. (C) Transcriptional activation assays in yeast. Yeast strain Y2HGold carrying fusion cassettes of the GAL4 DNA-binding domain (BDGal4; negative control), the GAL4 DNA-binding and activation domains (BDGal4-ADGal4; positive control), or the GAL4 DNA-binding and the indicated TF (BDGal4::TF) were cultured on Trp-free SD plates containing 0.5 mM X-α-gal and 125 ng/ mL AbA at 30^o^C for 3 days. (D) Domain organization of three TFs, BbOsrR1, BbOsrR2 and BbOsrR3. (E) RT-qPCR analysis of *BbOsrR1*, *BbOsrR2*, *BbOsrR3* and *BbClp1* expression in wild type strain under unstressed and H_2_O_2_ (4 mM) or menadione (MND, 60 μM)-stressed conditions for 30 min. The asterisks (*) and (**) in the column charts denote *P* < 0.05 and *P* < 0.01 for the stressed conditions versus the unstressed condition (¼ SDY) (t-test), respectively. **Fig S3.** Localization examination of the three TFs and BbClp1 in* B. bassiana*. GFP signals of BbOsrR1::eGFP, BbOsrR2::eGFP, BbOsrR3::eGFP and BbClp1::eGFP are distributed in nuclei of conidia, germlings without or with exposure to oxidative stress (4 mM H_2_O_2_ or 60 μM menadione for 30 min), and *in vivo* blastospores (derived from infected insect), which was stained with florescent DAPI (4', 6-diamidino-2-phenylindole). Scale bar = 10 μm. **Fig S4.** Molecular manipulations of the targeted gene disruption and complementation. (A) Target gene locus and gene replacement vector pΔ*BbOsrR1*/ *BbOsrR2*/ *BbOsrR3*/ *BbClp1*. The gene replacement vector contains *bar* cassette flanked by 5′- and 3′-border sequences of target gene. Homologous recombination (cross over event marked by “X”) resulting in a region of target gene was replaced by the *bar *cassette. Arrow indicates the orientation of transcription in the ORF. (B) Target gene complementation vector, pCB-*BbOsrR1*/ *BbOsrR2*/ *BbOsrR3*/ *BbClp1*, containing the sulfonylurea resistance marker (*sur*). (C) PCR analysis of wild type strain (WT), Δ*BbOsrR1*/ Δ*BbOsrR2*/ Δ*BbOsrR3*/ Δ*BbClp1*, and complemented mutant (*BbOsrR1*^*RC*^/ *BbOsrR2 *^*RC*^/ *BbOsrR3 *^*RC*^/ *BbClp1*^*RC*^). Desired integration events were confirmed by PCR using primers S-F and S-R. (D) RT-PCR confirmation of loss of gene expression in the gene disruption mutant and recovery in the complemented strain (Comp). Total RNA was isolated as described in the Methods section. Expression of gene was examined using *18S rRNA *as the reference gene in wild type (WT), gene disruption mutant, and reverse complemented mutant strains. **Fig S5.** Colony growth of wild type (WT), target gene disruption mutant and complemented strains. (A) Colony grown on Czapek-Dox agar (CZA), PDA and ¼ SDAY at 26^o^C for 7 days. (B) Growth rate of the colony. Colony diameter was measured daily after 3 days of inoculation using cross method with a vernier caliper. Colony growth rate was evaluated using linear-regression analysis. Error bars indicate SDs. (C) Conidial yield of the fungal strains on CZA and ¼ SDAY plates after 14 days at 26°C. (D) Conidial germination rate. Conidial germination was monitored over the indicated time course via microscopic analysis of samples. Conidia were considered germinated when the germ tube was equal in length to the diameter of the conidia. At least 300 conidia were examined and the experiment repeated using three independent batches of conidia. The time required to achieve 50% germination of conidia (GT_50_) was estimated by modeling analysis of the germination trend of each strain over the time of incubation. (E) RT-qPCR analysis of *BrlA*, *AbaA*, *WetA* and *VosA* at conidiation stage (5 days on ¼ SDAY). The average values with standard deviation (± SD) of triplicated experiments are shown in (C, D and E). The asterisks (*) and (**) in (C) and D) denote *P* < 0.05 and *P* < 0.01 for the indicated fungal strains versus the wild type strain (WT) (t-test), respectively. **Fig S6.** Sensitivities of the wild type and mutant strains to H_2_O_2_ and menadione (MND) on CZA and expression patterns of key antioxidant/ detoxification genes. (A) Vegetative growth of the wild type and mutant strains on CZA and CZA containing 4 mM H_2_O_2_ or 60 μM MND for 7 days. (B) Calculated relative growth inhibition (RGI) of* B. bassiana *isolates challenged with oxidative stressors for 7 days as described in Methods section. (C) RT-qPCR analysis of key antioxidant/ detoxification genes from RNA-seq datasets in the CZB-cultured mutants either under no-stress and H_2_O_2_ (4 mM)- or menadione (60 μM)-stress conditions for 30 min as compared to wild type strain. The asterisks (**) in the column charts denote *P* < 0.01 for the indicated fungal strains versus the WT (t-test). **Fig S7. **GO enrichment analysis of Δ*BbOsrR1*, Δ*BbOsrR2* or Δ*BbOsrR3* versus WT DEGs under normal condition (¼ SDY), specially induced by H_2_O_2_ (4 mM) or/and menadione (MND, 60 μM) stresses for 30 min, respectively, and those DEGs commonly induced by H_2_O_2_ and MND. **Fig S8.** Insect bioassays of the wild type (WT), *BbClp1 *disruption mutant and its’ reverse complementation strains. (A) Survival of *G. mellonella* larvae following topical infection with the spore suspensions (5 × 10^6^ conidia/ mL suspensions). (B) Survival of the larvae following injection into the second proleg with 2 μL of 1 × 10^6^ conidia/ mL suspensions. Control insects were treated with sterile water. The survival data were plotted as Kaplan–Meyer curves and difference of gene disruption strains from the WT and its reverse was analyzed using a log-rank test. The mean lethal time to kill 50% of targets (LT_50_) was estimated using the SPSS 17.0 program. The experiments were repeated twice. **Fig S9.** Scanning of binding motifs BbOsrR1, 2, in and 3 the three-copy tandem repeat of the ‘ATATC’ used for yeast-one hybrid screening (A), yeast two-hybrid tests of interactions between BbOsrR2, BbOsrR3 and BbClp1 (B), and correlation of three TFs and BbClp1-mediated genes under the normal or oxidative stress conditions (C). Gold cells co-transformed with the pGBKT7-BbClp1 and pGADT7-BbOsrR2/BbOsrR3 grown on the medium (SD-His-Ade-Leu-Trp) with 0.5 mM X-α-gal and 350 ng/ mL AbA for 3 d. The yeast cells transformed with vector p53-AbAi and pGADT7-Rec-p53 vector (Clontech) or with a blank vector p53-AbAi only were used as positive or negative controls, respectively. Transcriptomic data controlled by those factors were comparatively analyzed using an intergroup correlation analysis (ICA) with the OmicShare ICA tools2 (https://www.omicshare.com/tools). Pearson correlation coefficient was calculated and indicated.

**Additional file2:** **Table S1. **Primers used in this study. (xlsx). **Table S2. **DEGs of Δ*BbOsrR1* versus WT under normal condition (¼ SDY). (xlsx). **Table S3. **DEGs of Δ*BbOsrR1* versus WT under H_2_O_2_ (4 mM)-stress condition for 30 min. (xlsx). **Table S4. **DEGs of Δ*BbClp1* versus WT under normal condition (¼ SDY). (xlsx). **Table S5. **DEGs of Δ*BbClp1* versus WT under H_2_O_2_ (4 mM)-stress condition for 30 min. (xlsx). **Table S6. **DEGs of Δ*BbClp1* versus WT under menadione (60 μM)-stress condition for 30 min. (xlsx). **Table S7.** DEGs of Δ*BbOsrR2* versus WT under normal condition (¼ SDY). (xlsx). **Table S8. **DEGs of Δ*BbOsrR2* versus WT under H_2_O_2_ (4 mM)-stress condition for 30 min. (xlsx). **Table S9. **DEGs of ΔBbOsrR2 versus WT under menadione (60 μM)-stress condition for 30 min. (xlsx). **Table S10.** DEGs of Δ*BbOsrR3* versus WT under normal condition (¼ SDY). (xlsx). **Table S11**. DEGs of Δ*BbOsrR3* versus WT under H_2_O_2_ (4 mM)-stress condition for 30 min. (xlsx). **Table S12. **DEGs of Δ*BbOsrR3* versus WT under menadione (60 μM)-stress condition for 30 min. (xlsx). **Table S13. **BbOsrR1 target genes identified by ChIP-seq under normal condition. (xlsx). **Table S14. **BbOsrR1 target genes identified by ChIP-seq under H_2_O_2_ (4 mM)-stress condition for 30 min. (xlsx). **Table S15. **BbOsrR2 target genes identified by ChIP-seq under normal condition.. **Table S16. **BbOsrR2 target genes identified by ChIP-seq under H_2_O_2_ (4 mM)-stress condition for 30 min. (xlsx). **Table S17. **BbOsrR2 target genes identified by ChIP-seq under menadione (60 μM)-stress condition for 30 min. (xlsx). **Table S18. **BbOsrR3 target genes identified by ChIP-seq under normal condition. (xlsx). **Table S19. **BbOsrR3 target genes identified by ChIP-seq under H_2_O_2_ (4 mM)-stress condition for 30 min. (xlsx). **Table S20. **BbOsrR3 target genes identified by ChIP-seq under menadione (60 μM)-stress condition for 30 min. (xlsx)

**Additional file3:** Original images of EMSA, Western blot and PCR gel.

## Data Availability

All data generated or analyzed during this study are included in this article, its supplementary information files, and publicly available repositories. Sequencing data have been deposited in the NCBI SRA as Bioprojects and the accession codes are PRJNA868476 for RNA sequences [79] and PRJNA883178 for ChIP sequences [80]

## Abbreviations

AbA: Aureobasidin A
ChIP: Chromatin immunoprecipitation
Co-IP: Co-immunoprecipitation
Comp: Complemented strain
DAPI: 4', 6-Diamidino-2-phenylindole
DEG: Differentially expressed gene
H_2_O_2_: Hydrogen peroxide
EMSA: Electrophoretic mobility shift assays
GO: Gene Ontology
KEGG: Kyoto Encyclopedia of Genes and Genomes
MND: Menadione
NO: Nitric oxide
RGI: Relative growth inhibition
ROS: Reactive oxygen species
TF: Transcription factor
WT: Wild type

## References

[1] Graves DB (2012). The emerging role of reactive oxygen and nitrogen species in redox biology and some implications for plasma applications to medicine and biology. J Phys D Appl Phys.

[2] He Z, Zhang S, Keyhani NO, Song Y, Huang S, Pei Y, Zhang Y (2015). A novel mitochondrial membrane protein, Ohmm, limits fungal oxidative stress resistance and virulence in the insect fungal pathogen *Beauveria bassiana*. Environ Microbiol.

[3] Olsen LF, Issinger OG, Guerra B (2013). The yin and yang of redox regulation. Redox Rep.

[4] Aguirre J, Rios-Momberg M, Hewitt D, Hansberg W (2005). Reactive oxygen species and development in microbial eukaryotes. Trends Microbiol.

[5] Andreyev AY, Kushnareva YE, Starkov AA (2005). Mitochondrial metabolism of reactive oxygen species. Biochem Mosc.

[6] Thorpe GW, Fong CS, Alic N, Higgins VJ, Dawes IW (2004). Cells have distinct mechanisms to maintain protection against different reactive oxygen species: Oxidative-stress-response genes. Proc Natl Acad Sci USA.

[7] Temple MD, Perrone GG, Dawes IW (2005). Complex cellular responses to reactive oxygen species. Trends Cell Biol.

[8] Wang YL, Ruby EG (2011). The roles of NO in microbial symbioses. Cell Microbiol.

[9] Lu Z, Deng J, Wang H, Zhao X, Luo Z, Yu C, Zhang Y (2021). Multifunctional role of a fungal pathogen-secreted laccase 2 in evasion of insect immune defense. Environ Microbiol.

[10] Cessna SG, Sears VE, Dickman MB, Low PS (2000). Oxalic acid, a pathogenicity factor for *Sclerotinia sclerotiorum*, suppresses the oxidative burst of the host plant. Plant Cell.

[11] Langfelder K, Streibel M, Jahn B, Haase G, Brakhage AA (2003). Biosynthesis of fungal melanins and their importance for human pathogenic fungi. Fungal Genet Biol.

[12] Heller J, Tudzynski P (2011). Reactive oxygen species in phytopathogenic fungi: signaling, development, and disease. Annu Rev Phytopathol.

[13] Finkel T (2003). Oxidant signals and oxidative stress. Curr Opin Cell Biol.

[14] Lambeth JD (2004). NOX enzymes and the biology of reactive oxygen. Nat Rev Immunol.

[15] Ikner A, Shiozaki K (2005). Yeast signaling pathways in the oxidative stress response. Mutat Res.

[16] Vilella F, Herrero E, Torres J, de la Torre-Ruiz MA (2005). Pkc1 and the upstream elements of the cell integrity pathway in *Saccharomyces cerevisiae*, Rom2 and Mtl1, are required for cellular responses to oxidative stress. J Biol Chem.

[17] Gerik KJ, Bhimireddy SR, Ryerse JS, Specht CA, Lodge JK (2008). PKC1 is essential for protection against both oxidative and nitrosative stresses, cell integrity, and normal manifestation of virulence factors in the pathogenic fungus *Cryptococcus neoformans*. Eukaryot Cell.

[18] de Dios CH, Román E, Monge RA, Pla J (2010). The role of MAPK signal transduction pathways in the response to oxidative stress in the fungal pathogen *Candida albicans*: implications in virulence. Curr Protein Pept Sci.

[19] Wang J, Liu J, Hu Y, Ying SH, Feng MG (2013). Cytokinesis-required Cdc14 is a signaling hub of asexual development and multi-stress tolerance in *Beauveria bassiana*. Sci Rep.

[20] Wang J, Zhou G, Ying SH, Feng MG (2013). P-type calcium ATPase functions as a core regulator of *Beauveria bassiana* growth, conidiation and responses to multiple stressful stimuli through cross-talk with signaling networks. Environ Microbiol.

[21] Moye-Rowley WS (2002). Transcription factors regulating the response to oxidative stress in yeast. Antioxid Redox Signal.

[22] Alonso-Monge R, Román E, Arana DM, Prieto D, Urrialde V, Nombela C, Pla J (2010). The Sko1 protein represses the yeast-to-hypha transition and regulates the oxidative stress response in *Candida albicans*. Fungal Genet Biol.

[23] Ohmiya R, Kato C, Yamada H, Aiba H, Mizuno T (1999). A fission yeast gene (*prr1+*) that encodes a response regulator implicated in oxidative stress response. J Biochem (Tokyo).

[24] Lessing F, Kniemeyer O, Wozniok I, Loeffler J, Kurzai O, Haertl A, Brakhage AA (2007). The *Aspergillus fumigatus* transcriptional regulator AfYap1 represents the major regulator for defense against reactive oxygen intermediates but is dispensable for pathogenicity in an intranasal mouse infection model. Eukaryot Cell.

[25] Molina L, Kahmann R (2007). An *Ustilago maydis* gene involved in H_2_O_2_ detoxification is required for virulence. Plant Cell.

[26] Guo M, Chen Y, Du Y, Dong Y, Guo W, Zhai S, Zhang H, Dong S, Zhang Z, Wang Y, Wang P, Zheng X (2011). The bZIP transcription factor MoAP1 mediates the oxidative stress response and is critical for pathogenicity of the rice blast fungus *Magnaporthe oryzae*. PLoS Pathog.

[27] Huang K, Czymmek KJ, Caplan JL, Sweigard JA, Donofrio NM (2011). HYR1-mediated detoxification of reactive oxygen species is required for full virulence in the rice blast fungus. PLoS Pathog.

[28] Nicholls S, Straffon M, Enjalbert B, Nantel A, Macaskill S, Whiteway M, Brown AJ (2004). Msn2-and Msn4-like transcription factors play no obvious roles in the stress responses of the fungal pathogen *Candida albicans*. Eukaryot Cell.

[29] Roetzer A, Gregori C, Jennings AM, Quintin J, Ferrandon D, Butler G, Kuchler K, Ammerer G, Schüller C (2008). *Candida glabrata* environmental stress response involves *Saccharomyces cerevisiae* Msn2/4 orthologous transcription factors. Mol Microbiol.

[30] Xiao G, Ying SH, Zheng P, Wang ZL, Zhang S, Xie XQ, Shang Y, St Leger RJ, Zhao GP, Wang C, Feng MG (2012). Genomic perspectives on the evolution of fungal entomopathogenicity in *Beauveria bassiana*. Sci Rep.

[31] Luo Z, Li Y, Mousa J, Bruner S, Zhang Y, Pei Y, Keyhani NO (2015). Bbmsn2 acts as a pH-dependent negative regulator of secondary metabolite production in the entomopathogenic fungus *Beauveria bassiana*. Environ Microbiol.

[32] Liu Q, Ying SH, Li JG, Tian CG, Feng MG (2013). Insight into the transcriptional regulation of Msn2 required for conidiation, multi-stress responses and virulence of two entomopathogenic fungi. Fungal Genet Biol.

[33] Fassler JS, West AH (2011). Fungal Skn7 stress responses and their relationship to virulence. Eukaryot Cell.

[34] Chen LH, Lin CH, Chung KR (2012). Roles for SKN7 response regulator in stress resistance, conidiation and virulence in the citrus pathogen *Alternaria alternata*. Fungal Genet Biol.

[35] Yang Q, Yin D, Yin Y, Cao Y, Ma Z (2015). The response regulator BcSkn7 is required for vegetative differentiation and adaptation to oxidative and osmotic stresses in *Botrytis cinerea*. Mol Plant Pathol.

[36] Jiang C, Zhang S, Zhang Q, Tao Y, Wang C, Xu JR (2015). FgSKN7 and FgATF1 have overlapping functions in as cosporogenesis, pathogenesis and stress responses in *Fusarium graminearum*. Environ Microbiol.

[37] Shang Y, Chen P, Chen Y, Lu Y, Wang C (2015). MrSkn7 controls sporulation, cell wall integrity, autolysis, and virulence in *Metarhizium robertsii*. Eukaryot Cell.

[38] Todd RB, Zhou M, Ohm RA, Leeggangers HA, Visser L, de Vries RP (2014). Prevalence of transcription factors in ascomycete and basidiomycete fungi. BMC Genomics.

[39] Glare T, Caradus J, Gelernter W, Jackson T, Keyhani N, Köhl J, Marrone P, Morin L, Stewart A (2012). Have biopesticides come of age?. Trends Biotechnol.

[40] Ortiz-Urquiza A, Keyhani NO (2013). Action on the surface: entomopathogenic fungi versus the insect cuticle. Insects.

[41] Holder DJ, Keyhani NO (2005). Adhesion of the entomopathogenic fungus *Beauveria* (*Cordyceps*) *bassiana* to substrata. Appl Environ Microbiol.

[42] Pedrini N, Ortiz-Urquiza A, Huarte-Bonnet C, Fan Y, Juárez MP, Keyhani NO (2015). Tenebrionid secretions and a fungal benzoquinone oxidoreductase form competing components of an arms race between a host and pathogen. Proc Natl Acad Sci USA.

[43] He Z, Zhao X, Gao Y, Keyhani NO, Wang H, Deng J, Lu Z, Kan Y, Luo Z, Zhang Y (2020). The fungal mitochondrial membrane protein, BbOhmm, antagonistically controls hypoxia tolerance. Environ Microbiol.

[44] Zhang Y, Zhao J, Fang W, Zhang J, Luo Z, Zhang M, Fan Y, Pei Y (2009). Mitogen-activated protein kinase hog1 in the entomopathogenic fungus *Beauveria bassiana* regulates environmental stress responses and virulence to insects. Appl Environ Microbiol.

[45] Gonźalez-Rubio G, Ferńandez-Acero T, Martín H, Molina M (2019). Mitogen- Activated Protein Kinase Phosphatases (MKPs) in fungal signaling: conservation, function, and regulation. Int J Mol Sci.

[46] Zhao X, Yang X, Lu Z, Wang H, He Z, Zhou G, Luo Z, Zhang Y (2019). MADS-box transcription factor Mcm1 controls cell cycle, fungal development, cell integrity and virulence in the filamentous insect pathogenic fungus *Beauveria bassiana*. Environ Microbiol.

[47] Chen RE, Thorner J (2007). Function and regulation in MAPK signaling pathways: lessons learned from the yeast *Saccharomyces cerevisiae*. Biochim Biophys Acta.

[48] Marín MJ, Flández M, Bermejo C, Arroyo J, Martín H, Molina M (2009). Different modulation of the outputs of yeast MAPK-mediated pathways by distinct stimuli and isoforms of the dual-specificity phosphatase Msg5. Mol Genet Genomics.

[49] Mendenhall MD, Hodge AE (1998). Regulation of Cdc28 cyclin-dependent protein kinase activity during the cell cycle of the yeast *Saccharomyces cerevisiae*. Microbiol Mol Biol Rev.

[50] Golias CH, Charalabopoulos A, Charalabopoulos K (2004). Cell proliferation and cell cycle control: a mini review. Int J Clin Pract.

[51] Huang S, Ingber DE (1999). The structural and mechanical complexity of cell-growth control. Nat Cell Biol.

[52] Nishizawa M, Kanaya Y, Toh-E A (1999). Mouse cyclin-dependent kinase (Cdk) 5 is a functional homologue of a yeast Cdk, pho85 kinase. J Biol Chem.

[53] Liu J, Kipreos ET (2000). Evolution of cyclin-dependent kinases (CDKs) and CDK-activating kinases (CAKs): differential conservation of CAKs in yeast and metazoa. Mol Biol Evol.

[54] Zhang Y, Zhang J, Jiang X, Wang G, Luo Z, Fan Y, Wu Z, Pei Y (2010). Requirement of a mitogen-activated protein kinase for appressorium formation and penetration of insect cuticle by the entomopathogenic fungus *Beauveria bassiana*. Appl Environ Microbiol.

[55] Zhao X, Jiang Y, Wang H, Lu Z, Huang S, Luo Z, Zhang L, Lv T, Tang X, Zhang Y (2023). Fus3/Kss1-MAP kinase and Ste12-like control distinct biocontrol-traits besides regulation of insect cuticle penetration via phosphorylation cascade in a filamentous fungal pathogen. Pest Manag Sci.

[56] Zapater M, Clotet J, Escote X, Posas F (2005). Control of cell cycle progression by the stress-activated Hog1 MAPK. Cell Cycle.

[57] Duch A, de Nadal E, Posas F (2012). The p38 and Hog1 SAPKs control cell cycle progression in response to environmental stresses. FEBS Lett.

[58] Oehlen LJ, McKinney JD, Cross FR (1996). Ste12 and Mcm1 regulate cell cycle-dependent transcription of FAR1. Mol Cell Biol.

[59] Mead J, Bruning AR, Gill MK, Steiner AM, Acton TB, Vershon AK (2002). Interactions of the Mcm1 MADS box protein with cofactors that regulate mating in yeast. Mol Biol Cell.

[60] Chou S, Lane S, Liu H (2006). Regulation of mating and filamentation genes by two distinct Ste12 complexes in *Saccharomyces cerevisiae*. Mol Cell Biol.

[61] Wong Sak Hoi  J, Dumas B (2010). Ste12 and Ste12-like proteins, fungal transcription factors regulating development and pathogenicity. Eukaryot Cell.

[62] Shang J, Shang Y, Tang G, Wang C (2021). Identification of a key G-protein coupled receptor in mediating appressorium formation and fungal virulence against insects. Sci China Life Sci.

[63] Zhao X, Luo T, Huang S, Peng N, Yin Y, Luo Z, Zhang Y (2021). A novel transcription factor negatively regulates antioxidant response, cell wall integrity and virulence in the fungal insect pathogen. Beauveria bassiana Environ Microbiol.

[64] Wang H, He Z, Luo L, Zhao X, Lu Z, Luo T, Li M, Zhang Y (2018). An aldo-keto reductase, Bbakr1, is involved in stress response and detoxification of heavy metal chromium but not required for virulence in the insect fungal pathogen. Beauveria bassiana Fungal Genet Biol.

[65] Ma JC, Zhou Q, Zhou YH, Liao XG, Zhang YJ, Jin D, Pei Y (2009). The size and ratio of homologous sequence to non-homologous sequence in gene disruption cassette influences the gene targeting efficiency in *Beauveria bassiana*. Appl Microbiol Biotechnol.

[66] Yang Z, Jiang H, Zhao X, Lu Z, Luo Z, Li X, Zhao J, Zhang Y (2017). Correlation of cell surface proteins of distinct *Beauveria bassiana* cell types and adaption to varied environment and interaction with the host insect. Fungal Genet Biol.

[67] Bähler J, Wu JQ, Longtine MS, Shah NG, Mckenzie A, Steever AB, Wach A, Philippsen P, Pringle JR (1998). Heterologous modules for efficient and versatile PCR-based gene targeting in *Schizosaccharomyces pombe*. Yeast.

[68] Liao XG, Fang WG, Zhang YJ, Fan YH, Wu XW, Zhou Q, Pei Y (2008). Characterization of a highly active promoter, PBbgpd. Beauveria bassiana Curr Microbiol.

[69] Wang Y, An C, Zhang X, Yao J, Zhang Y, Sun Y, Yu F, Amador DM, Mou Z (2013). The *Arabidopsis* elongator complex subunit2 epigenetically regulates plant immune responses. Plant Cell.

[70] Fan G, Zheng H, Zhang K, Devi Ganeshan V, Opiyo SO, Liu D, Li M, Li G, Mitchell TK, Yun Y, Wang Z, Lu GD (2020). Fghtf1 regulates global gene expression towards aerial mycelium and conidiophore formation in the cereal fungal pathogen *Fusarium graminearum*. Appl Environ Microbiol.

[71] Chung D, Barker BM, Carey CC, Merriman B, Werner ER, Lechner BE, Dhingra S, Cheng C, Xu W, Blosser SJ, Morohashi K, Mazurie A, Mitchell TK, Haas H, Mitchell AP, Cramer RA (2014). ChIP-seq and in vivo transcriptome analyses of the *Aspergillus fumigatus* SREBP SrbA reveals a new regulator of the fungal hypoxia response and virulence. PLoS Pathog.

[72] Lohse M, Bolger AM, Nagel A, Fernie AR, Lunn JE, Stitt M, Usadel B (2012). RobiNA: a user-friendly, integrated software solution for RNA-Seq-based transcriptomics. Nucleic Acids Res.

[73] Bailey TL, Boden M, Buske FA, Frith M, Grant CE, Clementi L, Ren J, Li WW, Noble WS (2009). MEME SUITE: tools for motif discovery and searching. Nucleic Acids Res.

[74] Audic S, Claverie JM (1997). The significance of digital gene expression profiles. Genome Res.

[75] Luo X, Keyhani NO, Yu X, He Z, Luo Z, Pei Y, Zhang Y (2012). The MAP kinase Bbslt2 controls growth, conidiation, cell wall integrity, and virulence in the insect pathogenic fungus *Beauveria bassiana*. Fungal Genet Biol.

[76] Kinoshita E, Kinoshita-Kikuta E, Takiyama K, Koike T (2006). Phosphate-binding tag, a new tool to visualize phosphorylated proteins. Mol Cell Proteomics.

[77] Yin Z, Feng W, Chen C, Xu J, Li Y, Yang L, Wang J, Liu X, Wang W, Gao C, Zhang H, Zheng X, Wang P, Zhang Z (2020). Shedding light on autophagy coordinating with cell wall integrity signaling to govern pathogenicity of *Magnaporthe oryzae*. Autophagy.

[78] Jensen EC (2013). Quantitative analysis of histological staining and fluorescence using ImageJ. Anat Rec (Hoboken).

[79] Southwest University. *Beauveria bassiana* Transcriptome or Gene expression. NCBI Bioproject accession: PRJNA868476. https://www.ncbi.nlm.nih.gov/bioproject/?term=PRJNA868476 (2022).

[80] Southwest University. *Beauveria bassiana* OsrR123 ChIP-seq. NCBI Bioproject accession: PRJNA883178. https://www.ncbi.nlm.nih.gov/bioproject/?term=PRJNA883178 (2022).

