# Supplementary material for: A network of transcription factors in complex with a regulating cell cycle cyclin orchestrates fungal oxidative stress responses
Source: BMC Biol. 2024 Apr 12;22:81. doi: 10.1186/s12915-024-01884-3 (PMC11015564; doi:10.1186/s12915-024-01884-3)
Supplement: Supplementary file 1 — Additional file1: Fig S1. Sensitivities of BbOhmm reverse complement strains to oxidative stress. The reverse complement strains were generated by introduction of BbOhmm with different length of promotor sequences into ΔBbOhmm. P + number indicate length of promotor sequence. “P1500+ mutated sites” indicate the element ‘ATATC’ in 1500 bp promotor sequences of BbOhmm was mutated to CCCTC. Two μL conidial suspensions (1×10^7^ cells/ mL−1) were spot-inoculated onto CZM plates supplemented with H2O2 (5.76 mM) and incubated at 26℃ for 7 days. Fig S2. Identification of transcription factors of BbOhmm in response to oxidative stress. (A) DNA-binding domain-contained clones that were screened from the yeast-one hybrid library. (B) Verification of transcription control of BbOhmm by the screened DNA-binding domain-contained proteins in yeast. DNA-binding domain-coding region of the protein was cloned into pGADT7-Rec, which was introduced into the Y1HGold strain harboring pAbAi-bait (vectoring tandem repeat of three copies of the ‘ATATC’ element in BbOhmm promoter). The transformants were cultured on Leu-free SD agar or Leu-free SD agar containing 125 ng / mL Aureobasidin A (AbA) to examine colony growth. The yeast cells transformed with vector p53-AbAi and pGADT7-Recp53, or with the pGADT7 AD vector containing the DNA-binding sequence of candidate proteins and a blank vector (p53-AbAi) vector, were used as positive or negative controls, respectively. (C) Transcriptional activation assays in yeast. Yeast strain Y2HGold carrying fusion cassettes of the GAL4 DNA-binding domain (BDGal4; negative control), the GAL4 DNA-binding and activation domains (BDGal4-ADGal4; positive control), or the GAL4 DNA-binding and the indicated TF (BDGal4::TF) were cultured on Trp-free SD plates containing 0.5 mM X-α-gal and 125 ng/ mL AbA at 30oC for 3 days. (D) Domain organization of three TFs, BbOsrR1, BbOsrR2 and BbOsrR3. (E) RT-qPCR analysis of BbOsrR1, BbOsrR2, BbOsrR3 and BbClp1 expression in wi [file 12915_2024_1884_MOESM1_ESM.pdf]

A network of transcription factors in complex with a regulating cell cycle cyclin orchestrates fungal oxidative stress responses

Additional file 1:

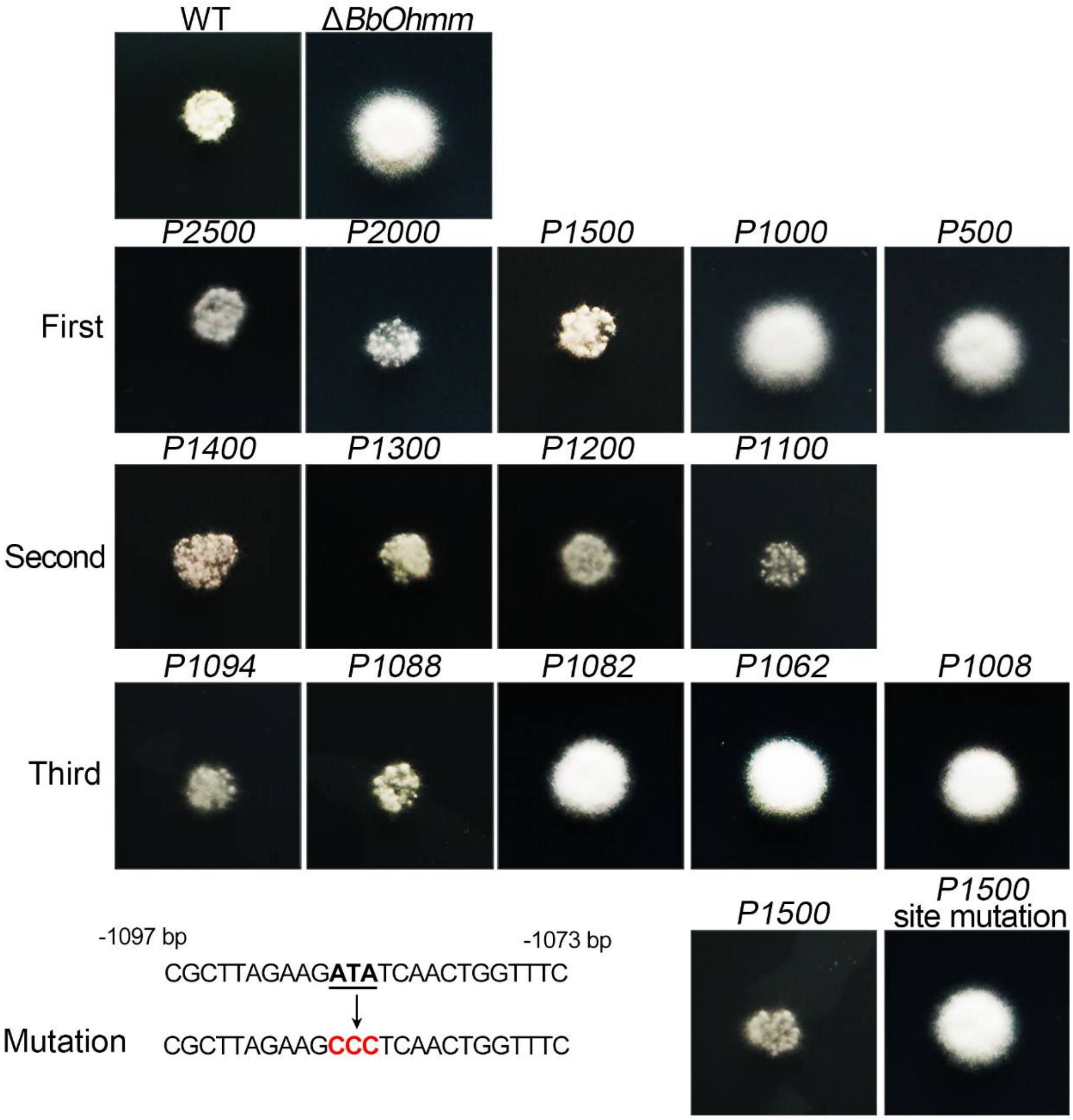

Fig S1. Sensitivities of *BbOhmm* reverse complement strains to oxidative stress. The reverse complement strains were generated by introduction of *BbOhmm* with different length of promotor sequences into Δ*BbOhmm*. P + number indicate length of promotor sequence. "P1500+ mutated sites" indicate the element 'ATATC' in 1500 bp promotor sequences of *BbOhmm* was mutated to CCCTC. Two μL conidial suspensions (1×10^7 cells/ mL^{-1}) were spot-inoculated onto CZM plates supplemented with H_2O_2 (5.76 mM) and incubated at 26°C for 7 days.

A DNA-binding domain-contained clones that were screened from the yeast one-hybrid libraries

Gene ID	Description	DNA-binding type
BBA_04239	negative acting factor	GAL4-like Zn_2Cys_6
BBA_01981	fungal specific transcription factor	GAL4-like Zn_2Cys_6
BBA_01499	nitrate assimilation regulatory protein nirA	GAL4-like Zn_2Cys_6
BBA_06193	transcription factor Opi1	Opil
BBA_00237	Transcription factor	GAL4-like Zn_2Cys_6
BBA_05389	WD domain-containing protein	WD40
BBA_04821	RING-13 protein	zf-C3HC4.2

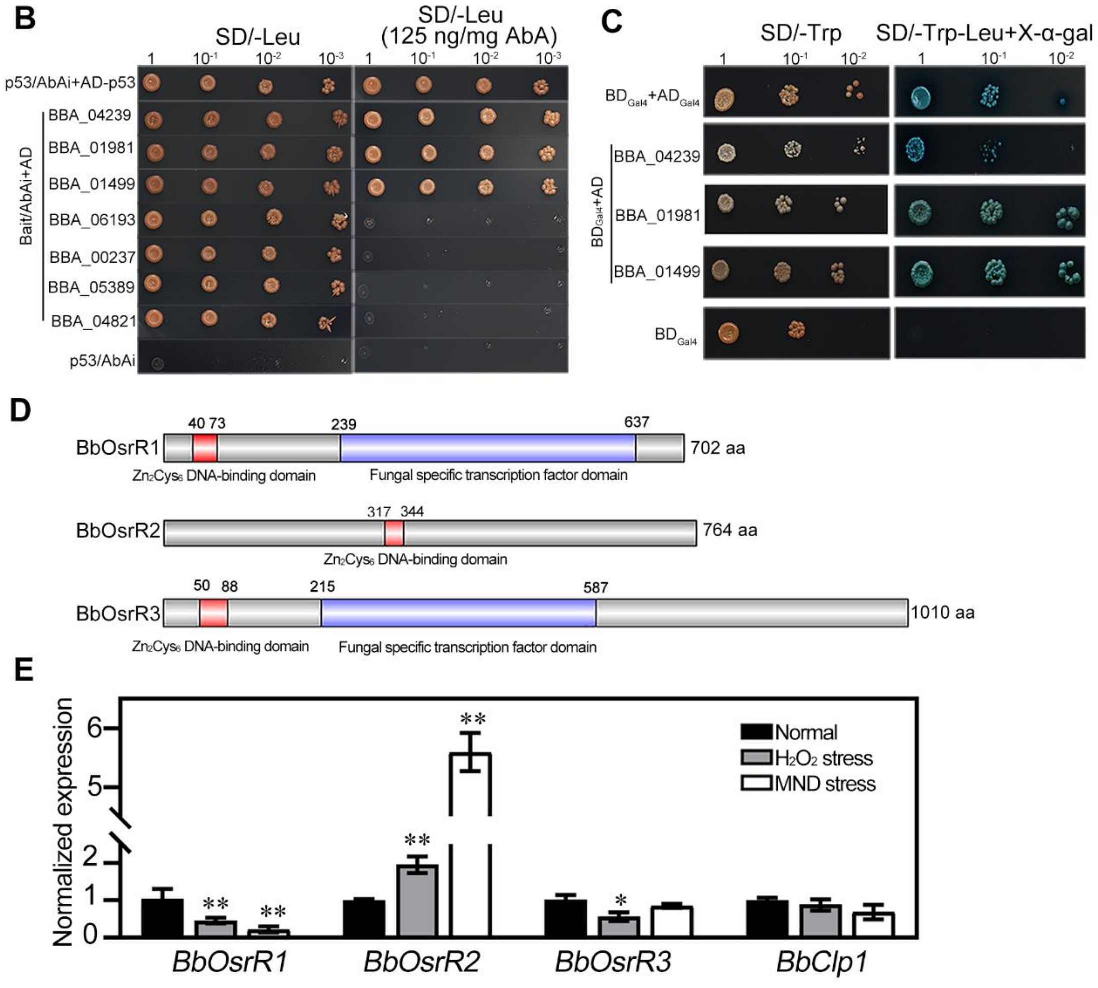

Fig S2. Identification of transcription factors of *BbOhmm* in response to oxidative stress. (A) DNA-binding domain-contained clones that were screened from the yeast one-hybrid library. (B) Verification of transcription control of *BbOhmm* by the screened DNA-binding domain-contained proteins in yeast. DNA-binding domain-coding region of the protein was cloned into pGADT7-Rec, which was introduced into the Y1HGold strain harboring pAbAi-bait (vectoring tandem repeat of three copies of the ‘ATATC’

element in *BbOhmm* promoter). The transformants were cultured on Leu-free SD agar or Leu-free SD agar containing 125 ng/ mL Aureobasidin A (AbA) to examine colony growth. The yeast cells transformed with vector p53-AbAi and pGADT7-Recp53, or with the pGADT7 AD vector containing the DNA-binding sequence of candidate proteins and a blank vector (p53-AbAi) vector, were used as positive or negative controls, respectively. (C) Transcriptional activation assays in yeast. Yeast strain Y2HGold carrying fusion cassettes of the GAL4 DNA-binding domain (BDGal4; negative control), the GAL4 DNA-binding and activation domains (BDGal4-ADGal4; positive control), or the GAL4 DNA-binding and the indicated TF (BDGal4::TF) were cultured on Trp-free SD plates containing 0.5 mM X-α-gal and 125 ng/ mL AbA at 30ºC for 3 days. (D) Domain organization of three TFs, BbOsrR1, BbOsrR2 and BbOsrR3. (E) RT-qPCR analysis of *BbOsrR1*, *BbOsrR2*, *BbOsrR3* and *BbClp1* expression in wild type strain under unstressed and H_2O_2 (4 mM) or menadione (MND, 60 μM)-stressed conditions for 30 min. The asterisks (*) and (**) in the column charts denote $P < 0.05$ and $P < 0.01$ for the stressed conditions versus the unstressed condition (¼ SDY) (t-test), respectively.

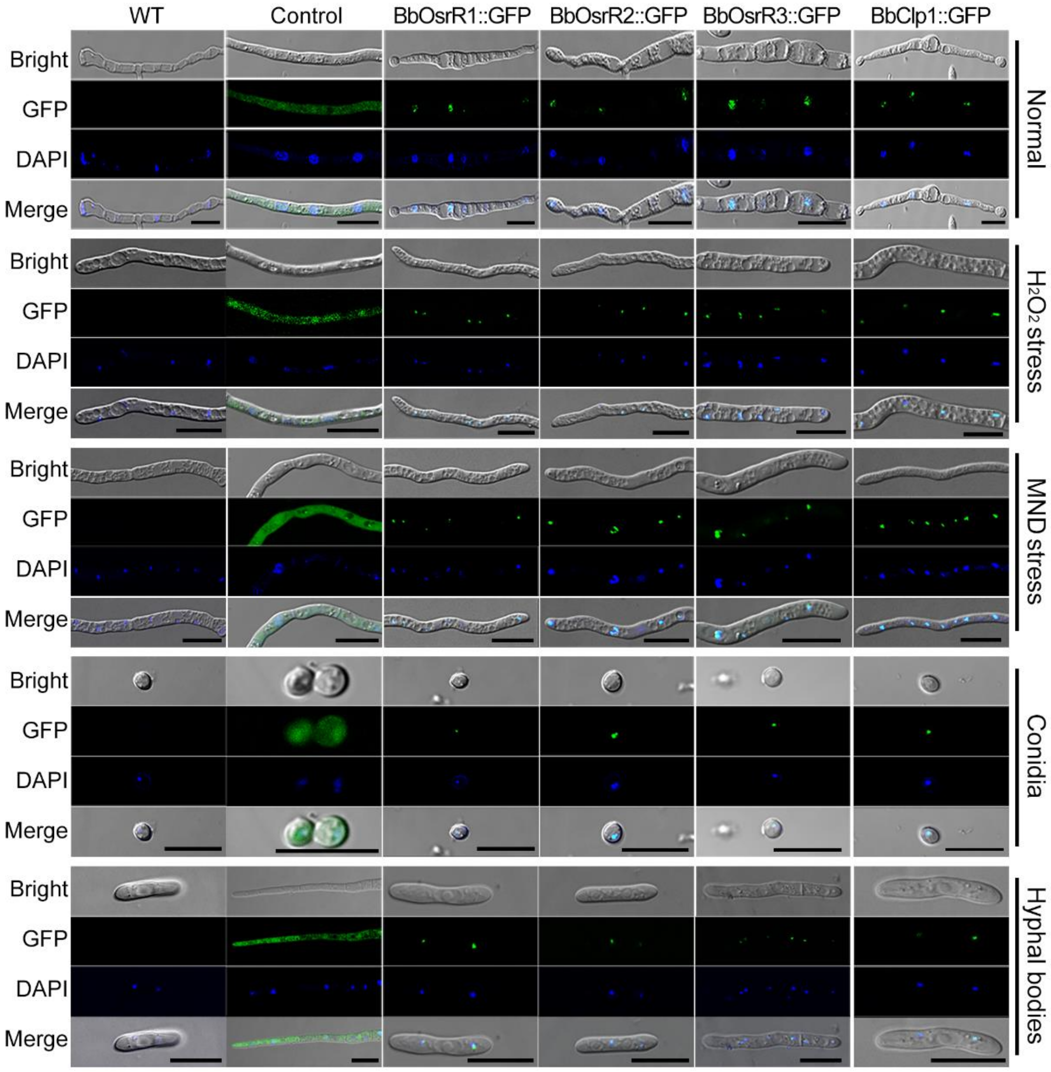

Fig S3. Localization examination of the three TFs and BbClp1 in *B. bassiana*. GFP signals of BbOsrR1::eGFP, BbOsrR2::eGFP, BbOsrR3::eGFP and BbClp1::eGFP are distributed in nuclei of conidia, germlings without or with exposure to oxidative stress (4 mM H_2O_2 or 60 μM menadione for 30 min), and *in vivo* blastospores (derived from infected insect), which was stained with florescent DAPI (4', 6-diamidino-2-phenylindole). Scale bar = 10 μm.

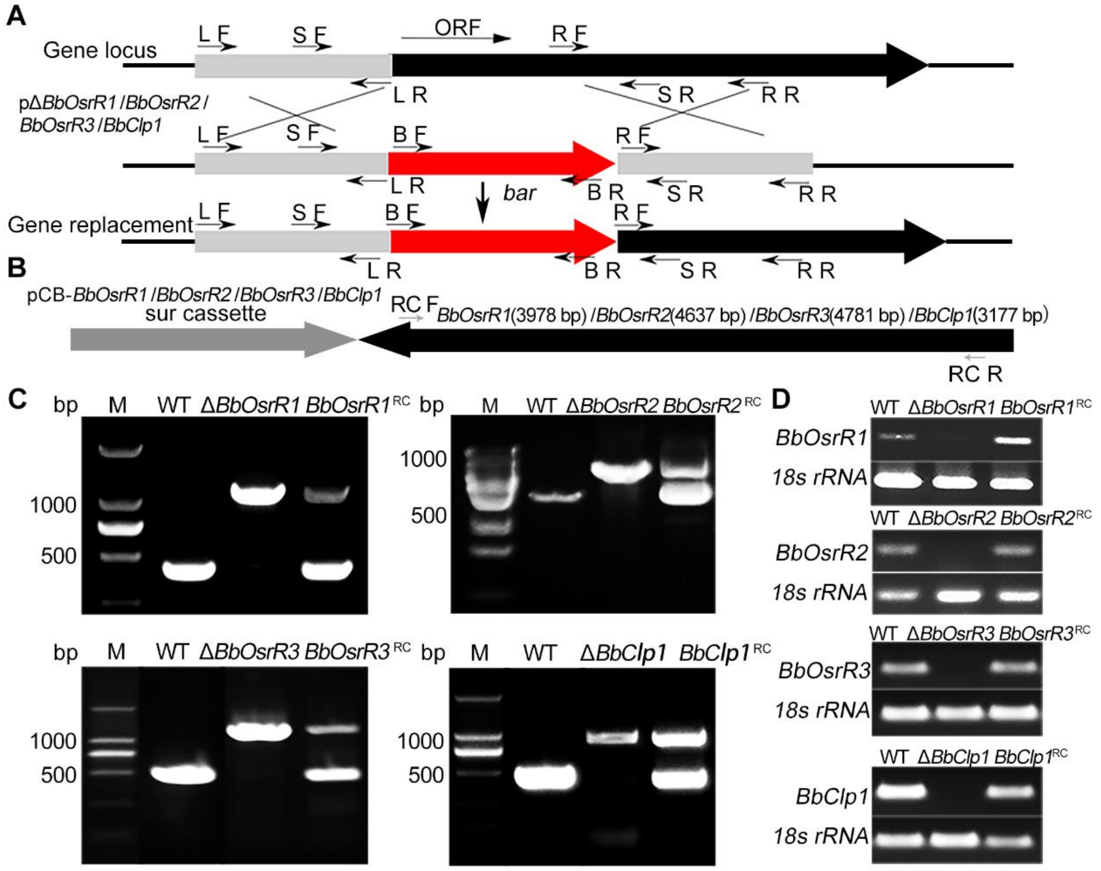

Fig S4. Molecular manipulations of the targeted gene disruption and complementation. (A) Target gene locus and gene replacement vector pΔ*BbOsrR1*/ *BbOsrR2*/ *BbOsrR3*/ *BbClp1*. The gene replacement vector contains *bar* cassette flanked by 5' and 3' border sequences of target gene. Homologous recombination (cross over event marked by "X") resulting in a region of target gene was replaced by the *bar* cassette. Arrow indicates the orientation of transcription in the ORF. (B) Target gene complementation vector, pCB-*BbOsrR1*/ *BbOsrR2*/ *BbOsrR3*/ *BbClp1*, containing the sulfonylurea resistance marker (*sur*). (C) PCR analysis of wild type strain (WT), Δ*BbOsrR1*/ Δ*BbOsrR2*/ Δ*BbOsrR3*/ Δ*BbClp1*, and complemented mutant (*BbOsrR1*^{RC}/ *BbOsrR2* ^{RC}/ *BbOsrR3* ^{RC}/ *BbClp1*^{RC}). Desired integration events were confirmed by PCR using primers S-F and S-R. (D) RT-PCR confirmation of loss of gene expression in the gene disruption mutant and recovery in the complemented strain (Comp). Total RNA was isolated as described in the Methods section. Expression of gene was examined using *18S rRNA* as the reference gene in wild type (WT), gene disruption mutant, and reverse complemented mutant strains.

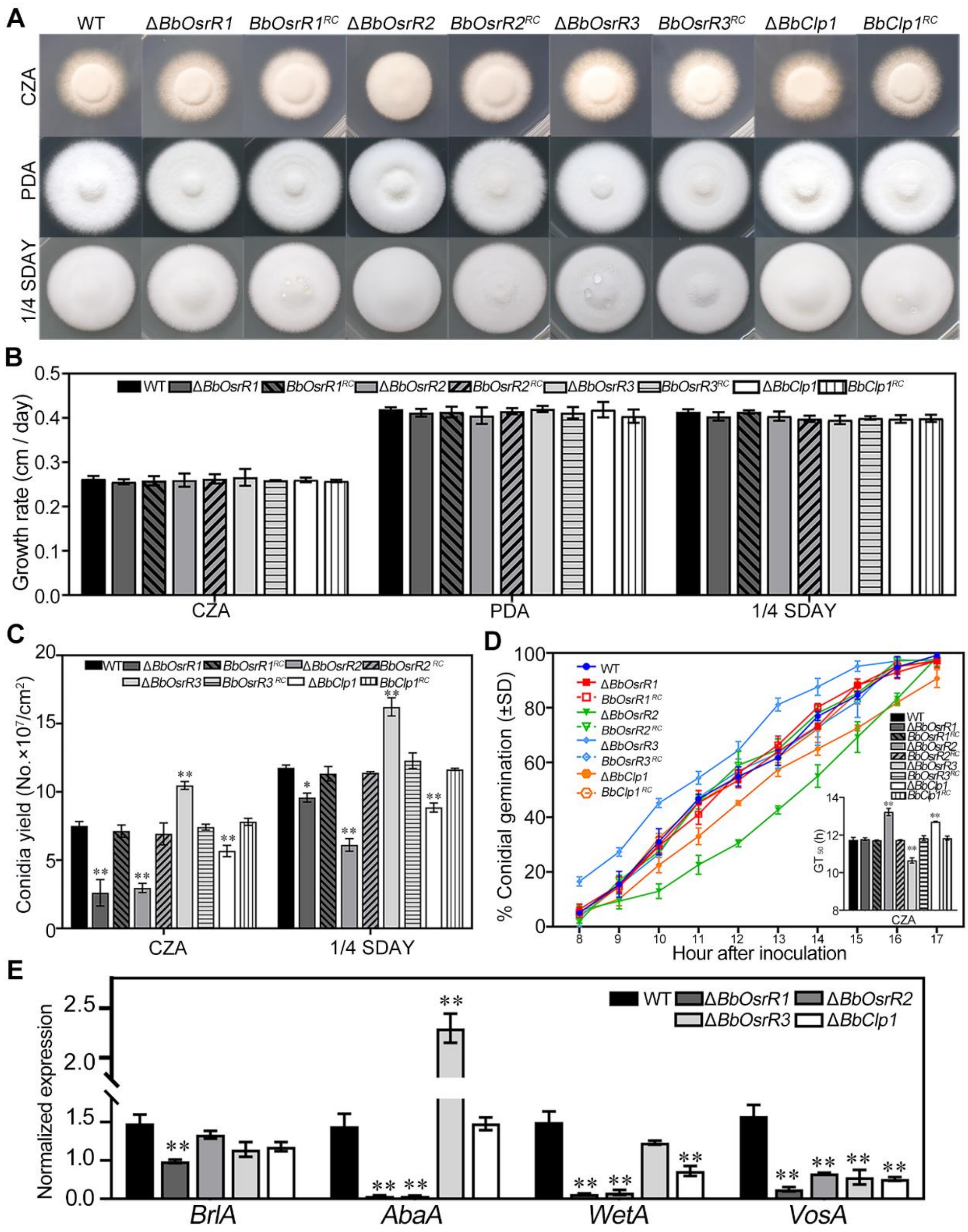

Fig S5. Colony growth of wild type (WT), gene disruption mutant and complemented strains. (A) Colony grown on Czapek-Dox agar (CZA), PDA and ¼ SDAY at 26°C for 7 days. (B) Growth rate of the colony. Colony diameter was measured daily after 3 days of inoculation using cross method with a vernier caliper. Colony growth rate was evaluated using linear-regression analysis. Error bars indicate SDs. (C) Conidial yield of the fungal strains on CZA and ¼ SDAY plates after 14 days at 26°C. (D) Conidial

germination rate. Conidial germination was monitored over the indicated time course via microscopic analysis of samples. Conidia were considered germinated when the germ tube was equal in length to the diameter of the conidia. At least 300 conidia were examined and the experiment repeated using three independent batches of conidia. The time required to achieve 50% germination of conidia (GT_{50}) was estimated by modeling analysis of the germination trend of each strain over the time of incubation. (E) RT-qPCR analysis of *BrlA*, *AbaA*, *WetA* and *VosA* at conidiation stage (5 days on ¼ SDAY). The average values with standard deviation (±SD) of triplicated experiments are shown in (C, D and E). The asterisks (*) and (**) in (C) and D) denote $P < 0.05$ and $P < 0.01$ for the indicated fungal strains versus the wild type strain (WT) (t-test), respectively.

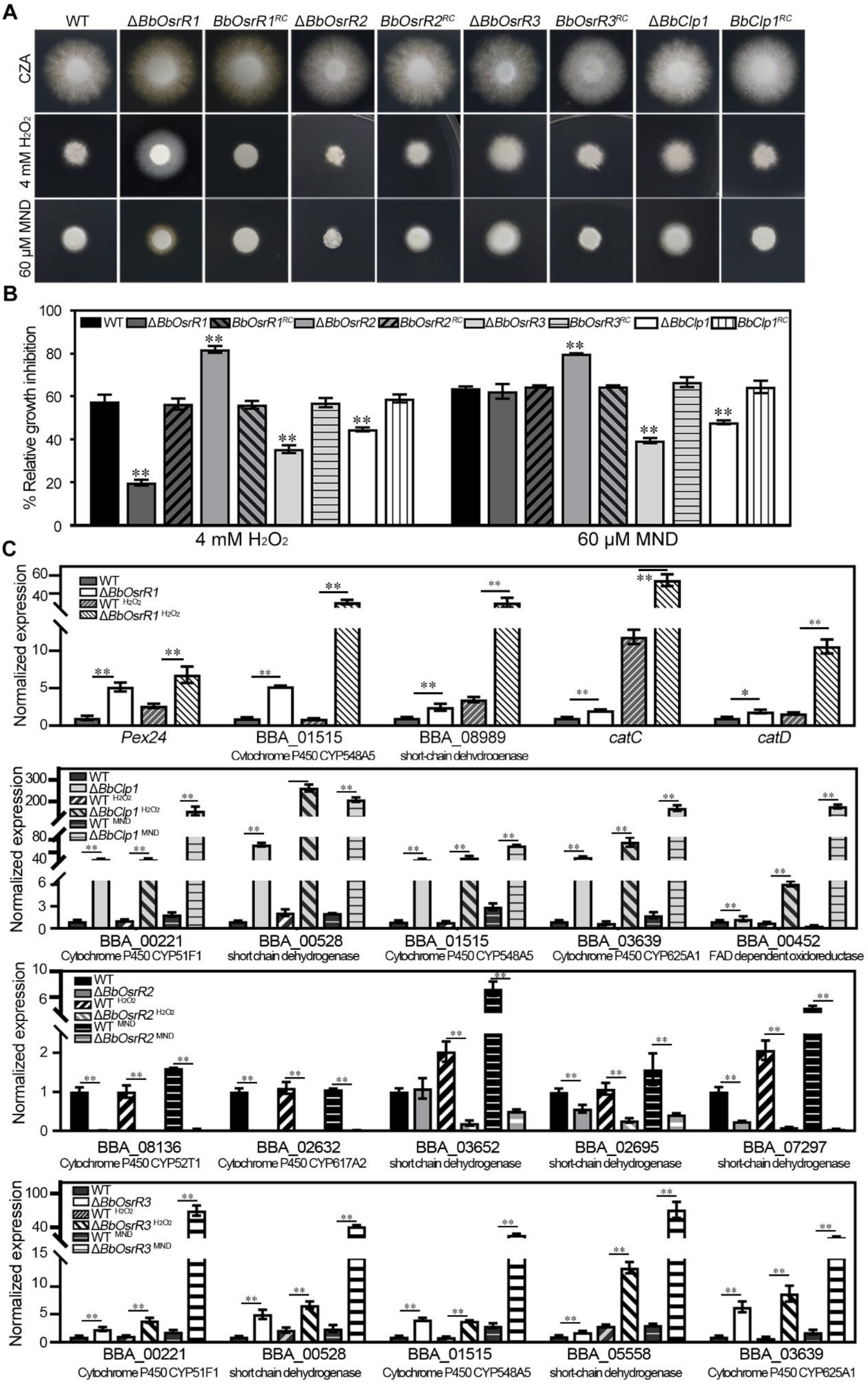
A
WT
ΔBbOsrR1
BbOsrR1RC
ΔBbOsrR2
BbOsrR2RC
ΔBbOsrR3
BbOsrR3RC
ΔBbClp1
BbClp1RC
CZA
4 mM H2O2
60 μM MND
B
% Relative growth inhibition
4 mM H2O2
60 μM MND
C
Normalized expression
Pex24
BBA_01515
Cytochrome P450 CYP548A5
BBA_08989
short-chain dehydrogenase
catC
catD
BBA_00221
Cytochrome P450 CYP51F1
BBA_00528
short chain dehydrogenase
BBA_01515
Cytochrome P450 CYP548A5
BBA_03639
Cytochrome P450 CYP625A1
BBA_00452
FAD dependent oxidoreductase
BBA_08136
Cytochrome P450 CYP52T1
BBA_02632
Cytochrome P450 CYP617A2
BBA_03652
short chain dehydrogenase
BBA_02695
short-chain dehydrogenase
BBA_07297
short-chain dehydrogenase
BBA_00221
Cytochrome P450 CYP51F1
BBA_00528
short chain dehydrogenase
BBA_01515
Cytochrome P450 CYP548A5
BBA_05558
short-chain dehydrogenase
BBA_03639
Cytochrome P450 CYP625A1

Fig S6. Sensitivities of the wild type and mutant strains to H_2O_2 and menadione (MND) on CZA and expression patterns of key antioxidant/ detoxification genes. (A) Vegetative growth of the wild type and mutant strains on CZA and CZA containing 4 mM H_2O_2 or 60 μM MND for 7 days. (B) Calculated relative growth inhibition (RGI) of *B. bassiana* isolates challenged with oxidative stressors for 7 days as described in Methods section. (C) RT-qPCR analysis of key antioxidant/ detoxification genes from RNA-seq datasets in the CZB-cultured mutants either under no-stress and H_2O_2 (4 mM)- or menadione (60 μM)-stress conditions for 30 min as compared to wild type strain. The asterisks (**) in the column charts denote $P < 0.01$ for the indicated fungal strains versus the WT (t-test).

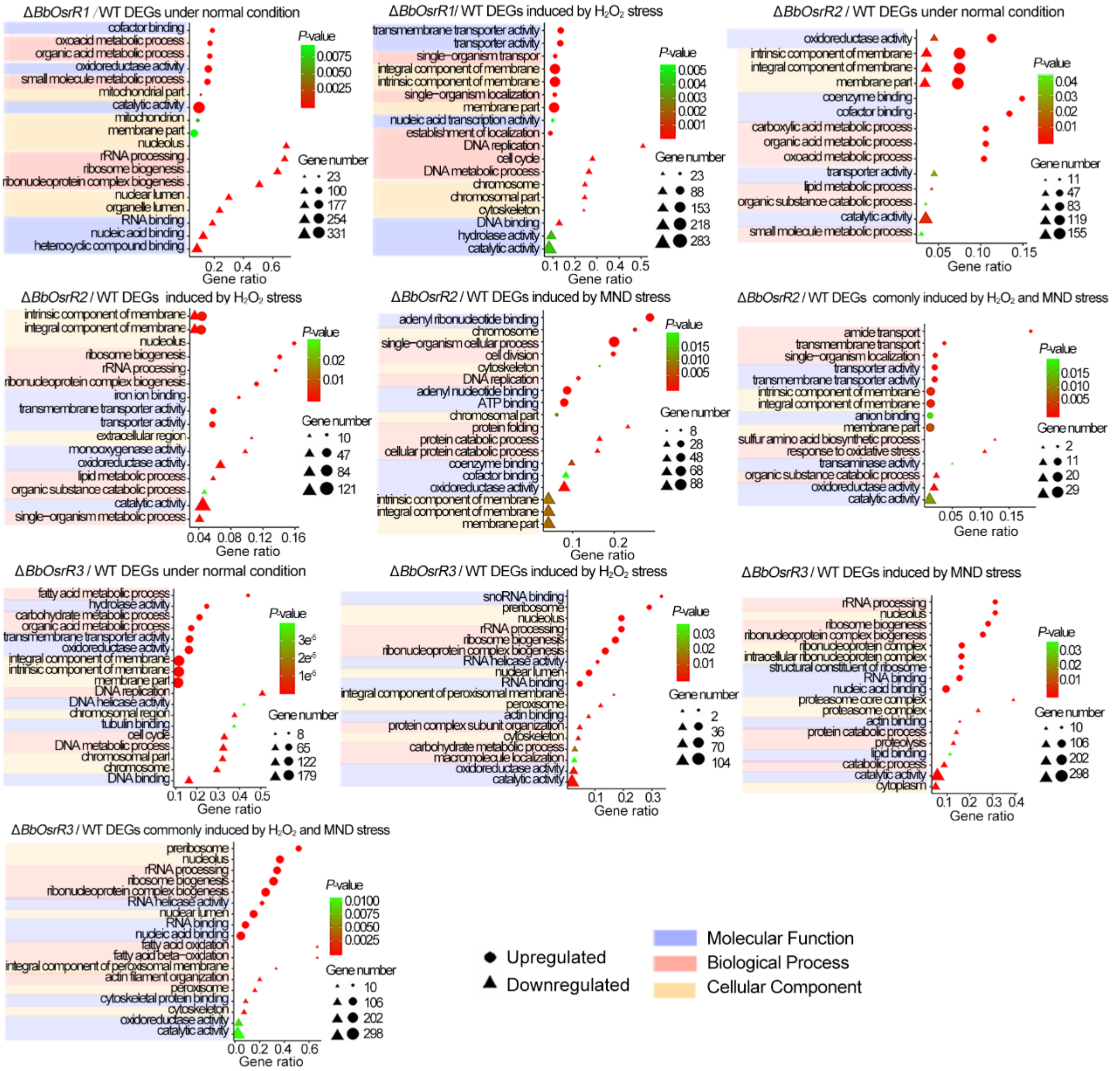

Fig S7. GO enrichment analysis of Δ*BbOsrR1*, Δ*BbOsrR2* or Δ*BbOsrR3* versus WT DEGs under normal condition (¼ SDY), specially induced by H_2O_2 (4 mM) or/and menadione (MND, 60 μM) stresses for 30 min, respectively, and those DEGs commonly induced by H_2O_2 and MND.

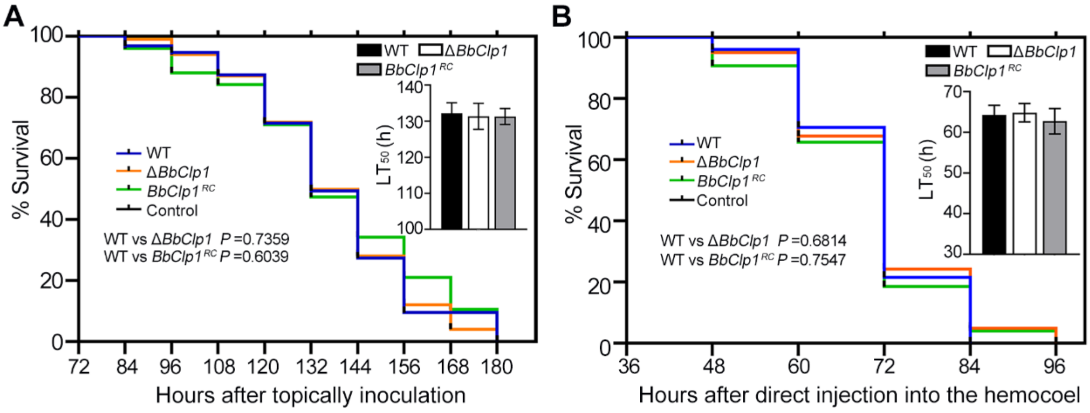

Fig S8. Insect bioassays of the wild type (WT), *BbClp1* disruption mutant and its' reverse complementation strains. (A) Survival of *G. mellonella* larvae following topical infection with the spore suspensions (5 × 10^6 conidia/ mL suspensions). (B) Survival of the larvae following injection into the second proleg with 2 μL of 1 × 10^6 conidia/ mL suspensions. Control insects were treated with sterile water. The survival data were plotted as Kaplan–Meyer curves and difference of gene disruption strains from the WT and its reverse was analyzed using a log rank test. The mean lethal time to kill 50% of targets (LT_{50}) was estimated using the SPSS 17.0 program. The experiments were repeated twice.

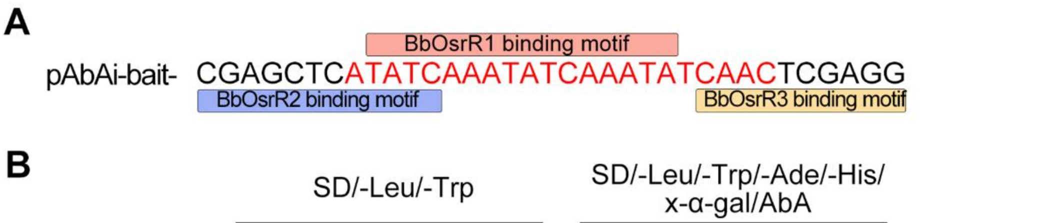

RNA-seq (Normal)	ΔBbOsrR1	ΔBbOsrR2	ΔBbOsrR3	ΔBbClp1
ΔBbOsrR1	1.00	0.84	0.51	0.57
ΔBbOsrR2	0.84	1.00	0.67	0.73
ΔBbOsrR3	0.51	0.67	1.00	0.76
ΔBbClp1	0.57	0.73	0.76	1.00

RNA-seq (H_2O_2 - stress)	ΔBbOsrR1	ΔBbOsrR2	ΔBbOsrR3	ΔBbClp1
ΔBbOsrR1	1.00	0.80	0.84	0.78
ΔBbOsrR2	0.80	1.00	0.80	0.77
ΔBbOsrR3	0.84	0.80	1.00	0.95
ΔBbClp1	0.78	0.77	0.95	1.00

RNA-seq (MND - stress)	ΔBbOsrR2	ΔBbOsrR3	ΔBbClp1
ΔBbOsrR2	1.00	0.74	0.79
ΔBbOsrR3	0.74	1.00	0.94
ΔBbClp1	0.79	0.94	1.00

Fig S9. Scanning of binding motifs BbOsrR1, 2, in and 3 the three-copy tandem repeat of the 'ATATC' used for yeast-one hybrid screening (A), yeast two-hybrid tests of interactions between BbOsrR2, BbOsrR3 and BbClp1 (B), and correlation of three TFs and BbClp1-mediated genes under the normal or oxidative stress conditions (C). Gold cells co-transformed with the pGBKT7-BbClp1 and pGADT7-BbOsrR2/BbOsrR3 grown on the medium (SD-His-Ade-Leu-Trp) with 0.5 mM X-α-gal and 350 ng/ mL AbA for 3 d. The yeast cells transformed with vector p53-AbAi and pGADT7-Rec-p53 vector (Clontech) or with a blank vector p53-AbAi only were used as positive or negative controls, respectively. Transcriptomic data controlled by those factors were comparatively analyzed using an intergroup correlation analysis (ICA) with the OmicShare ICA tools2 (https://www.omicshare.com/tools). Pearson correlation coefficient was calculated and indicated.
