# Supplementary material for: A network of transcription factors in complex with a regulating cell cycle cyclin orchestrates fungal oxidative stress responses
Source: BMC Biol. 2024 Apr 12;22:81. doi: 10.1186/s12915-024-01884-3 (PMC11015564; doi:10.1186/s12915-024-01884-3)
Supplement: Supplementary file 3 — Additional file3: Original images of EMSA, Western blot and PCR gel. [file 12915_2024_1884_MOESM3_ESM.pdf]

A network of transcription factors in complex with a regulating cell cycle cyclin orchestrates fungal oxidative stress responses

Additional file 3:

Original images of EMSA, Western blot, and PCR gel

Fig 1A

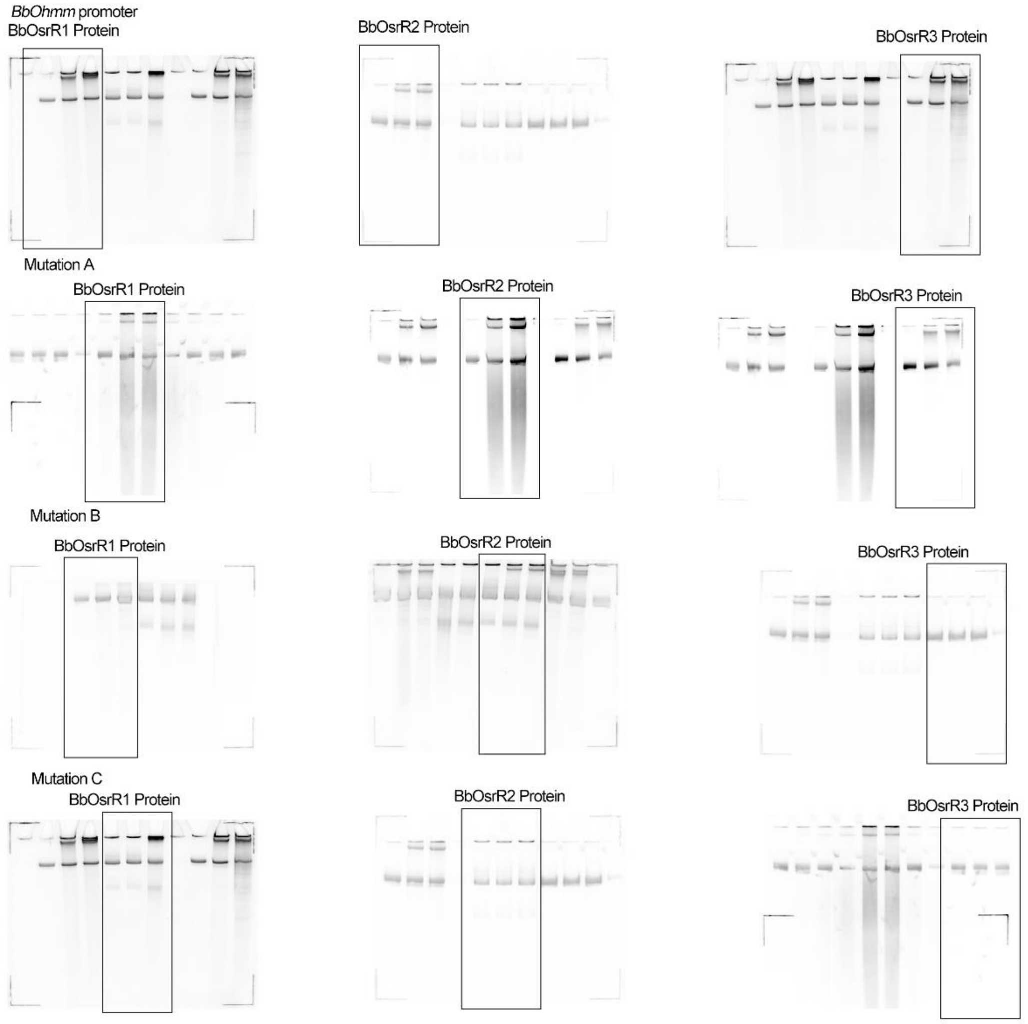

Original EMSA gel in Figure 1.

Fig 3D

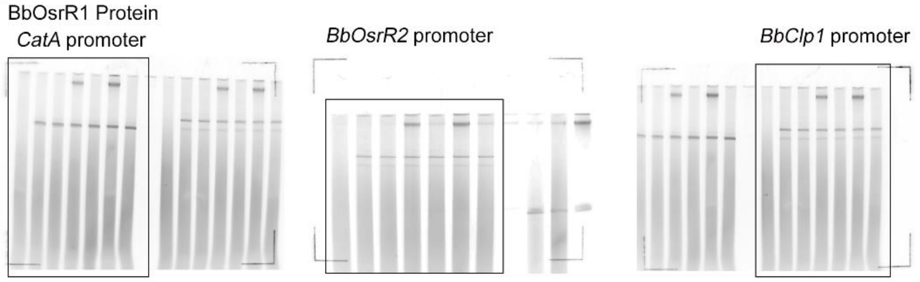

Fig 5D

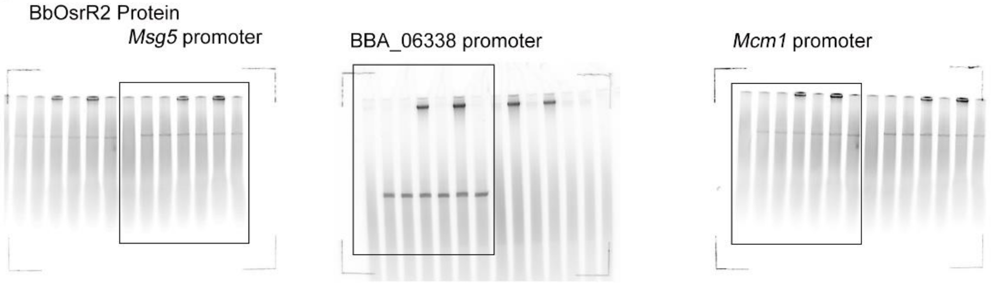

Fig 6D

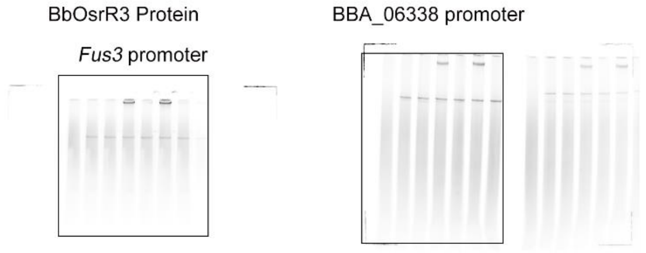

Original EMSA gel in Figure 3/5/6.

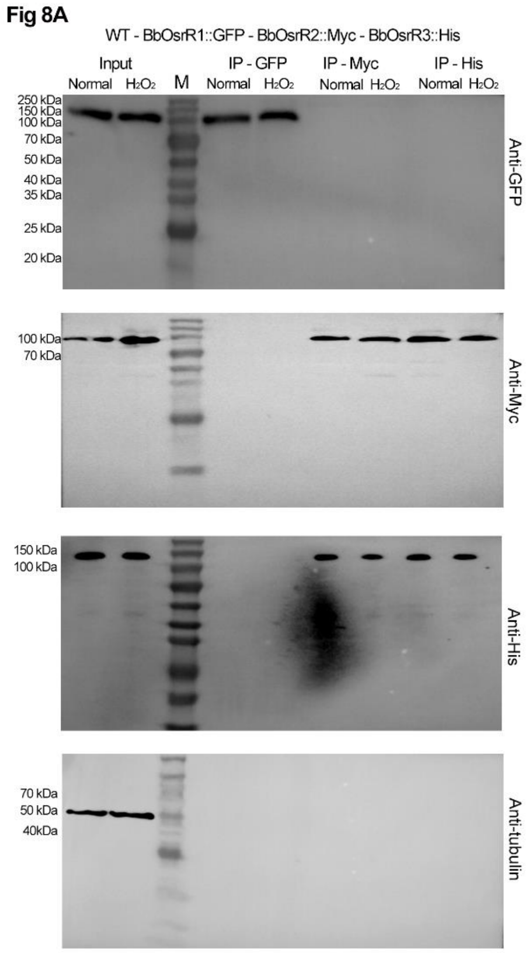

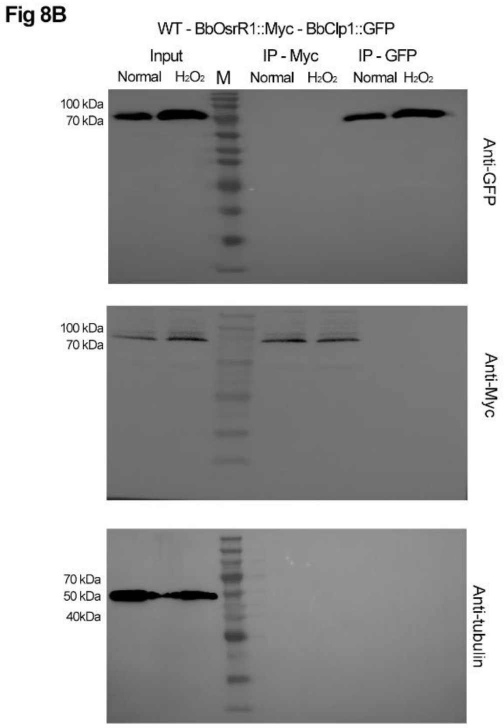

Original Western blots in Figure 8A/B.

Fig 8C

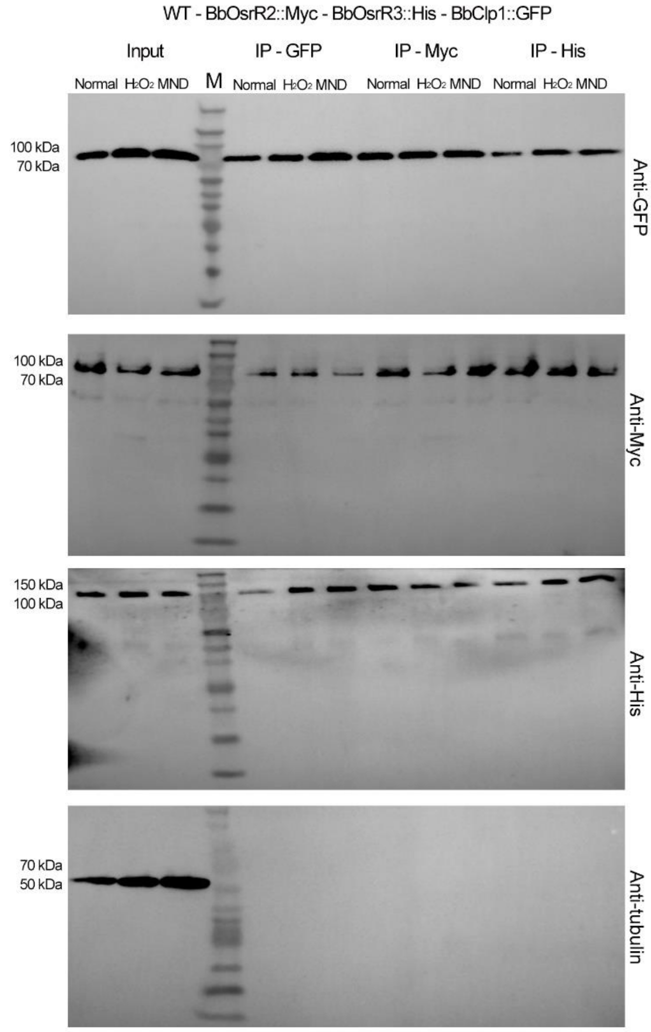

Fig 8D

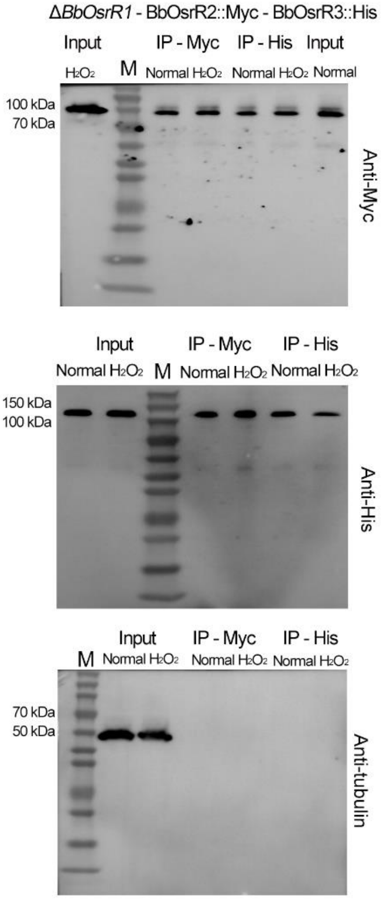

Original Western blots in Figure 8C/D.

Fig 8E

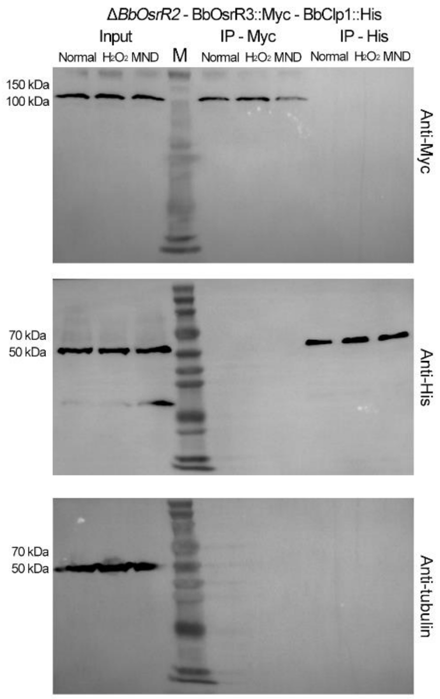

Fig 8F

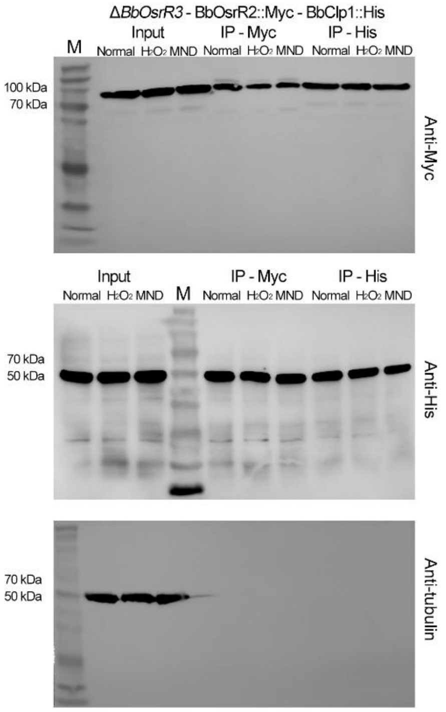

Fig 8G

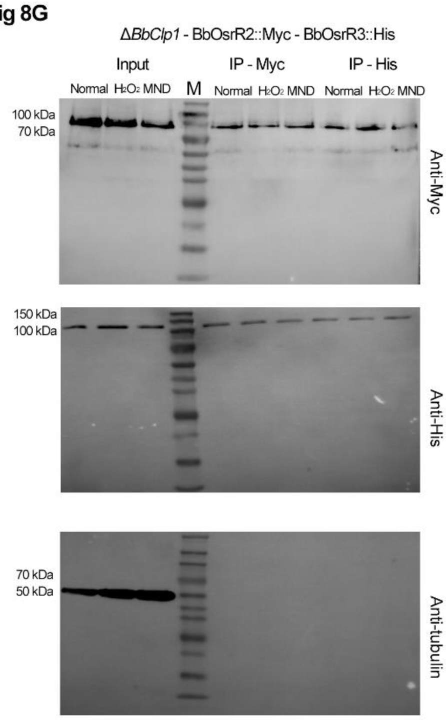

Fig 8H

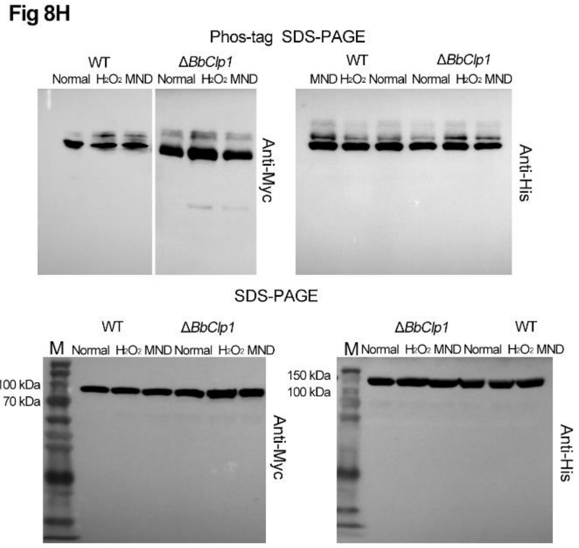

Original Western blots in Figure 8E/F/G/H.

Fig S3C

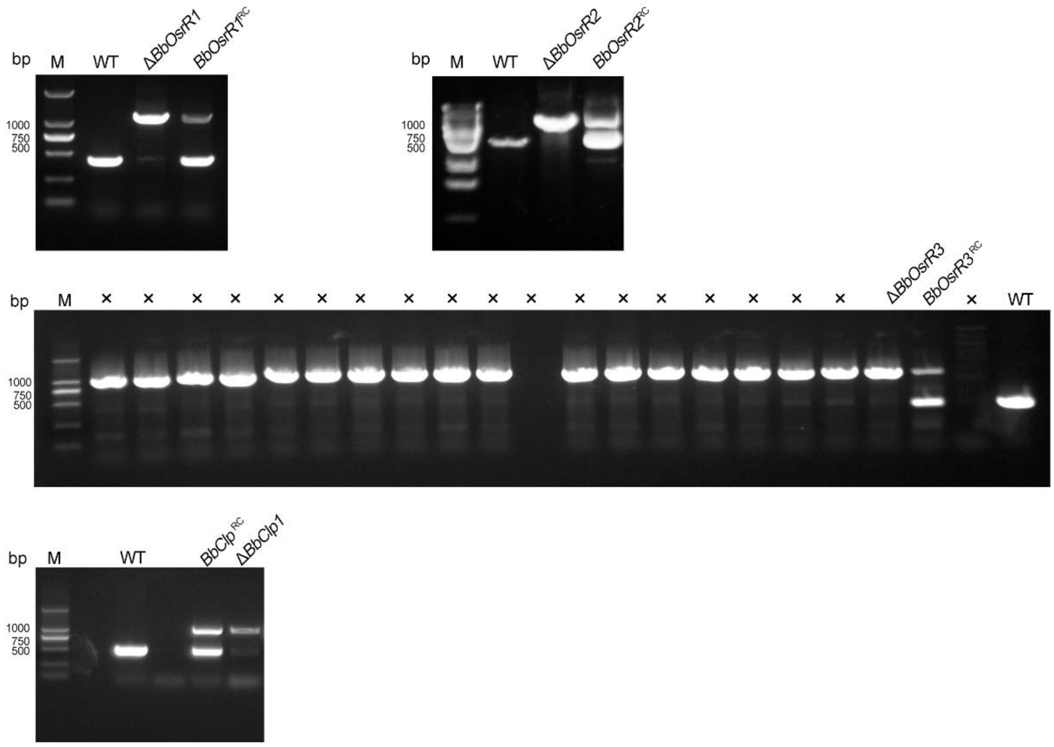

Fig S3D

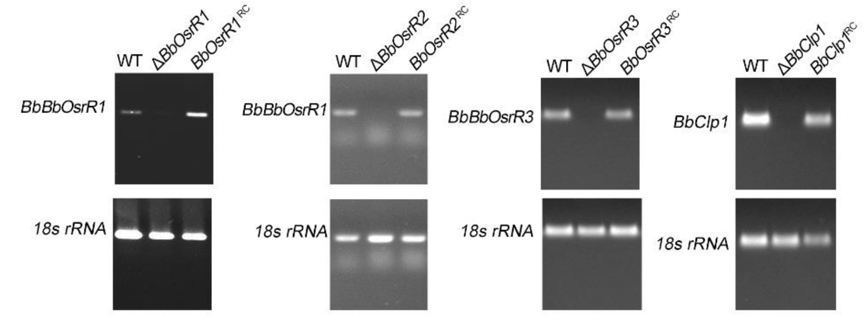

Original PCR gel in Figure S3C/D.
